# Optimizing the immunogenicity of HIV prime-boost DNA-MVA-rgp140/GLA vaccines in a phase II randomized factorial trial design

**DOI:** 10.1371/journal.pone.0206838

**Published:** 2018-11-29

**Authors:** Edna O. Viegas, Arne Kroidl, Patricia J. Munseri, Marco Missanga, Charlotta Nilsson, Nelson Tembe, Asli Bauer, Agricola Joachim, Sarah Joseph, Philipp Mann, Christof Geldmacher, Sue Fleck, Wolfgang Stöhr, Gabriella Scarlatti, Said Aboud, Muhammad Bakari, Leonard Maboko, Michael Hoelscher, Britta Wahren, Merlin L. Robb, Jonathan Weber, Sheena McCormack, Gunnel Biberfeld, Ilesh V. Jani, Eric Sandström, Eligius Lyamuya, TaMoVac study group

**Affiliations:** 1 Instituto Nacional de Saúde (INS), Maputo, Mozambique; 2 Division of Infectious Diseases and Tropical Medicine, University Hospital, LMU Munich, Germany; 3 German Centre for Infection Research (DZIF), partner site Munich, Munich, Germany; 4 Muhimbili University of Health and Allied Sciences (MUHAS), Department of Internal Medicine, Dar es Salaam, Tanzania; 5 National Institute for Medical Research, Mbeya Medical Research Center (NIMR-MMRC), Mbeya, Tanzania; 6 Karolinska Institutet, Department of Laboratory Medicine, Huddinge, Sweden; 7 Public Health Agency of Sweden (PHAS), Department of Microbiology, Solna, Sweden; 8 Muhimbili University of Health and Allied Sciences (MUHAS), Department of Microbiology and Immunology, Dar es Salaam, Tanzania; 9 MRC Clinical Trials Unit at UCL, London, United Kingdom; 10 Istituto di Ricovero e Cura a Carattere Scientifico (IRCCS), San Raffaele Scientific Institute, Viral Evolution and Transmission Unit, Milan, Italy; 11 Karolinska Institutet, Department of Microbiology, Tumor and Cell Biology, Stockholm, Sweden; 12 The US Military HIV Research Program, the Henry M Jackson Foundation for the Advancement of Military Medicine and Walter Reed Army Institute of Research, Silver Spring, MD, United States; 13 Imperial College London, London, United Kingdom; 14 Karolinska Institutet, Department of Public Health Sciences, Stockholm, Sweden; 15 Karolinska Institutet at Södersjukhuset, Venhälsan, Stockholm, Sweden; Rush University, UNITED STATES

## Abstract

**Background:**

We evaluated the safety and immunogenicity of (i) an intradermal HIV-DNA regimen given with/without intradermal electroporation (EP) as prime and (ii) the impact of boosting with modified vaccinia virus Ankara (HIV-MVA) administered with or without subtype C CN54rgp140 envelope protein adjuvanted with Glucopyranosyl Lipid A (GLA-AF) in volunteers from Tanzania and Mozambique.

**Methods:**

Healthy HIV-uninfected adults (N = 191) were randomized twice; first to one of three HIV-DNA intradermal priming regimens by needle-free ZetaJet device at weeks 0, 4 and 12 (Group I: 2x0.1mL [3mg/mL], Group II: 2x0.1mL [3mg/mL] plus EP, Group III: 1x0.1mL [6mg/mL] plus EP). Second the same volunteers received 10^8^ pfu HIV-MVA twice, alone or combined with CN54rgp140/GLA-AF, intramuscularly by syringe, 16 weeks apart. Additionally, 20 volunteers received saline placebo.

**Results:**

Vaccinations and electroporation did not raise safety concerns. After the last vaccination, the overall IFN-γ ELISpot response rate to either Gag or Env was 97%. Intradermal electroporation significantly increased ELISpot response rates to HIV-DNA-specific Gag (66% group I vs. 86% group II, p = 0.026), but not to the HIV-MVA vaccine-specific Gag or Env peptide pools nor the magnitude of responses. Co-administration of rgp140/GLA-AF with HIV-MVA did not impact the frequency of binding antibody responses against subtype B gp160, C gp140 or E gp120 antigens (95%, 99%, 79%, respectively), but significantly enhanced the magnitude against subtype B gp160 (2700 versus 300, p<0.001) and subtype C gp140 (24300 versus 2700, p<0.001) Env protein. At relatively low titers, neutralizing antibody responses using the TZM-bl assay were more frequent in vaccinees given adjuvanted protein boost.

**Conclusion:**

Intradermal electroporation increased DNA-induced Gag response rates but did not show an impact on Env-specific responses nor on the magnitude of responses. Co-administration of HIV-MVA with rgp140/GLA-AF significantly enhanced antibody responses.

## Introduction

Although there has been a decline in the number of new human immunodeficiency virus (HIV) infections over the years, millions of people continue to be exposed and infected [1].

More than 200 HIV vaccine phase I and II, and 6 efficacy trials have been conducted [2, 3]. The RV144 Thai trial is the only trial to show a moderate protective effect using a canary poxvirus vector (ALVAC-HIV vcp1521)-based prime followed by alum-adjuvanted protein (AIDSVAX-gp120 B/E) boost vaccination strategy [4]. In the analysis of immune correlates of risk of HIV infection, antibodies against the V1/V2 region of HIV-1 envelope (Env) were inversely correlated with the rate of HIV-infection, while the presence of IgA Env-binding antibodies was associated with a lack of protection. Furthermore, antibody-dependent cellular cytotoxicity (ADCC)-mediating antibodies correlated with a reduced risk of HIV-infection in vaccinees with low IgA Env binding antibody titers [5].

DNA-based vaccines carrying HIV-1 genes have been shown to be safe and to induce potent cellular immune responses when used in combination with genetically modified vector-based vaccines containing HIV-1 inserts [6–13]. Over the past 11 years, the safety and immunogenicity of a multigene multiclade HIV-1 DNA vaccine candidate (HIV-DNA), boosted with heterologous HIV-1 modified vaccinia virus Ankara (MVA)-Chiang Mai double recombinant (CMDR) vaccine (HIV-MVA) have been assessed in phase I/II HIV vaccine trials [11–16]. Different doses and modes of delivering HIV-DNA vaccine were evaluated in these trials. Potent and durable immune responses were induced after three HIV-DNA and two HIV-MVA immunizations [11, 17, 18]. Furthermore, HIV-DNA vaccine administered intradermally (ID) in a simplified injection regimen (one or two injections), using a needle-free jet device (Biojector) in a total dose of 600μg efficiently primed cellular immune responses after HIV-MVA vaccination [12].

Electroporation (EP) has been used both in pre-clinical and clinical studies to augment the plasmid DNA immunogenicity [19, 20]. EP increases the transfection efficiency into antigen-presenting cells (APCs) by creating transient pores in the cell membranes increasing the uptake of DNA. Clinical studies have suggested that intramuscular (IM) EP can enhance the immunogenicity of DNA vaccines [21, 22], but other routes of administration should be considered to reduce discomfort.

Adjuvanted HIV-1 Env protein-based vaccines have been shown to stimulate humoral immune responses including binding and neutralizing antibodies [23]. Vax003 and Vax004 phase III trials, which evaluated vaccine regimens containing recombinant gp120 (B/E) and gp120 (B/B), respectively, failed to confer protection [24, 25]. In contrast, the RV144 trial which used a vectored-based prime vaccine and recombinant gp120 (B/E) protein boosts succeeded in conferring a moderate protection against HIV infection [4].

The present study builds on previous data from trials in Sweden, Tanzania and Mozambique [13, 14, 26], and aimed to determine the optimal prime boost regimen to take forward to efficacy testing by evaluating whether (i) ID EP with the Derma Vax (Cellectis) device would boost responses to 600μg HIV-DNA, (ii) combining the DNA plasmids in a single injection would compromise responses, and (iii) combining GLA-AF adjuvanted CN54rg140 protein with the HIV-MVA would improve the magnitude or functionality of humoral responses. We chose to address these questions in a single trial using a factorial design.

## Materials and methods

### Ethics and regulatory statement

This study received ethical clearance from the institutional review boards of the Muhimbili University of Health and Allied Sciences, the Mbeya Medical Research Ethics Committee, and the National Institute for Medical Research, in Tanzania; the National Health Bioethics Committee, in Mozambique; the Regional Ethics Committee in Stockholm, Sweden and the Ethics Committee of the Ludwig Maximilian University in Munich, Germany. The Tanzania Food and Drugs Authority (TFDA), in Tanzania and the Pharmaceutical Department, Ministry of Health, in Mozambique, granted regulatory approvals. Study investigators followed the principles of the International Council of Harmonization and Good Clinical Practice guidelines (ICH-GCP). Written informed consent was obtained prior to any study activities. Participants were required to have passed an assessment of understanding prior to any screening procedures. The trial is registered at the US National Institutes of Health (NCT01697007).

### Study design and population

This phase II randomized, placebo-controlled, double–blinded factorial trial was conducted at three different locations: a) the Muhimbili University of Health and Allied Sciences (MUHAS) in Dar es Salaam, and b) the National Institute for Medical Research-Mbeya Medical Research Center (NIMR-MMRC) in Mbeya, Tanzania, and c) the Polana Caniço Health Research and Training Center-Instituto Nacional de Saúde (CISPOC-INS) in Maputo, Mozambique. Study participants were recruited from the Police and Prisons forces, youth friendly clinics and general population in Dar es Salaam; from the general population in Mbeya; and from a cohort of young adults in Maputo. Healthy subjects, aged 18 to 40 years, who were at low risk for acquiring an HIV infection, and neither pregnant nor planning to conceive a child for the duration of the trial, were eligible to participate. Effective birth control practice was required throughout the study, for both male and female volunteers. At screening, subjects diagnosed with HIV, syphilis and hepatitis B virus infection as well as pregnant and breastfeeding women were excluded from study participation. Exclusion criteria also included findings in the electrocardiogram (ECG) suggestive of cardiac disease or that could interfere in the interpretation of peri/myocarditis [27], clinically relevant medical conditions, allergy to vaccines, and subjects taking disallowed medication or other drugs.

### Randomization and vaccinations

Participants were randomized twice in a factorial design; first to one of three HIV-DNA priming regimens as summarized in Table 1. Within each group, subjects were also randomized to receive vaccine or placebo in a ratio of 10:1. Vaccinations were administered ID using the needle-free Zetajet device (Inovio Pharmaceuticals, Plymouth Meeting, PA, USA), in the deltoid region, at weeks 0, 4 and 12. For participants receiving 2 injections per immunization, separate deltoids were injected. ID EP was applied using the Derma Vax device (donated by Cellectis AS, Romainville, France) at the injection site, after administration of the HIV-DNA/placebo. Second, participants receiving the active product were randomized to receive two boosts with HIV-MVA/placebo with or without subtype C rgp140/GLA-AF or placebo IM at a ratio of 1:1. The vaccines were administered as separate injections into opposing deltoid muscles and scheduled to be given at weeks 24 and 40. Not all boost vaccinations were given on schedule due to delays with vaccine supply, nonetheless, the 16 week interval between the two boosts was kept. Subjects were followed for 12 weeks after the last injection. Randomization was based on a computer-generated, sequentially numbered list (random permuted blocks of varying size), stratified by centre and gender, and sent in sequentially numbered sealed envelopes to the site pharmacists who randomized the participant and prepared the vaccines. If participants prematurely terminated their vaccination schedule whilst recruitment was ongoing, additional participants were randomized to meet the target sample size.

**Table 1:** Randomized study groups, doses, routes and time-points of different HIV-DNA priming (1^st^ randomization) and HIV-MVA with or without CN54rgp140/GLA-AF boosting (2^nd^ randomization) vaccinations.

		First randomization	Second randomization
Group (n = target)		HIV-DNA prime (Weeks 0, 4, 12)	HIV-MVA+/- CN54rgp140/GLA-AF boost (Weeks 24, 40)
**I**	**Vaccine (n = 60)**	**2 inj.** x 0.1 mL total 600 μg (**3mg/mL**) by **ID Zetajet**	**A.** 1 mL HIV-MVA 10^8^ pfu **IM plus** 0.4 mL [100 μg] CN54rgp140/GLA-AF [5μg] **IM (n = 30)**
			**B.** 1mL HIV-MVA 10^8^ pfu **IM (n = 30)**
	**Placebo (n = 6)**	**2 inj.** of 0.1mL saline by **ID Zetajet**	**A.** 1 mL saline **IM plus** 0.4 mL saline **IM (n = 3)**
			**B.** 1 mL saline **IM (n = 3)**
**II**	**Vaccine (n = 60)**	**2 inj.** x 0.1 mL total 600 μg (**3mg/mL**) by **ID Zetajet + Derma Vax EP**	**A.** 1 mL HIV-MVA 10^8^ pfu **IM plus** 0.4 mL [100 μg] CN54rgp140/GLA-AF [5μg] **IM (n = 30)**
			**B.** 1mL HIV-MVA 10^8^ pfu **IM (n = 30)**
	**Placebo (n = 6)**	**2 inj.** of 0.1mL saline by **ID Zetajet + Derma Vax EP**	**A.** 1 mL saline **IM plus** 0.4 mL saline **IM (n = 3)**
			**B.** 1 mL saline **IM (n = 3)**
**III**	**Vaccine (n = 60)**	**1 inj.** x 0.1 mL total 600 μg (**6mg/mL**) by **ID Zetajet + Derma Vax EP**	**A.** 1 mL HIV-MVA 10^8^ pfu IM **plus** 0.4 mL [100 μg] CN54rgp140/GLA-AF [5μg] **IM (n = 30)**
			**B.** 1mL HIV-MVA 10^8^ pfu **IM (n = 30)**
	**Placebo (n = 6)**	1 inj. of 0.1mL saline by **ID Zetajet + Derma Vax EP**	**A.** 1 mL saline **IM plus** 0.4 mL saline **IM (n = 3)**
			**B.** 1 mL saline **IM (n = 3)**

### Vaccines

Details on the vaccines, composition and derivation are provided in the S1 Table. In brief, the HIV-DNA is composed of seven plasmids delivered in two separate pools (pool 1: Env gp160 A/B/C and Rev B; pool 2: Gag p37 A/ B and RTmut B) [16, 28]. The vaccine was formulated in physiological saline at a concentration of 3mg/mL for groups I and II, or pooled at 6mg/mL for group III. The HIV-MVA is a multigenic live recombinant replication-deficient poxvirus vector-based vaccine that contains HIV-1 gp150 E, and Gag and Pol A [29]. The CN54rgp140 is a trimeric recombinant subtype C HIV-1 gp140 Env glycoprotein. [30, 31]. GLA-AF is an adjuvant containing an aqueous formulation of glucopyranosyl lipid A, which is a synthetic monophosphoryl lipid A (MPL)-like molecule [32], a ligand for toll-like receptor 4 (TLR4) and potent stimulator of the antigen presenting cells.

The vaccines were thawed at the pharmacy, dispensed into syringes and labelled with the study code. They were kept under refrigeration (+2-8ºC) and administered within 4 hours of being dispensed. CN54rgp140 and GLA-AF were mixed prior to IM administration. Sterile commercially available normal saline for humans was used as the placebo.

The study team and the participants were blinded to vaccine or placebo administration but not to the treatment arms.

### Safety assessment

Laboratory safety assessments (complete blood count, glucose, ALT, creatinine and direct bilirubin) were performed two and four weeks after each vaccination and at the last follow-up visit. Local and systemic solicited adverse events were collected 30 minutes after injections, and on diary cards the same evening and for seven days following each immunization. Local events included pain, itching, warmth, swelling, erythema and induration. Systemic events included fever (axillar temperature >37.5°C), malaise, chills, arthralgia, myalgia, headache, nausea and vomiting. A 12-lead ECG was performed at screening and whenever a suspicion of cardiac impairment was present.

Non-solicited adverse events (AEs) and the use of concomitant medications were collected from the time of the 1^st^ vaccine injection up to the last study visit and graded for severity according to the DAIDS Adverse Event Grading Table, version 1.0 (Division of AIDS, National Institutes of Health) [33], except for neutropenia for which the cut-off for grade 1 was adjusted to a lower local reference ranges (1100 cells/μL) [34]. AEs were classified for causality as not related, probably not related, possibly related, probably related and definitely related to the investigational products. Events were categorized using MedDRA version 19.1 System Organ Class (SOC) terminology.

Urinalysis, urine pregnancy and HIV tests were performed at screening and prior to each vaccine administration. Female volunteers with a positive pregnancy test were ineligible for further vaccinations and were followed until delivery. HIV infected individuals were stopped from further vaccinations but follow-up continued until the last study visit. Monitoring for HIV-1 infection was performed using a sequential algorithm of a first enzyme-linked immunosorbent assay (ELISA) screening which was confirmed when reactive by a second ELISA, immunoblotting and/or quantitative HIV RNA assessments as previously described [12]. Only cases with detectable HIV RNA were considered HIV infected, otherwise interpreted as reactive due to vaccine-induced responses. HIV status reports to the study clinics were termed HIV infected or not infected to maintain blinding. All pregnant and HIV-infected subjects were referred for clinical follow-up at a health facility of the national health system.

### Immunogenicity assessment

IFN-γ ELISpot assays were performed on freshly isolated PBMC using the h-IFN-γ ELISpot PLUS kit in a two-step detection system (Mabtech, Nacka, Sweden) as previously described [11]. Vaccine-induced T cell responses were determined using HIV-1-specific peptide pools matching HIV-DNA Gag_p37_ (Gag Smi) encoding subtype B p17 and subtype A and B p24, HIV-MVA subtype A Gag_p55_, including the p15 region (Gag CMDR) [35], and HIV-MVA subtype E Env (Env CMDR) at a purity of >80% (JPT, Peptide Technologies, Berlin, Germany). Results were expressed as spot forming cells (SFC)/10^6^ PBMC. ELISpot responses were considered positive if the number of SFC/10^6^ PBMC was >4 times the background (medium only), >4 times the baseline (pre-immunization) value, and >55 SFC/10^6^ PBMC. Data were excluded from analyses if the background responses in medium wells or pre-immunization values exceeded 60 SFC/10^6^ PBMC.

Analyses of antibody-mediated immune responses were performed on serum or plasma samples. Binding IgG antibody responses to subtype B IIIB gp160 protein (Advanced Biotechnologies Inc., Columbia, MD), subtype C CN54 gp140 recombinant protein (homologous to the protein immunogen) and subtype E 93TH975 gp120 recombinant protein (NIH AIDS Research and Reference Reagents program, Division of AIDS, NIAD, Germantown, USA) were measured using three-fold dilution series in ELISA as detailed previously [26]. Data were reported as reciprocal end-point titers.

Neutralization antibody activity was measured using a TZM-bl cell based assay employing pseudoviruses and a luciferase reporter gene readout. The detailed protocol for virus titration and assay is available at EUROPRISE website (www.europrise.org/neutnet_sops.html). The pseudoviruses used were SF162 subtype B, 93MW965 subtype C, GC015.EC12 subtype C, Th023.06 CRF01_AE and CM235 CRF01_AE. Briefly, 2-fold dilutions starting with 1:20 of each serum sample were incubated with viral supernatant (200 TCID50) for 1 hour. Thereafter, 10^4^ TZM-bl cells were added and plates were incubated for 48 hours, when luciferase activity was measured. Neutralization titers were defined as the samples dilution at which relative luminescence units (RLU) were reduced by 50% in the test sample wells compared to virus control wells, after subtraction of background RLU in control wells with only cells. Testing against VSV was used to exclude unspecific reactions.

### Study endpoints

The primary safety endpoints were defined as any grade 3 or above local or systemic clinical or laboratory solicited AE or any grade of AE that resulted in a clinical decision to discontinue immunizations. Secondary safety endpoints were defined as any grade of AE in a participant that had received at least one immunization.

The primary immunogenicity endpoint for the first randomization was the presence of IFN-γ ELISpot responses to Gag or Env two weeks after the final vaccination; and for the second randomization, (a) the magnitude of binding antibody responses to subtype C Env and (b) the presence and magnitude of neutralizing antibody responses four weeks after the final vaccination.

Secondary immunogenicity endpoints included the magnitude of IFN-γ ELISpot responses, the presence of antibody responses to subtype C gp140, subtype B gp160 and CRF01_AE gp120, as well as the magnitude of antibody responses to subtype B gp 160 and CRF01_AE gp120.

### Statistical analysis

For the first randomization an absolute difference of 30% (50% versus 80%) in the proportion of cellular responders was considered clinically relevant and a sample size of 60 per group was required for a power of 90% and significance level of 2.5% to adjust for multiple comparisons. For the second randomization we were interested in a 75% increase in the magnitude of humoral immune responses (corresponding to a difference of approximately .243 on a log10 scale, assuming a standard deviation of 0.5) in line with the results seen in RV144 and a sample size of 90 per group provided 90% power at a 5% significance level.

Safety data were transcribed from source documents to case report forms and double entered in a SQL Server 2008 Express edition database (Microsoft, Redmond, WA). Immunological data were entered into Microsoft Office Excel 2007 (Microsoft, Redmond, WA). Data were exported and analyzed in Stata 14 (StataCorp. 2015. Stata: Release 14. Statistical Software. College Station, TX: StataCorp LP).

Safety data analysis was performed using a modified intent-to-treat (mITT) approach that included all-randomized participants who received at least one HIV-DNA (first randomization and overall). For comparison of the second randomisation, events were limited to those occurring after the first boost vaccination. The proportion of participants who ever experienced a particular type of adverse event was compared between the experimental vaccination groups. Solicited and non-solicited events were summarized according to the maximum grade of severity as mild, moderate or severe.

The immunological analysis was limited to valid laboratory tests in participants who completed all the scheduled immunizations. For the various immunogenicity endpoints, we compared the proportion of responders and the magnitude in responders between HIV-DNA priming groups and between the HIV-MVA with or without CN54rgp140/GLA-AF boosting groups.

For both safety and immunogenicity outcomes, comparisons of proportions were made using chi-square test or Fisher’s exact test where appropriate. 95% confidence interval (CI, Agresti-Caffo method [36]) of the absolute difference between groups was reported for immune responses. The magnitude of responses was described using median and interquartile range (IQR), and comparisons between the randomization groups were made by Wilcoxon rank-sum test. Logistic regression was used to analyse the association between immune responses and possible predictors. All tests were performed without adjustments for multiple comparisons.

## Results

### Demographics, recruitment and inclusion

Between November 2012 and November 2013, 502 volunteers were screened, and 211 (42%) were enrolled; 249 (49.6%) did not meet the eligibility criteria; and 42 (14%) were eligible but not enrolled due to declining participation, not returning to the study clinic or because the study was already fully enrolled. The site affiliations and baseline characteristics of the 211 participants enrolled are shown in S2 Table. In brief, 46% were female, the median age was 22 years, the median body mass index (BMI) was 22 kg/m^2^, and 32% had scars compatible with previous vaccinia vaccination. Baseline characteristics were balanced across the randomization group.

### Withdrawals/Termination from vaccination

**Fig 1:**
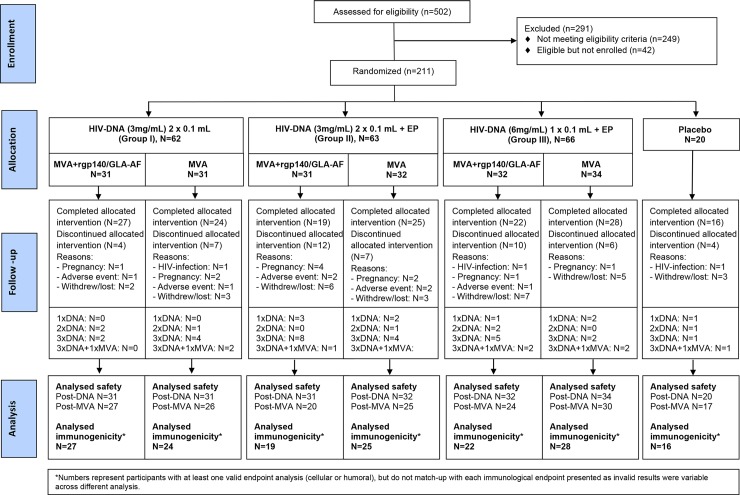
The number of individuals screened, randomized, allocated and withdrawn from the trial and the number of samples analyzed.

The study flow, retention to the vaccination schedule and contribution to analysis datasets are shown in Fig 1. Overall, 50 of 211 (23.7%) participants did not complete the vaccination schedule, with 26/50 (52%) dropping out after the last HIV-DNA vaccination, mainly due to a substantial delay in the first HIV-MVA boost whilst a stability concern was addressed. The first boost was delivered at a median of 43 weeks (range 23–58), i.e. 31 weeks (range 11–47) after the third HIV-DNA instead of the intended 12-week gap, and this was similar across the first and second randomisation groups. The 16-week interval between the two boost vaccinations was maintained. Other reasons for early termination included pregnancy in 11/50 (22%), adverse events that were not considered to be related to vaccine in 7/50 (14%) and HIV infection in 3/50 (6%) cases. Overall, out of 191 vaccine recipients, 177 (92.7%) completed all three prime vaccinations but only 152 continued with either HIV-MVA alone or HIV-MVA/rgp140/GLA-AF, and 145 (75.9%) completed the two boost immunizations. Sixteen (80%) of placebo recipients completed their injection regimen. Five vaccine and one placebo recipient relocated after the completed vaccination schedule and did not attend the last study visit.

### Primary safety endpoint

Forty participants experienced a primary safety endpoint: 35/191 (18%) in an experimental vaccine group and 5/20 (25%) placebo recipients. There were neither differences between vaccine groups of the first randomisation (p = 0.42) nor between vaccine groups of the second randomisation (p = 0.48). The majority of primary safety events were solicited local, systemic or laboratory events but 10 participants discontinued further immunisations following an adverse event. These events were 3 HIV infections, 2 hyperthyroidisms, 1 each of pulmonary tuberculosis, cannabis induced psychosis, fibroadenoma of the breasts, hypertension and iron deficiency anaemia and none were considered to be related to the study vaccines.

For solicited adverse events, of the 211 participants who received at least one HIV-DNA or placebo immunization, 124 (59%) reported a local and/or 133 (63%) a systemic solicited AE starting within one week of vaccination. All events were mild or moderate except for five (one in Group I, three in Group III, one placebo) which were transient grade 3 body temperature elevation. The most common solicited events were headache (47%), local pain (44%), itching (39%), arthralgia (23%), elevated body temperature (19%), induration (18%), myalgia (18%), swelling (16%), nausea (15%), and chills/rigor (11%). The proportion of participants with local or systemic solicited events was similar across all 3 HIV-DNA groups (S1 Fig).

On a visual analogue scale (0 = none, 10 = worst) the median score for discomfort or pain in 142 participants who received one or more ID EP was 2 (range 1–8), 23% scored 4–6, and 2% scored 7–8. The median score 30 minutes after vaccination was 0 (range 0–3).

Of 169 participants who received at least one HIV-MVA alone, HIV-MVA/rgp140/GLA-AF or placebo boosting, 91 (54%) reported any local and 80 (47%) any systemic solicited AE. All events were mild or moderate with the exception of three participants, one of whom reported severe pain, itching and warmth as well as severe chills/rigor, arthralgia, myalgia, headache and nausea following the second HIV-MVA immunization. The other two severe events were transient elevated temperature (HIV-MVA/rgp140/GLA-AF) and nausea (HIV-MVA alone). The most frequently reported events were local pain (52%), headache (25%), itching (21%), arthralgia (19%) or myalgia (17%), warmth (16%), elevated temperature (13%) and nausea (11%). The proportion of participants reporting local or systemic solicited events was similar in both boost groups (S1 Fig).

Overall, 511 non-solicited adverse events were reported by 159 (75.4%) participants who received at least one vaccination and details are provided in S3 Table. Only six were considered severe in grade, and the five serious adverse events were all hospitalization during the HIV-DNA priming period for events that were not considered to be related to the vaccinations. There were no significant differences between the vaccine and placebo groups overall, or between the vaccine groups for the first and second randomisations, although the higher-grade events were only observed in the vaccine groups. Two of these started within 28 days of immunisation (hypochromic microcytic anaemia and malaria). There were four (1.9%) HIV infections, two in vaccine recipients after the first (Group III) and third (Group I) HIV-DNA prime, and two in placebo recipients.

Laboratory safety data were available for 209. Overall, 137 (65.6%) participants had at least one laboratory event at any time during the entire trial, of whom 26 (12%) had a severe or worse event, 17 (8.1%) experiencing these within 28 days of vaccination. The majority of the higher-grade abnormalities were neutropenia. Transient bilirubinaemia occurred in two participants, both within 28 days of receiving the combination of MVA and adjuvanted protein.

### Immunological outcomes

Data from IFN-γ ELISpot testing in 151 vaccinees were available after completion of three vaccinations. The frequency of Gag-specific responses observed after stimulation with Gag Smi and/or Gag CMDR peptide pools, i.e. to any Gag peptide pool, two weeks after the third HIV-DNA immunization was 11/50 (22%), 11/51 (22%) and 10/50 (20%) for group I, II and III, respectively. An even lower frequency of response was seen to Env, 2/50 (4%), 3/51 (6%) and 2/50 (4%) in groups I, II and II respectively (data not shown).

Results from IFN-γ ELISpot testing against Gag CMDR and Env CMDR peptide pools specific for the HIV-MVA vaccine were available from 126 vaccinees who had completed all vaccinations. There were 121 vaccinees (96%) with ELISpot responses to either Gag CMDR and/or Env CMDR peptides with 90% responding to Gag and 90% responding to Env peptide pool stimulation two weeks after the final vaccination. There was no statistical difference in the overall response rates between the three HIV-DNA priming immunization groups being 98%, 100% and 93%, respectively, nor was there a difference in ELISpot response rates by the second randomization; i.e. between receiving HIV-MVA+rgp140/GLA-AF and HIV-MVA alone, with response rates of 97% and 97%, respectively (Table 2).

**Table 2:** Frequency of ELISpot Responses Two Weeks after the 2^nd^ MVA Alone or MVA Plus rgp140/GLA-AF Vaccination by First (Group I: 2x 0.1 mL ID [3mg/mL], Group II: 2x 0.1 mL ID + Electroporation [3mg/mL], Group III: 1x 0.1 mL ID + Electroporation [6mg/mL]) and Second (MVA Plus rgp140/GLA-AF or MVA Alone) Randomizations.

	First Randomization							Second Randomization		
Peptide Pool	Group I	Group II	Group III	Overall P-value	Group II vs I*	Group III vs. I*	Group III vs. II*	MVA + Protein	MVA	P-value
**Gag Smi**	25/38 (66)	32/36 (89)	29/36 (81)	0.07	23 (4 to 40) **p = 0.026**	15 (-6 to 34) p = 0.19	-8 (-25 to 9) 0.51	34/47 (72)	52/63 (82)	0.20
**Gag CMDR**	41/45 (91)	37/39 (95)	36/42 (86)	0.63	4 (-9 to 15) p = 0.68	-5 (-19 to 9) p = 0.51	-9 (-22 to 5) 0.27	53/59 (90)	61/67 (91)	0.82
**Env CMDR**	39/45 (87)	37/39 (95)	37/42 (88)	0.76	8 (-5 to 21) 0.28	1 (-13 to 16) p = 1.0	-7 (-19 to 7) 0.43	51/59 (86)	62/67 (93)	0.26
**Any Gag**	41/45 (91)	37/39 (95)	37/42 (88)	0.84	4 (-9 to 15) p = 0.68	-3 (-16 to 10) p = 0.73	-7 (-19 to 7) 0.43	53/59 (90)	62/67 (92)	0.59
**Gag and/or Env**	44/45 (98)	39/39 (100)	39/42 (93)	0.21	2 (-6 to 9) p = 1.00	-5 (-15 to 5) p = 0.35	-7 (-16 to 3) 0.24	57/59 (97)	65/67 (97)	1.0

Values are provided in numbers of responders (n)/numbers of participants with valid assays (N) and percentage (%)

* Absolute difference (95% CI), p-value. Overall response rate to either Gag Smi, Gag CMDR or Env peptide pools: 122/126 (97%); Gag Smi; HIV-DNA Gag_p37_ (subtype B p17 and subtype A and B p24)-specific peptide pool, Gag CMDR and Env CMDR; peptide pools specific for the subtype A Gag_p55_ and subtype E Env inserts in HIV-MVA (MVA CMDR). All peptide pools consisted of 15- to 18-mers with 11-aa overlap.

Data for IFN-γ ELISpot responses to the Gag Smi peptide pool specific for the HIV-DNA vaccine were available from 110 vaccinees who had completed all vaccinations. Two weeks after the last vaccination, Gag Smi-specific responses were frequent in all three DNA priming immunization groups. There was one vaccinee with Gag Smi-specific response that did not react with the Gag CMDR or Env CMDR peptide pools. Thus, the overall response rate to Gag Smi, Gag CMDR and/or Env CMDR was 122/126 (97%). The Gag Smi-specific ELISpot response rate was significantly higher in vaccinees given EP in combination with the HIV-DNA vaccine in separate deltoids (group II, 32/35, 86%) than in vaccinees receiving HIV-DNA without EP (group I, 25/38, 66%), p = 0.026 (Table 2).

Due to the delay between the last HIV-DNA prime and first HIV-MVA boost we examined whether this had any impact on the IFN-γ ELISpot responses after the final vaccination and found a significantly decreased probability of Gag Smi-specific responses with longer intervals (OR 0.88 per additional week, 95%CI 0.80–0.97, p = 0.011) and a trend for decreased Gag CMDR-specific responses (OR 0.90, 95%CI 0.80–1.00, p = 0.060); all analyses were adjusted for randomisation arm, sex, age and site.

**Fig 2:**
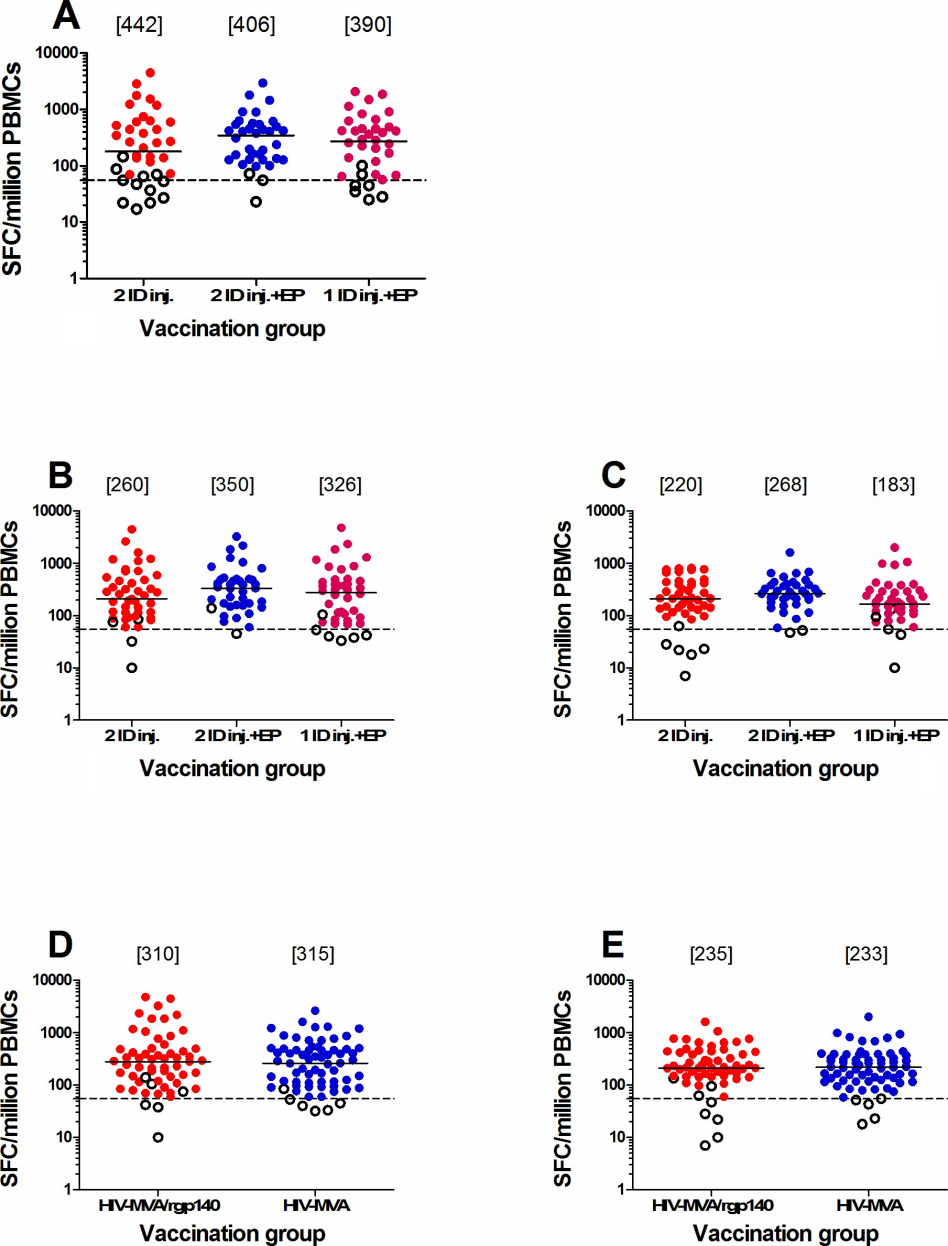
IFN-γ ELISpot responses. (A) the DNA vaccine-specific Gag Smi peptide pool and, (B) the HIV-MVA vaccine-specific Gag CMDR, (C) Env CMDR peptide pools by the first randomization, to (D) Gag CMDR and (E) Env CMDR peptide pool stimulation by the second randomization in samples collected two weeks after the final vaccination in vaccine recipients only. Responders and non-responders are shown by filled and open circles, respectively. Median values in responders are given in brackets.

Fig 2 shows the magnitude of the IFN-γ ELISpot responses to Gag and Env peptide pools two weeks after the final vaccination. There was no significant difference in magnitude of the ELISpot responses to the Gag Smi peptide pool between the three DNA priming immunization groups. The median responses in responders to Gag Smi were 442 SFC/million PBMC (IQR 208–752), 406 SFC/million PBMC (IQR 158–555) and 390 SFC/million PBMC (IQR 205–625), respectively (p = 0.63). In addition, there was no significant difference in magnitude of the ELISpot responses to Gag CMDR or Env CMDR peptide pools between the three DNA priming immunization groups. The median responses to Gag CMDR were 260 SFC/million PBMC (IQR 117–540), 350 SFC/million PBMC (IQR 172–505) and 326 SFC/million PBMC (IQR 118–501), respectively (p = 0.62). The median responses to Env were 220 SFC/million PBMC (IQR 145–440), 268 SFC/million PBMC (IQR 210–390) and 183 SFC/million PBMC (IQR 135–305), respectively (p = 0.15) (Fig 2).

Concurrent administration of CN54rgp140/GLA-AF with MVA had no impact on the magnitude of the ELISpot responses. The median Gag CMDR-specific responses were 310 (IQR 157–505) and 315 (IQR 120–507) SFC/million PBMC, for those receiving CN54rgp140 or not, respectively, and the median Env-specific responses were 235 (IQR 167–440) and 233 (IQR 137–385) SFC/million PBMC, respectively (Fig 2).

One placebo recipient among 11 evaluable had ELISpot reactivity to the Gag Smi, Gag CMDR and Env CMDR peptide pools 2 weeks after the final injection (910, 870 and 395 SFC/million PBMC, respectively), but was not reactive at other time points.

**Fig 3:**
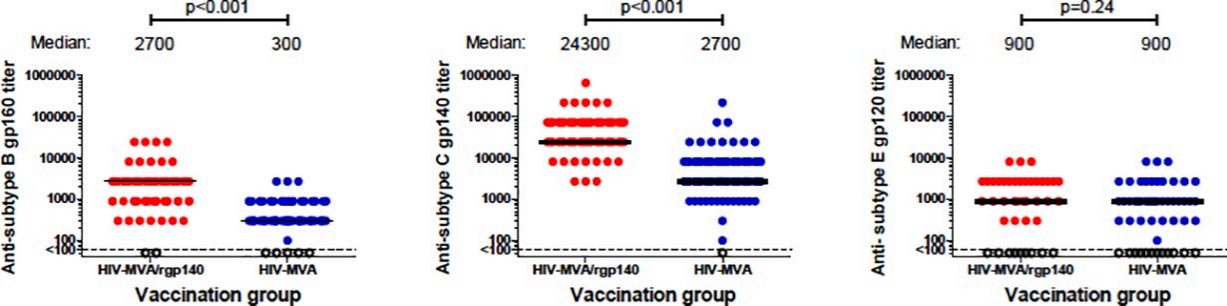
Binding antibody responses. (A) subtype B gp160, (B) subtype C gp140 and (C) subtype E gp120 antigen by the second randomization, in samples collected four weeks after the final vaccination in vaccine recipients only. Responders are shown by filled circles and non-responders are shown by open circles. The Wilcoxon rank-sum test was used for comparisons.

Binding antibody responses measured in serum/plasma four weeks after the final vaccination are shown in Fig 3. Overall, 124/131 (95%) exhibited binding IgG antibodies to subtype B gp160, 135/136 (99%) to subtype C gp140 and 80/101 (79%) to subtype E gp120 (Table 3).

**Table 3:** Frequency of antibody responses as determined by ELISA four weeks after the final vaccination.

	Randomization Group		Comparison
Antigen	10^8^ pfu HIV-MVA plus rgp140/GLA-AF	10^8^ pfu HIV-MVA	(P-value)
**Subtype B gp160**	59/61 (97)	65/70 (93)	0.33
**Subtype C gp140**	62/62 (100)	73/74 (99)	1.00
**Subtype E gp120**	40/49 (82)	40/52 (77)	0.56

Values are provided in numbers of responders (n)/numbers of participants with valid assays (N) and percentage (%).

Concurrent administration of CN54rgp140/GLA-AF with HIV-MVA had no significant influence on the frequency of binding antibody responses to subtype B gp160 and subtype C gp140, with a response rate close to 100%, but addition of the adjuvanted protein significantly increased the magnitude of antibody responses to subtype B gp160 and subtype C gp140, but not to subtype E gp120 (Table 4, Fig 3). Notably, the highest antibody titers were induced against the subtype C gp140 antigen (homologous to the CN54rgp140 vaccine) with median titer of 24300 (IQR 24300–72900) and 2700 (IQR 2700–8100), respectively, in vaccinees receiving HIV-MVA+rgp140/GLA-AF and HIV-MVA only.

**Table 4:** Neutralizing Antibodies (NAb) determined four weeks after the final vaccination using the TZM-bl cell platform by second randomization.

Pseudovirus	Frequency of Response (Number of Positive/Number of Tested, %)		P-value	Magnitude of Response (Median Titer)	
	MVA plus rgp140/GLA-AF	HIV-MVA alone		MVA plus rgp140/GLA-AF	HIV-MVA alone
**Subtype B (SF162)**	4/65 (6)	0/75 (0)	**0.04**	27	N/A
**Subtype C (93MW965)**	38/65 (58)	1/75 (1)	**<0.001**	90	150
**Subtype C (GS015.EC12)**	6/65 (9)	3/75 (4)	0.30	25	40
**CRF01_AE (TH.023.06)**	17/65 (26)	10/75 (13)	0.06	34	25
**CRF01_AE (CM235.EC4)**	0/65 (0)	0/75 (%)	N/A	N/A	N/A

The impact of the delay between the last HIV-DNA prime and first HIV-MVA was minimal, with no difference in frequency or magnitude of binding antibody responses after the final vaccination to subtype B gp160 or subtype C gp140, and only significantly more frequent antibody responses with longer intervals to subtype E gp120 among those boosted with HIV-MVA alone (OR 1.11 per additional week, 95%CI 1.02–1.19, p = 0.011).

Four weeks after the final injection, 2 of 16 placebo recipients exhibited binding antibody responses to gp140 subtype C antigen, both with a titer of 100 (the starting dilution used in the assay), while none showed reactivity to gp160 subtype B (n = 15) or gp120 subtype E (n = 14) antigen.

Neutralizing antibodies (NAb) against 5 different pseudoviruses were measured in a TZM-bl cell based assay four weeks after the final vaccination (Table 4). NAb to subtype B SF162 were rare and only found in four vaccinees, who had all received HIV-MVA combined with rgp140/GLA-AF protein. A significantly higher frequency of NAb responses to subtype C 93MW965 was seen in vaccinees that received HIV-MVA+rgp140/GLA-AF than in those receiving HIV-MVA alone, 38/65 (58%) versus 1/75 (1%), p<0.001. NAbs to subtype C GS015.EC12 were rare, but seen in both randomization groups. NAb responses to CRF01_AE TH.023.06 were seen in both randomization groups; in 17 (26%) of 65 vaccinees receiving rgp140/GLA-AF in combination with HIV-MVA and in 10/75 (13%) of vaccinees receiving HIV-MVA alone, p = 0.06. None of the vaccinees exhibited NAbs to CRF01_AE CM235.EC4. In general, the magnitude of the NAb responses was low. The highest titers were noted against subtype C 93MW965 with a median titer of 90 and 150 in vaccinees receiving HIV-MVA+rgp140/GLA-AF and HIV-MVA alone, respectively.

Four weeks after the final injection, 1 of 15 evaluable placebo recipients exhibited NAb reactivity to subtype C (GS015.EC12) with a titer of 31, but no reactivity to any of the other pseudoviruses was observed.

## Discussion

The principal aim of this phase II vaccine trial was to determine the optimal prime boost regimen to take forward to efficacy testing. Although EP with the Derma Vax device safely enhanced the frequency of responders to Gag peptides following ID injection of HIV-DNA, it did not impact on the magnitude of response or the proportion of Env responders, which was high across all groups. Combining the plasmids in a single injection did not compromise immunogenicity, and was simpler to administer than two injections. The addition of adjuvanted protein appeared safe and significantly improved antibody responses, although neutralizing antibody titres were low.

The vaccine regimen was generally well tolerated and the adverse events leading to discontinuation of the schedule and/or hospitalisation were considered unrelated or unlikely to be related, in accordance with results from our previous trials [11–13, 26, 37]. One participant experienced multiple grade 3 local (pain, itching and warmth) and systemic (chills/rigor, arthralgia, myalgia, headache and nausea) adverse events after the second HIV-MVA alone boost, indicating that severe reactogenicity can occur. Transient and clinically irrelevant low neutrophil counts within 28 days post vaccination were the most commonly observed grade 3 or 4 laboratory events. This has also been observed in our previous trials in Tanzania [11, 12] and might be a normal variation in this African population, as has been described previously [38–40]. ID EP was associated with moderate or severe local pain in 25% of participants which is lower than reported in a previous trial using IM EP in which 51% of participants reported moderate or severe pain [41].

ID EP significantly increased the frequency of IFN-γ ELISpot response to a peptide pool specific for the HIV-DNA vaccine (Gag Smi), but not to the MVA-CMDR vaccine-specific peptide pools (Gag CMDR and Env CMDR). These findings extend those from a phase I trial in Swedish vaccinees receiving the same vaccine regimen and ID HIV-DNA immunization using Zetajet delivery and Derma Vax EP, with overall IFN-γ ELISpot response rates of 95% to both Gag CMDR and Env CMDR two weeks after the first HIV-MVA+/- unadjuvanted Env protein vaccination [13]. After a second HIV-MVA +/- protein immunization, the median IFN-γ ELISpot responses in responders to Gag CMDR and Env Gag CMDR were 319 and 242 SFCs/million PBMC, respectively, similar to what is reported here. Responses to the Gag Smi peptide pool were not explored in that study [13]. Vasan et al reported a 63% ELISpot response rate in vaccinees receiving the ADVAX DNA and IM EP (TriGrid delivery System) after two vaccinations, while none of the eight vaccinees receiving a higher ADVAX dose without EP developed ELISpot responses [22]. A study by Kalams et al evaluated the use of PENNVAX-B DNA vaccine plus IL-12 plasmid with CELLECTRA IM EP and showed that 88.9% of participants developed CD4+ and CD8+ T-cell responses [21]. Recently, in a trial by Ake et al a 57% ELISpot response rate was observed in vaccinees from the US and Africa receiving the PENNVAX-G DNA IM by Biojector 2000 or by CELLECTRA IM EP, with EP followed by MVA-CMDR boost not impacting on the cellular immune response [41]. ID EP using the Derma Vax device was well tolerated in the present trial, nonetheless the marginal gain in immunogenicity to Zetajet HIV-DNA prime followed by HIV-MVA boost may not motivate the additional effort needed for ID EP use.

In our previous trials we have shown that using the Bioject needle-free device to administer 1mg, ID HIV-DNA in five injections induced a stronger cell-mediated immune response following HIV-MVA boosting than 3.8 mg HIV-DNA administered IM [11], and that the number of injections can be reduced to two by combining the HIV-DNA plasmids and decreasing the dose 600 μg [12]. The results from the present trial suggest that further simplification to three doses of single injections administered using the Zetajet device, given without EP is a reasonable priming regimen to take forward to efficacy testing.

A second objective of our trial was to investigate the impact of adjuvanted rgp140/GLA-AF administered concurrently with the HIV-MVA boost compared to HIV-MVA boosting alone. The proportion of participants with binding antibodies was high, 79–99% depending on the subtype antigen used, with no difference seen between the two study arms. The magnitude of the binding antibody responses was significantly higher in the HIV-MVA plus rgp140/GLA-AF arm as compared to HIV-MVA alone against subtype C gp140 (homologous to the CN54rgp140 vaccine) and B gp160, but not against subtype E gp120. The median titres in the combination arm are similar to those we observed in a Tanzanian study population who received two sequential rgp140gp140/GLA-AF boosts after three HIV-DNA and two HIV-MVA vaccinations (23400 and 2700 against subtype C gp140 and subtype B gp160 respectively) [26]. In the present study, the higher frequency of NAb in vaccinees given HIV-MVA in combination with subtype C rgp140/GLA-AF was only significant for subtype C 93MW965, a relatively easy to neutralize tier 1 pseudovirus. When rgp140gp140/GLA-AF boosts were given sequentially after HIV-DNA and HIV-MVA vaccinations, no NAb were observed to SF162 subtype B, GS015 subtype C or CM235 CRF01_AE pseudovirus. Subtype C 93MW965 pesudovirus was not included in the panel [26]. The modest enhancement of Env-specific cellular immune responses observed with sequential boosting was not seen in the present study [26].

A major limitation in our trial was the substantial number of early study terminations because of the delay in release of the HIV-MVA vaccine. The targeted vaccine intervals between the last DNA-priming and the first HIV-MVA boosting were delayed by a median of 19 weeks and led to heterogeneity of longer and shorter intervals in participants receiving the booster vaccinations after completion of the DNA-priming. However, as delays were evenly distributed across groups we do not think that this impacted the overall study outcome, but as we found a lower probability of IFN-γ ELISpot Gag Smi-specific responses with the longer interval, this may have led us to underestimate the proportion of responders. IFN-γ ELISpot reactivity pre-immunization or in medium wells reduced the availability of valid ELISpot data for analysis.

In summary, the results from this innovative factorial Phase II trial suggest that we can safely simplify the prime boost regimen to a single HIV-DNA ID injection administered with the Zetajet device on three occasions, followed by two combination boosts with HIV-MVA/adjuvanted rgp140 without compromising the immune responses.

## Supporting information

S1 CONSORT Checklist (PDF)

S1 Table Vaccines composition. (DOCX)

S2 Table Baseline characteristics for participants included in the modified intent-to-treat analysis (mITT), overall and by randomization group. (DOCX)

S3 Table Non-solicited adverse events and laboratory adverse events indicated for the total population, and vaccine and placebo recipients over the entire study period. P-values are given for the comparisons of events between vaccine groups (chi-square/Fisher’s exact test).
(DOCX)

S1 Fig Solicited local and systemic adverse events. (A) After HIV-DNA priming (Group I: 2x 0.1 mL ID [3mg/mL], Group II: 2x 0.1 mL ID + electroporation [3mg/mL], Group III: 1x 0.1 mL ID + electroporation [6mg/mL]), and (B) after HIV-MVA boost alone or HIV-MVA plus rgp140/GLA-AF. P-values are given for the comparisons of any event between experimental vaccine groups (chi-square/Fisher’s exact test).
(TIF)

S1 File Trial protocol. (PDF)

## References

[1] UNAIDS JUN-PoHA. AIDS by the numbers Geneva, Switzerland: 2017.

[2] Day TA, Kublin JG. Lessons learned from HIV vaccine clinical efficacy trials. Curr HIV Res. 2013;11(6):441–9. ; PubMed Central PMCID: PMCPMC4000156. 24033299 10.2174/1570162x113116660051 PMC4000156

[3] O'Connell RJ, Kim JH, Corey L, Michael NL. Human immunodeficiency virus vaccine trials. Cold Spring Harb Perspect Med. 2012;2(12):a007351 10.1101/cshperspect.a007351 ; PubMed Central PMCID: PMCPMC3543076. 23209178 10.1101/cshperspect.a007351 PMC3543076

[4] Rerks-Ngarm S, Pitisuttithum P, Nitayaphan S, Kaewkungwal J, Chiu J, Paris R, et al Vaccination with ALVAC and AIDSVAX to prevent HIV-1 infection in Thailand. N Engl J Med. 2009;361(23):2209–20. Epub 2009/10/22. NEJMoa0908492 [pii] 10.1056/NEJMoa0908492 . 19843557 10.1056/NEJMoa0908492

[5] Haynes BF, Gilbert PB, McElrath MJ, Zolla-Pazner S, Tomaras GD, Alam SM, et al Immune-correlates analysis of an HIV-1 vaccine efficacy trial. N Engl J Med. 2012;366(14):1275–86. Epub 2012/04/06. 10.1056/NEJMoa1113425 . 22475592 10.1056/NEJMoa1113425 PMC3371689

[6] Churchyard GJ, Morgan C, Adams E, Hural J, Graham BS, Moodie Z, et al A phase IIA randomized clinical trial of a multiclade HIV-1 DNA prime followed by a multiclade rAd5 HIV-1 vaccine boost in healthy adults (HVTN204). PLoS One. 2011;6(8):e21225 Epub 2011/08/23. 10.1371/journal.pone.0021225 PONE-D-11-04311 [pii]. . 21857901 10.1371/journal.pone.0021225 PMC3152265

[7] Excler JL, Tomaras GD, Russell ND. Novel directions in HIV-1 vaccines revealed from clinical trials. Curr Opin HIV AIDS. 2013;8(5):421–31. Epub 2013/06/08. 10.1097/COH.0b013e3283632c26 . 23743791 10.1097/COH.0b013e3283632c26 PMC5420453

[8] Goepfert PA, Elizaga ML, Sato A, Qin L, Cardinali M, Hay CM, et al Phase 1 safety and immunogenicity testing of DNA and recombinant modified vaccinia Ankara vaccines expressing HIV-1 virus-like particles. J Infect Dis. 2011;203(5):610–9. Epub 2011/02/02. jiq105 [pii] 10.1093/infdis/jiq105 . 21282192 10.1093/infdis/jiq105 PMC3072720

[9] Harari A, Bart PA, Stohr W, Tapia G, Garcia M, Medjitna-Rais E, et al An HIV-1 clade C DNA prime, NYVAC boost vaccine regimen induces reliable, polyfunctional, and long-lasting T cell responses. J Exp Med. 2008;205(1):63–77. Epub 2008/01/16. jem.20071331 [pii] 10.1084/jem.20071331 . 18195071 10.1084/jem.20071331 PMC2234371

[10] Mehendale S, Thakar M, Sahay S, Kumar M, Shete A, Sathyamurthi P, et al Safety and immunogenicity of DNA and MVA HIV-1 subtype C vaccine prime-boost regimens: a phase I randomised Trial in HIV-uninfected Indian volunteers. PLoS One. 2013;8(2):e55831 Epub 2013/02/19. 10.1371/journal.pone.0055831 PONE-D-12-18429 [pii]. . 23418465 10.1371/journal.pone.0055831 PMC3572184

[11] Bakari M, Aboud S, Nilsson C, Francis J, Buma D, Moshiro C, et al Broad and potent immune responses to a low dose intradermal HIV-1 DNA boosted with HIV-1 recombinant MVA among healthy adults in Tanzania. Vaccine. 2011;29(46):8417–28. Epub 2011/08/26. S0264-410X(11)01210-2 [pii] 10.1016/j.vaccine.2011.08.001 . 21864626 10.1016/j.vaccine.2011.08.001 PMC4795940

[12] Munseri PJ, Kroidl A, Nilsson C, Joachim A, Geldmacher C, Mann P, et al Priming with a simplified intradermal HIV-1 DNA vaccine regimen followed by boosting with recombinant HIV-1 MVA vaccine is safe and immunogenic: a phase IIa randomized clinical trial. PLoS One. 2015;10(4):e0119629 Epub 2015/04/16. 10.1371/journal.pone.0119629 PONE-D-14-37940 [pii]. . 25875843 10.1371/journal.pone.0119629 PMC4398367

[13] Nilsson C, Hejdeman B, Godoy-Ramirez K, Tecleab T, Scarlatti G, Brave A, et al HIV-DNA Given with or without Intradermal Electroporation Is Safe and Highly Immunogenic in Healthy Swedish HIV-1 DNA/MVA Vaccinees: A Phase I Randomized Trial. PLoS One. 2015;10(6):e0131748 Epub 2015/06/30. 10.1371/journal.pone.0131748 PONE-D-14-51346 [pii]. . 26121679 10.1371/journal.pone.0131748 PMC4486388

[14] Viegas EO, Tembe N, Nilsson C, Meggi B, Maueia C, Augusto O, et al Intradermal HIV-1 DNA Immunization Using Needle-Free Zetajet Injection Followed by HIV-Modified Vaccinia Virus Ankara Vaccination Is Safe and Immunogenic in Mozambican Young Adults: A Phase I Randomized Controlled Trial. AIDS Res Hum Retroviruses. 2017 Epub 2017/10/04. 10.1089/AID.2017.0121 . 28969431 10.1089/aid.2017.0121 PMC6913121

[15] Joachim A, Nilsson C, Aboud S, Bakari M, Lyamuya EF, Robb ML, et al Potent functional antibody responses elicited by HIV-I DNA priming and boosting with heterologous HIV-1 recombinant MVA in healthy Tanzanian adults. PLoS One. 10(4):e0118486 Epub 2015/04/16. 10.1371/journal.pone.0118486 PONE-D-14-36854 [pii]. . 25874723 10.1371/journal.pone.0118486 PMC4396991

[16] Sandstrom E, Nilsson C, Hejdeman B, Brave A, Bratt G, Robb M, et al Broad immunogenicity of a multigene, multiclade HIV-1 DNA vaccine boosted with heterologous HIV-1 recombinant modified vaccinia virus Ankara. J Infect Dis. 2008;198(10):1482–90. Epub 2008/09/24. 10.1086/592507 . 18808335 10.1086/592507 PMC4793972

[17] Joachim A, Munseri PJ, Nilsson C, Bakari M, Aboud S, Lyamuya EF, et al Three-Year Durability of Immune Responses Induced by HIV-DNA and HIV-Modified Vaccinia Virus Ankara and Effect of a Late HIV-Modified Vaccinia Virus Ankara Boost in Tanzanian Volunteers. AIDS Res Hum Retroviruses. 2017;33(8):880–8. Epub 2016/12/29. 10.1089/AID.2016.0251 . 28027665 10.1089/aid.2016.0251 PMC5564012

[18] Nilsson C, Godoy-Ramirez K, Hejdeman B, Brave A, Gudmundsdotter L, Hallengard D, et al Broad and potent cellular and humoral immune responses after a second late HIV-modified vaccinia virus ankara vaccination in HIV-DNA-primed and HIV-modified vaccinia virus Ankara-boosted Swedish vaccinees. AIDS Res Hum Retroviruses. 2014;30(3):299–311. Epub 2013/10/05. 10.1089/AID.2013.0149 . 24090081 10.1089/aid.2013.0149 PMC3938943

[19] Hirao LA, Wu L, Khan AS, Satishchandran A, Draghia-Akli R, Weiner DB. Intradermal/subcutaneous immunization by electroporation improves plasmid vaccine delivery and potency in pigs and rhesus macaques. Vaccine. 2008;26(3):440–8. Epub 2007/12/18. S0264-410X(07)01158-9 [pii] 10.1016/j.vaccine.2007.10.041 . 18082294 10.1016/j.vaccine.2007.10.041

[20] Kulkarni V, Rosati M, Bear J, Pilkington GR, Jalah R, Bergamaschi C, et al Comparison of intradermal and intramuscular delivery followed by in vivo electroporation of SIV Env DNA in macaques. Hum Vaccin Immunother. 2013;9(10):2081–94. Epub 2013/07/03. 25473 [pii] 10.4161/hv.25473 . 23811579 10.4161/hv.25473 PMC3906392

[21] Kalams SA, Parker SD, Elizaga M, Metch B, Edupuganti S, Hural J, et al Safety and comparative immunogenicity of an HIV-1 DNA vaccine in combination with plasmid interleukin 12 and impact of intramuscular electroporation for delivery. J Infect Dis. 2013;208(5):818–29. Epub 2013/07/11. jit236 [pii] 10.1093/infdis/jit236 . 23840043 10.1093/infdis/jit236 PMC3733506

[22] Vasan S, Hurley A, Schlesinger SJ, Hannaman D, Gardiner DF, Dugin DP, et al In vivo electroporation enhances the immunogenicity of an HIV-1 DNA vaccine candidate in healthy volunteers. PLoS One. 2011;6(5):e19252 Epub 2011/05/24. 10.1371/journal.pone.0019252 PONE-D-11-02192 [pii]. . 21603651 10.1371/journal.pone.0019252 PMC3095594

[23] Bolhassani A. Which Vaccination Strategies and Immune Responses are More Effective Against HIV Infections? In: Saxena SK, editor. Advances in Molecular Retrovirology: InTech; 2016 10.1186/s12977-016-0269-6

[24] Pitisuttithum P, Gilbert P, Gurwith M, Heyward W, Martin M, van Griensven F, et al Randomized, double-blind, placebo-controlled efficacy trial of a bivalent recombinant glycoprotein 120 HIV-1 vaccine among injection drug users in Bangkok, Thailand. J Infect Dis. 2006;194(12):1661–71. Epub 2006/11/17. JID36424 [pii] 10.1086/508748 . 17109337 10.1086/508748

[25] Flynn NM, Forthal DN, Harro CD, Judson FN, Mayer KH, Para MF. Placebo-controlled phase 3 trial of a recombinant glycoprotein 120 vaccine to prevent HIV-1 infection. J Infect Dis. 2005;191(5):654–65. Epub 2005/02/03. JID33333 [pii] 10.1086/428404 . 15688278 10.1086/428404

[26] Joachim A, Bauer A, Joseph S, Geldmacher C, Munseri PJ, Aboud S, et al Boosting with Subtype C CN54rgp140 Protein Adjuvanted with Glucopyranosyl Lipid Adjuvant after Priming with HIV-DNA and HIV-MVA Is Safe and Enhances Immune Responses: A Phase I Trial. PLoS One. 2016;11(5):e0155702 Epub 2016/05/19. 10.1371/journal.pone.0155702 PONE-D-15-55482 [pii]. . 27192151 10.1371/journal.pone.0155702 PMC4871571

[27] Mann P MP, Missanga M, Lwakatare J, Janabi M, Kapesa E, Robb ML, Hoelscher M, McCormack S, Bakari M, Maboko L, Sandström E, Kroidl A. High prevalence of ST-elevation, early repolarization, and left ventricular hypertrophy during the eligibility assessment for an HIV vaccine trial in young, healthy Tanzanians. Clinical Trials and Regulatory Science in Cardiology. 2017;26:1–6.

[28] Brave A, Ljungberg K, Boberg A, Rollman E, Isaguliants M, Lundgren B, et al Multigene/multisubtype HIV-1 vaccine induces potent cellular and humoral immune responses by needle-free intradermal delivery. Mol Ther. 2005;12(6):1197–205. Epub 2005/08/23. S1525-0016(05)01026-9 [pii] 10.1016/j.ymthe.2005.06.473 . 16112909 10.1016/j.ymthe.2005.06.473

[29] Earl PL, Cotter C, Moss B, VanCott T, Currier J, Eller LA, et al Design and evaluation of multi-gene, multi-clade HIV-1 MVA vaccines. Vaccine. 2009;27(42):5885–95. Epub 2009/08/06. S0264-410X(09)01050-0 [pii] 10.1016/j.vaccine.2009.07.039 . 19654066 10.1016/j.vaccine.2009.07.039 PMC2743792

[30] Su L, Graf M, Zhang Y, von Briesen H, Xing H, Kostler J, et al Characterization of a virtually full-length human immunodeficiency virus type 1 genome of a prevalent intersubtype (C/B') recombinant strain in China. J Virol. 2000;74(23):11367–76. Epub 2000/11/09. . 11070037 10.1128/jvi.74.23.11367-11376.2000 PMC113242

[31] Rodenburg CM, Li Y, Trask SA, Chen Y, Decker J, Robertson DL, et al Near full-length clones and reference sequences for subtype C isolates of HIV type 1 from three different continents. AIDS Res Hum Retroviruses. 2001;17(2):161–8. Epub 2001/02/15. 10.1089/08892220150217247 . 11177395 10.1089/08892220150217247

[32] Clegg CH, Roque R, Perrone LA, Rininger JA, Bowen R, Reed SG. GLA-AF, an emulsion-free vaccine adjuvant for pandemic influenza. PLoS One. 2014;9(2):e88979 Epub 2014/02/20. 10.1371/journal.pone.0088979 PONE-D-13-48976 [pii]. . 24551202 10.1371/journal.pone.0088979 PMC3925208

[33] (DAIDS) DoA. Table for Grading The Severity of Adult and Pediatric Adverse Events. 2004 Clarification August 2009;Version 1.0.

[34] Tembe N, Joaquim O, Alfai E, Sitoe N, Viegas E, Macovela E, et al Reference values for clinical laboratory parameters in young adults in Maputo, Mozambique. PLoS One. 2014;9(5):e97391 Epub 2014/05/16. 10.1371/journal.pone.0097391 PONE-D-14-04918 [pii]. . 24827458 10.1371/journal.pone.0097391 PMC4020854

[35] Bauer A, Podola L, Mann P, Missanga M, Haule A, Sudi L, et al Preferential Targeting of Conserved Gag Regions after Vaccination with a Heterologous DNA Prime-Modified Vaccinia Virus Ankara Boost HIV-1 Vaccine Regimen. J Virol. 2017;91(18). Epub 2017/07/14. 10.1128/JVI.00730-17 ; PubMed Central PMCID: PMCPMC5571275. 28701395 10.1128/JVI.00730-17 PMC5571275

[36] Agresti A, Caffo B. Simple and effective confidence intervals for proportions and differences of proportions result from adding two successes and two failures. The American Statistician 2000;54:280–88.

[37] Joseph S, Quinn K, Greenwood A, Cope AV, McKay PF, Hayes PJ, et al A Comparative Phase I Study of Combination, Homologous Subtype-C DNA, MVA, and Env gp140 Protein/Adjuvant HIV Vaccines in Two Immunization Regimes. Front Immunol. 2017;8:149 10.3389/fimmu.2017.00149 ; PubMed Central PMCID: PMCPMC5319954. 28275375 10.3389/fimmu.2017.00149 PMC5319954

[38] Eller LA, Eller MA, Ouma B, Kataaha P, Kyabaggu D, Tumusiime R, et al Reference intervals in healthy adult Ugandan blood donors and their impact on conducting international vaccine trials. PLoS One. 2008;3(12):e3919 Epub 2008/12/17. 10.1371/journal.pone.0003919 . 19079547 10.1371/journal.pone.0003919 PMC2593783

[39] Haddy TB, Rana SR, Castro O. Benign ethnic neutropenia: what is a normal absolute neutrophil count? J Lab Clin Med. 1999;133(1):15–22. Epub 1999/06/29. S0022-2143(99)00013-X [pii] 10.1053/lc.1999.v133.a94931 . 10385477 10.1053/lc.1999.v133.a94931

[40] Kibaya RS, Bautista CT, Sawe FK, Shaffer DN, Sateren WB, Scott PT, et al Reference ranges for the clinical laboratory derived from a rural population in Kericho, Kenya. PLoS One. 2008;3(10):e3327 Epub 2008/10/04. 10.1371/journal.pone.0003327 . 18833329 10.1371/journal.pone.0003327 PMC2553265

[41] Ake JA, Schuetz A, Pegu P, Wieczorek L, Eller MA, Kibuuka H, et al Safety and Immunogenicity of PENNVAX(R)-G DNA Prime Administered by Biojector(R) 2000 or CELLECTRA(R) Electroporation Device with Modified Vaccinia Ankara-CMDR Boost. J Infect Dis. 2017 Epub 2017/10/03. 4102981 [pii] 10.1093/infdis/jix456 . 28968759 10.1093/infdis/jix456 PMC5853809

