# Supplementary material for: Optimizing the immunogenicity of HIV prime-boost DNA-MVA-rgp140/GLA vaccines in a phase II randomized factorial trial design
Source: PLoS One. 2018 Nov 29;13(11):e0206838. doi: 10.1371/journal.pone.0206838 (PMC6264478; doi:10.1371/journal.pone.0206838)
Supplement: S1 Table — (DOCX) [file pone.0206838.s002.docx]

**S1 Table 1**. Vaccines composition

| **Vaccines** | **Composition and Derivation** | **References** |
| --- | --- | --- |
| HIVIS-DNA  Lot #20120928-24:3/42-8 (Groups I, II)  Lot #20120926-24:3/42-8 (Group III)  Manufactured by Vecura (Huddinge, Stockholm, Sweden) | Pool 1 (Env gp160 A,B,C and Rev B)  Pool 2 (Gag p37 A,B and Rtmut B) | 16, 28 |
| HIV-MVA  Lot #08560313  Manufactured by WRAIR Pilot Bio production facility (Forest Glen, Maryland, USA) | CRF01_AE isolates from Chiang Mai (CM), Thailand  Env gp150 E (derived from isolate HIV-1 CM235)  Gag and Pol A (derived from isolate HIV-1 CM240) | 29 |
| CN54rgp140  Lot #T920711A  Manufactured by Polymun Scientific (Vienna, Austria) | Subtype-C/B’ p97CN54 from Chinese isolate 97CM001  Env gp140 (gp120 plus ectodomain of gp41) | 30, 31 |
| GLA-AF  Manufactured by Infectious Disease Research Institute (IDRI, Seattle, WA, USA). | Glucopyranosyl lipid A in an acqueous formulation, concentration of 25μg/mL | 32 |
