# Supplementary material for: Optimizing the immunogenicity of HIV prime-boost DNA-MVA-rgp140/GLA vaccines in a phase II randomized factorial trial design
Source: PLoS One. 2018 Nov 29;13(11):e0206838. doi: 10.1371/journal.pone.0206838 (PMC6264478; doi:10.1371/journal.pone.0206838)
Supplement: S2 Table — (DOCX) [file pone.0206838.s003.docx]

| **S2 Table. Baseline characteristics for participants included in the modified intent-to-treat analysis (mITT), overall and by randomization group.** | | | | | | | |
| --- | --- | --- | --- | --- | --- | --- | --- |
|  |  | **HIV-DNA vaccine recipients (N=191)** | | | **HIV-MVA +/- CN54rgp140/GLA-AF vaccine recipients (N=152)** | |  |
| **Characteristics** | **Total**  **N=211** | **Group I**  **N=62** | **Group II**  **N=63** | **Group III**  **N=66** | **MVA + Protein**  **N=71** | **MVA**  **N=81** | **Placebo**  **N=20** |
| Site |  |  |  |  |  |  |  |
| NIMR-MMRC | 89 (42.2) | 27 (43.5) | 26 (41.3) | 28 (42.4) | 29 (40.9) | 30 (37.1) | 8 (40.0) |
| MUHAS | 82 (38.8) | 23 (37.1) | 25 (39.7) | 25 (37.9) | 28 (39.4) | 33 (40.7) | 9 (45.0) |
| CISPOC | 40 (19.0) | 12 (19.4) | 12 (19.0) | 13 (19.7) | 14 (19.7) | 18 (22.2) | 3 (15.0) |
| Sex |  |  |  |  |  |  |  |
| Female | 97 (46.0) | 28 (45.2) | 30 (47.6) | 30 (45.5) | 29 (40.9) | 33 (40.7) | 9 (45.0) |
| Age (years) | 22 (20-26) | 23 (20-25) | 22 (20-25) | 23 (20-28) | 23 (20-27) | 22 (20-26) | 21 (19-23) |
| Vaccinia scar present | 68 (32.2) | 19 (30.7) | 21 (33.3) | 22 (33.3) | 22 (31.0) | 24 (29.6) | 6 (30.0) |
| BMI (kg/m^2^) | 22 (20-23) | 22 (20-24) | 22 (20-24) | 22 (20-23) | 22 (20-24) | 21 (19-23) | 20 (19-21) |
| Laboratory |  |  |  |  |  |  |  |
| Hemoglobin (g/dL) | 14.0 (12.5-15.4) | 13.9 (12.5-15.6) | 13.6 (12.5-15.2) | 14.1 (12.8-15.5) | 13.9 (12.4-15.5) | 14.1 (12.7-15.4) | 14.4 (12.2-15.7) |
| White cell count (10^9^ cells/l) | 4.7 (3.8-5.4) | 4.6 (3.7-5.7) | 4.8 (3.8-5.6) | 4.5 (3.9-5.2) | 4.6 (3.6-5.5) | 4.7 (4.0-5.3) | 4.8 (3.9-5.1) |
| Neutrophils (10^9^ cells/L) | 2.3 (1.7-2.8) | 2.3 (1.6-2.7) | 2.6 (1.8-3.2) | 2.1 (1.6-2.6) | 2.3 (1.8-2.7) | 2.4 (1.6-2.9) | 2.2 (1.7-2.8) |
| Lymphocytes (10^9^ cells/L) | 1.8 (1.5-2.2) | 1.7 (1.5-2.2) | 1.8 (1.4-2.2) | 1.8 (1.5-2.1) | 1.7 (1.5-2.0) | 1.8 (1.4-2.2) | 2.0 (1.6-2.3) |
| Platelets (10^9^ cells/L) | 251 (208-303) | 237 (189-294) | 258 (208-295) | 260 (218-300) | 251 (207-302) | 250 (208-291) | 255 (214-328) |
| CD4^+^ cell count (10^6^ cells/L) | 687 (561-851) | 660 (517-807) | 671 (560-808) | 696 (561-882) | 660 (500-851) | 678 (542-844) | 731 (640-764) |
| ALT (U/L) | 13 (10-19) | 16 (11-20) | 13 (10-17) | 13 (10-19) | 15 (11-19) | 14 (11-20) | 12 (10-19) |
| Total Bilirubin (µmol/L) | 8 (6-12) | 8 (6-12) | 8 (6-12) | 8 (6-12) | 8 (6-12) | 9 (6-12) | 8 (5-14) |
| Creatinine (µmol/L) | 58 (48-66) | 58 (48-67) | 57 (48-68) | 58 (51-66) | 60 (52-66) | 59 (50-69) | 56 (47-62) |
| Glucose (mmol/L) | 4.4 (4.0-4.7) | 4.4 (4.0-4.7) | 4.4 (4.0-4.7) | 4.3 (3.9-4.8) | 4.3 (3.9-4.7) | 4.4 (4.0-4.8) | 4.2 (3.9-4.5) |

Note: Values are numbers (%) or medians (interquartile ranges)
