# Supplementary material for: Optimizing the immunogenicity of HIV prime-boost DNA-MVA-rgp140/GLA vaccines in a phase II randomized factorial trial design
Source: PLoS One. 2018 Nov 29;13(11):e0206838. doi: 10.1371/journal.pone.0206838 (PMC6264478; doi:10.1371/journal.pone.0206838)
Supplement: S3 Table — P-values are given for the comparisons of events between vaccine groups (chi-square/Fisher’s exact test). (DOCX) [file pone.0206838.s004.docx]

| **S3 Table. Non-solicited adverse events and laboratory adverse events indicated for the total population, and vaccine and placebo recipients over the entire study period. P-values are given for the comparisons of events between vaccine groups (chi-square/Fisher’s exact test).** | | | | | | | | | | | | |
| --- | --- | --- | --- | --- | --- | --- | --- | --- | --- | --- | --- | --- |
| **Non-solicited AE** | **Total**  **211** | **Vaccine**  **N=191** | **Placebo**  **N=20** | **Any DNA**  **N=191** | **Group I**  **N=62** | **Group II**  **N=63** | **Group III**  **N=66** | **p-value** | **Any MVA**  **N=152** | **MVA**  **N=81** | **MVA/p**  **N=71** | **p-value** |
| Overall number of adverse events | 511 | 470 | 41 | 331 | 91 | 135 | 105 |  | 136 | 81 | 55 |  |
| Any adverse event | 159 (75.4) | 143 (74.9) | 16 (80.0) | 123 (64.4) | 36 (58.1) | 46 (73.0) | 41 (62.1) | 0.19 | 70 (46.1) | 42 (51.9) | 28 (39.4) | 0.13 |
| Any adverse event within 28 days post vaccination | 126 (59.7) | 116 (60.7) | 10 (50.0) | 102 (53.4) | 30 (48.4) | 35 (55.6) | 37 (66.1) | 0.63 | 47 (30.9) | 28 (34.6) | 19 (26.8) | 0.30 |
| Any grade 3 or 4 adverse event | 6 (2.8) | 6 (3.1) | 0 | 6 (3.1) | 2 (3.2) | 4 (6.5) | 0 | 0.09 | 0 | 0 | 0 | - |
| Any grade 3 or 4 adverse event within 28 days post vaccination | 2 (1.0) | 2 (1.1) | 0 | 2 (1.1) | 1 (1.6) | 1 (1.6) | 0 | 0.55 | 0 | 0 | 0 | - |
| Adverse event related to vaccines§ | 9 (4.3) | 7 (3.7) | 2 (10.0) | 4 (2.1) | 1 (1.6) | 1 (1.6) | 2 (3.0) | 1.0 | 2 (1.3) | 0 | 2 (2.8) | 0.22 |
| HIV infections* | 4 (1.9) | 2 (1.0) | 2 (10.0) | 2 (1.0) | 1 (1.6) | 0 | 1 (1.5) | 0.77 | 0 | 0 | 0 | - |
| Serious adverse events† | 5 (2.4) | 5 (2.6) | 0 | 5 (2.6) | 1 (1.6) | 2 (3.2) | 2 (3.0) | 1.00 | 0 | 0 | 0 | - |
| Adverse event leading to vaccine discontinuation‡ | 10 (4.7) | 9 (4.7) | 1 (5.0) | 9 (4.7) | 3 (4.8) | 4 (6.4) | 2 (3.0) | 0.64 | 0 | 0 | 0 | - |
| Primary Safety endpoint** | 40 (19) | 35 (18) | 5 (25) | 30 (15) | 9 (14) | 13 (20) | 8 (12) | 0.42 | 11 (7) | 7 (9) | 4 (6) | 0.48 |
| **Laboratory adverse events** | **Total**  **209^¶^** | **Vaccine**  **N=190^¶^** | **Placebo**  **N=19^¶^** | **Any DNA**  **N=190^¶^** | **Group I**  **N=62** | **Group II**  **N=62^¶^** | **Group III**  **N=66** | **p-value** | **Any MVA**  **N=152** | **MVA**  **N=81** | **MVA/p**  **N=71** | **p-value** |
| Any grade lab AE | 137 (65.6) | 122 (64.2) | 15 (78.9) | 100 (52.6) | 32 (51.6) | 36 (58.1) | 32 (48.5) | 0.55 | 72 (47.4) | 44 (54.3) | 28 (39.4) | 0.07 |
| Any grade AE within 28 days post vaccination | 112 (53.6) | 97 (51.1) | 15 (78.9) | 78 (41.1) | 24 (38.7) | 28 (45.2) | 26 (39.4) | 0.72 | 58 (38.2) | 36 (44.4) | 22 (31.0) | 0.09 |
| Any grade 3 or 4 lab AE within 28 days post vaccination | 17 (8.1) | 14 (7.4) | 3 (15.8) | 8 (4.2) | 3 (4.8) | 3 (4.8) | 2 (3.0) | 0.82 | 7 (4.6) | 4 (4.9) | 3 (4.2) |  |
| - Anaemia, <9 g/dL | 2 (1.0) | 2 (1.1) | 0 | 1 (0.5) | 1 (1.6) | 0 | 0 | - | 1 (0.7) | 1 (0.7) | 0 | - |
| - Neutropenia, <750 cells/µL | 13 (6.2) | 10 (5.3) | 3 (15.8) | 7 (3.7) | 2 (3.2) | 3 (4.8) | 2 (3.0) | 0.84 | 4 (2.6) | 3 (3.7) | 1 (1.4) | 0.38 |
| - Bilirubinaemia, >2.5 x ULN | 2 (1.0) | 2 (1.1) | 0 | 0 | 0 | 0 | 0 | - | 2 (1.4) | 0 | 2 (2.8) | - |

**Note:** Apart from the first row which indicates the total number of event across participants, the number (%) of participants with a particular event is presented. Events within the DNA-priming groups (Group I: 2x 0.1 mL ID [3mg/mL], Group II: 2x 0.1 mL ID + electroporation [3mg/mL], Group III: 1x 0.1 mL ID + electroporation [6mg/mL]) indicate only those which occurred before the first vaccine boost, events which occurred after boosting with MVA alone or MVA plus rgp140/GLA-AF indicate only those that occurred after the first vaccine boost.

# All AEs were grade III, considered not related to vaccinations and included hypochromic microcytic anaemia (N=1), cannabis induced psychosis (N=1), malaria (N=2), abortion (N=1), fracture of the mandibular (N=1)

§ Overall 10 related events experienced by 9 participants were either mild or moderate and not serious. Possibly related events (N=8) occurred in 7 participants and included after DNA priming: vesicles on the tongue (N=1) and dizziness (N=1) in the same participant and time-point (Group I); vesicular rash (N=1, Group III), lumbar pain (N=1, Group II); after MVA + gp140/GLA boosting: chest pain (N=1), flu like disease (N=1); in the placebo groups: acute gastroenteritis (N=1), bullous skin lesion (N=1). Definitely related events (N=2) included induration and redness at the injection site (N=1, Group 3 post DNA-priming) and social harm (girlfriend separated because of trial participation) before any vaccine administration (N=1, Group III)).

*HIV-infection occurred (i) after the 3^rd^ HIV-DNA immunization (Group I), (ii) after the 1^st^ HIV-DNA immunization (Group III), and in two placebo recipients (iii) after the first boost, and (iv) after completion of all immunizations.

†all SAE were due to hospitalizations, considered not related to the vaccine regimen and included malaria (N=3), cannabis induced psychosis (N=1), acute HIV infection (N=1).

‡ all AEs leading to discontinuations were considered not related to the vaccine regimen and included hyperthyroidism (N=2), pulmonary tuberculosis (N=1), arterial hypertension (N=1), breast lumps (fibroadenoma) (N=1), cannabis induced psychosis (N=1), acute HIV infection (N=3), hypochromic microcytic anaemia (N=1).

¶ N=2 participants never received blood draw due to very early drop-out of the study.

** Primary safety endpoint: any grade 3 or above local or systemic clinical and laboratory solicited AEs or any grade of AE that resulted in a clinical decision to discontinue immunization.
