# Supplementary figures and images for: Optimizing the immunogenicity of HIV prime-boost DNA-MVA-rgp140/GLA vaccines in a phase II randomized factorial trial design
Source: PLoS One. 2018 Nov 29;13(11):e0206838. doi: 10.1371/journal.pone.0206838 (PMC6264478; doi:10.1371/journal.pone.0206838)

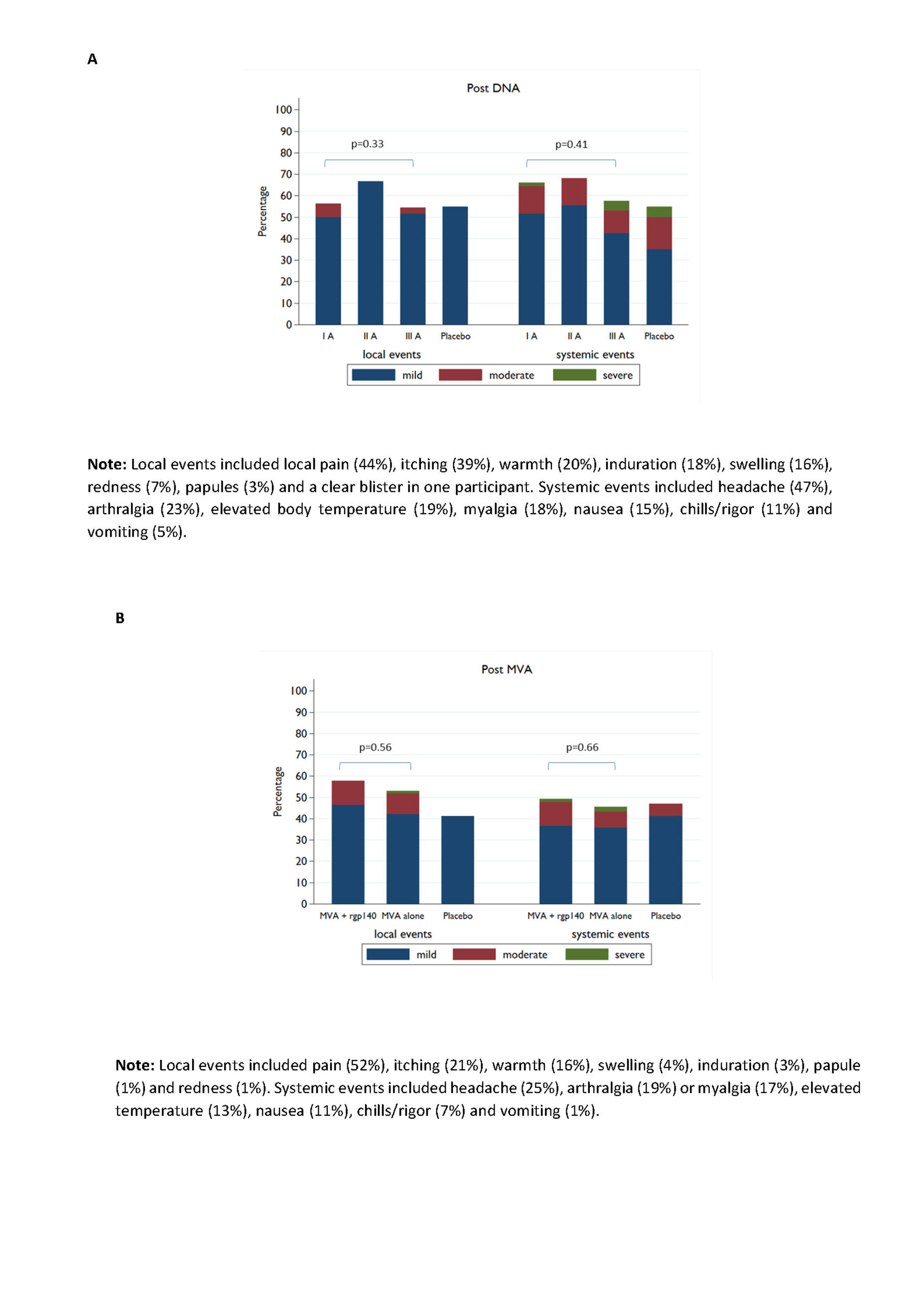

Supplement: S1 Fig — (A) After HIV-DNA priming (Group I: 2x 0.1 mL ID [3mg/mL], Group II: 2x 0.1 mL ID + electroporation [3mg/mL], Group III: 1x 0.1 mL ID + electroporation [6mg/mL]), and (B) after HIV-MVA boost alone or HIV-MVA plus rgp140/GLA-AF. P-values are given for the comparisons of any event between experimental vaccine groups (chi-square/Fisher’s exact test). (TIF) [file pone.0206838.s005.tif]
