# Supplementary material for: Optimizing the immunogenicity of HIV prime-boost DNA-MVA-rgp140/GLA vaccines in a phase II randomized factorial trial design
Source: PLoS One. 2018 Nov 29;13(11):e0206838. doi: 10.1371/journal.pone.0206838 (PMC6264478; doi:10.1371/journal.pone.0206838)
Supplement: S1 File — (PDF) [file pone.0206838.s006.pdf]

TaMoVac II

A Phase II trial to assess the safety and immunogenicity of DNA priming administered by the ID Zetajet® with or without ID Derma Vax™ electroporation followed by IM MVA boosting with or without CN54 rgp140/GLA-AF in healthy volunteers in Tanzania and Mozambique

The trial is funded by the European and Developing Countries Clinical Trials Partnership Programme (EDCTP)

The Swedish Institute for Communicable Disease Control, SMI will act as the Sponsor

Clinical Trials.gov: NCT01697007
PACTR: PACTR201211000435126

Protocol, Version Number 2.0
Protocol date 15th April 2013

**Authorised by:**			
Name:	Dr Johan Carlson	Role:	Sponsor – SMI<br>Swedish Institute for Infectious Disease Control
Signature:		Date:	
Name:	Professor Ephata E. Kaaya	Role:	Contractor - MUHAS<br>Muhimbili University of Health & Allied sciences
Signature:		Date:	
Name:	Professor Eligius Lyamuya	Role:	Chief Investigator<br>MUHAS
Signature:		Date:	
Name:	Professor Kisali Pallangyo	Role:	Deputy-Chief Investigator,<br>MUHAS
Signature:		Date:	
Name:	Dr Leonard Maboko	Role:	Principal Investigator,<br>NIMR-MMRC
Signature:		Date:	
Name:	Dr Ilesh Jani	Role:	Principal Investigator,<br>Instituto Nacional de Saúde (INS) from Mozambique
Signature:		Date:	

GENERAL INFORMATION

This document describes the TaMoVac II trial and provides information about procedures for entering participants into it. Every care was taken in drafting the protocol, but corrections or amendments may be necessary. These will be circulated to the Principal Investigators in the trial, prior to implementation. Clinical problems relating to this trial should be referred to one of the Principal Investigators or the Medical Expert.

Compliance

The study will be conducted according to the principles of ICH GCP guidelines and the Declaration of Helsinki (Version 2008), and it is the responsibility of the Clinical Investigators to ensure adherence. Before the study starts all personnel involved in the clinical and laboratory trial units will be trained for Good Clinical Practice (GCP) and/or Good Laboratory Practice (GLP) respectively. Study personnel will also be trained on standard operating procedures (SOPs) that are related to the protocol and will follow the GCP and GLP guidelines.

- ## Trial Sponsor

Prof. Johan Carlson (MD, PhD)
Swedish Institute for Communicable Disease Control
Nobels väg 18
171 82
Solna, Sweden
Tel: +46 8 457 2310
Fax: +46 8 30 36 68
E-mail: johan.carlson@smi.se

- ## Contractor

Prof. Ephata E. Kaaya,
Vice Chancellor,
Muhimbili University of Health and Allied Sciences,
Box 65001,
Dar Es Salaam.
Tanzania.
Email: ekaaya@muhas.ac.tz and ephata13.kaaya@gmail.com

- ## Funder

European and Developing Countries Clinical Trials Partnership Programme (EDCTP

- ## Main Contacts:
- **Chief Investigator**

Prof. Eligius Lyamuya
Muhimbili University of Health and Allied Sciences,
Department of Microbiology/Immunology,
P.O. Box 65001, Dar es Salaam
Tel: +255 (22) 2152489 or +255 (754) 495933
Email: elyamuya@muhas.ac.tzandeligius_lyamuya@yahoo.com

- **Deputy Chief Investigator**

Prof. Kisali Pallangyo
Muhimbili University of Health and Allied Sciences,
School of Medicine,
P.O. Box 65001, Dar es Salaam
Tel: +255 713 224052
Email: kpallangyo@muhas.ac.tz and kpallangyo@gmail.com

- **Principal Investigator, MUHAS**

Prof. Muhammad Bakari
Muhimbili University of Health and Allied Sciences (MUHAS)
Department of Internal Medicine,
P.O. Box 65001,
Dar es Salaam, Tanzania
Tel: 255 713 702211
Email: drbakari@yahoo.com

- **Principal Investigator, NIMR-MMRC**

Dr. Leonard Maboko (MD, MSc)
NIMR-Mbeya Medical Research Center
P.O. Box 2410,
Mbeya, Tanzania
Tel: 255 025 2503364
Email: lmaboko@mmrp.org

- **Principal Investigator, CISPOC**

Dr. Ilesh Jani (MD, PhD)
Instituto Nacional de Saúde
Ministério da Saúde
Maputo, Mozambique
E-mail: ilesh.jani@gmail.com
Phone: +258 827453820

- **Protocol Chair and Sponsor's Medical Expert**

Prof. Eric Sandström (MD, PhD)
Venhälsan/ Karolinska Institutet
Södersjukhuset
118 83 Stockholm, Sweden
Email: eric.sandstrom@sodersjukhuset.se
Phone: + 46 8 616 2571

- **Protocol Co-Chairs**

Dr. Arne Kroidl, MD
Klinikum der Universität München
Department for Infectious Diseases and Tropical Medicine
Section International Medicine and Public Health
Leopoldstrasse 5
80805 Munich, Germany
Email: akroidl@mmrp.org
Phone: +49 89 2180 17616

Prof. Sheena McCormack MSc, FRCP
Senior Clinical Scientist
Medical Research Council Clinical Trials Unit (MRC CTU)
Aviation House
125 Kingsway, London
Phone: +44 20 7670 4708
E-mail: smc@ctu.mrc.ac.uk

- **Co-Investigators**

Dr. Patricia Munseri, MD
Clinical Research Coordinator
Muhimbili University of Health and Allied Sciences (MUHAS)
Department of Internal Medicine,
P.O. Box 65001,
Dar es Salaam, Tanzania
Tel: 255 754 562784
Email: pmunseri@yahoo.com

Dr. Philipp Mann, MD
Clinical Research Coordinator
NIMR- Mbeya Medical Research Center
Hospital Hill
PO Box 2410, Mbeya, Tanzania
Email: pmann@mmrp.org
Telephone: +255 25 2503364/Fax: +255 25 2503134

Dr. Marco Missanga, MD
Co- Clinical Research Coordinator
NIMR- Mbeya Medical Research Center
Hospital Hill
PO Box 2410, Mbeya, Tanzania
Email: mmissanga@mmrp.org
Telephone: +255 25 2503364/Fax: +255 25 2503134

Dr. Bahati Kaluwa, MD
NIMR- Mbeya Medical Research Center
Hospital Hill
PO Box 2410, Mbeya, Tanzania
Email: bkaluwa@mmrp.org
Telephone: +255 25 2503364/Fax: +255 25 2503134

Dr. Emmanuel Kapesa, MD
NIMR-Mbeya Medical Research Center

Hospital Hill
PO Box 2410, Mbeya, Tanzania
Email: ekapesa@mmrp.org
Telephone: +255 25 2503364/Fax: +255 25 2503134

Dr. Nafissa Osman, MD PhD
Department of Gynecology and Obstetrics
Hospital Central de Maputo
Maputo, Mozambique
E-mail: nafissa.osman@gmail.com
Phone:+258 823102170

Dr. Edna Viegas, MD
Clinical Research Coordinator
Centro de Investigação e Treino em Saúde da Polana Caniço (CISPOC)
Instituto Nacional de Saúde, Ministério da Saúde
Maputo, Mozambique
E-mail: ednaviegas@gmail.com
Phone: +258 823060500

Dr. Nilesh Bhatt, MD
Centro de Investigação e Treino em Saúde da Polana Caniço (CISPOC)
Instituto Nacional de Saúde, Ministério da Saúde
Maputo, Mozambique
E-Mail: nbhatt.mz@gmail.com
Phone: +258 823861130

Prof. Michael Hoelscher, MD, PhD
Klinikum der Universität München
Department for Infectious Diseases and Tropical Medicine
Section International Medicine and Public Health
Leopoldstrasse 5
80805 Munich, Germany
Tel: +49 89 2180 17601 /Fax: +49 89 336038
Email: hoelscher@lrz.uni-muenchen.de

Sarah Joseph, PhD
Medical Research Council Clinical Trials Unit (MRC CTU)
Aviation House
125 Kingsway, London
Phone: +44 20 76704670
Email: sjo@ctu.mrc.ac.uk

Professor Britta Wahren, MD PhD
Department of Virology
The Swedish Institute for Infectious Disease Control
171 82 Solna
Telephone: 00 46 8 457 2630; 004670 6741527
Fax: 00 46 8 337272
Email: Britta.Wahren@ki.se

- ## Consultancies

Dr. Merlin Robb, MD
United States Military HIV Research Program
Military HIV Research Program (MHRP)
6720-A Rockledge Drive, Suite 400
Bethesda, MD 20817
Email: mrobb@hivresearch.org

- ## Trial Pharmacists

Mr. Deus Buma, BPharm MPharm
Muhimbili National Hospital (MNH)
Pharmacy Department
P.O. Box 65000,
Dar es Salaam, Tanzania
Tel: 255 715 22 82 88, +255 757 32 75 51
Email: buma70@yahoo.co.uk

Mr. Revocatus Kunambi, BPharm
NIMR- Mbeya Medical Research Center
PO Box 2410,
Mbeya, Tanzania
Phone: +255 25 2503364/Fax: +255 25 2503134
Email: rkunambi@mmrp.org

Mrs. Bindiya Meggi, PharmD
Centro de Investigação e Treino em Saúde da Polana Caniço (CISPOC)
Instituto Nacional de Saúde, Ministério da Saúde
Maputo, Mozambique
Phone:+258 3583120
E-mail: bindiyameggi@gmail.com

- ## Data Management & Statistics

Trial Statistician:

Dr. Candida Moshiro PhD
Muhimbili University of Health and Allied Sciences (MUHAS)
P.O. Box 65001,
Dar es Salaam , Tanzania
Email: cmoshiro@yahoo.com
Phone: +255 78 495 0071

Statistical Mentor

Dr. Wolfgang Stöhr PhD
Medical Research Council Clinical Trials Unit (MRC CTU)
Aviation House
125 Kingsway, London
Phone: +44 (0) 207 670 4858
E-mail: W.Stoehr@ctu.mrc.ac.uk

Data Management

Dr. Sayoki Mfinanga MD
National Institute for Medical Research (NIMR)
P.O. Box 3436
Dar es Salaam, Tanzania
Telephone: +255 22 2152232
Email: gsmfinang@yahoo.com

Mr. Ayubu Masasi
National Institute for Medical Research (NIMR)
P.O. Box 3436
Dar es Salaam, Tanzania
Telephone: +255 22 2121400
Email: ayubumasasi@yahoo.com

Mr. John Mduda
National Institute for Medical Research (NIMR)
P.O. Box 3436
Dar es Salaam, Tanzania
Telephone: +255 714 693099
Email: j.mduda@hotmail.com

Mr. Dickens Kowuor
NIMR - Mbeya Medical Research Centre
Hospital Hill,
PO Box 2410, Mbeya, Tanzania
Telephone: +255 25 2503364/Fax: +255 25 2503134
Email: dkowuor@mmrp.org

Maria Patricia Gonçalves
Centro de Investigação e Treino em Saúde da Polana Caniço (CISPOC)
Instituto Nacional de Saúde, Ministério da Saúde
Maputo, Mozambique
Telephone: +258 82 7008539
Email: mariapatriciagoncalves@gmail.com

Data Management Mentors

Dr. Sue Fleck PhD
Medical Research Council Clinical Trials Unit (MRC CTU)
Aviation House
125 Kingsway, London
Phone: +44 (0) 207 670 4893
E-mail: slf@ctu.mrc.ac.uk

Elmar Saathoff, PhD
Klinikum der Universität München
Department for Infectious Diseases and Tropical Medicine
Section International Medicine and Public Health
Leopoldstrasse 5
80805 Munich, Germany
Tel: +49 89 2180 17601 /Fax: +49 89 336038
Email: saathoff@lrz.uni-muenchen.de

- ## Laboratories

MUHAS: ***The Department of Microbiology and Immunology, School of Medicine,***

Dr. Said Aboud, PhD
Lab coordinator
Muhimbili University of Health and Allied Sciences (MUHAS)
P.O. Box 65001,
Dar es Salaam, Tanzania
Tel: +255 (22) 2150302 or +255 (754) 302792
Email: aboudsaid@yaho.com

Dr Agricola Joachim, MD
Muhimbili University of Health Sciences (MUHAS)
P.O. Box 65001, Dar es Salaam, Tanzania
Tel: +255 713 295 717
Email: agricolaj@yahoo.com

NIMR- MMRC: ***The Clinical Research Laboratory at MMRC***

Dr. Asli Bauer
Immunologist and Lab Manager Immunological Lab Mbeya
NIMR-Mbeya Medical Research Center (MMRC)
PO Box 2410, Mbeya, Tanzania
Tel: +255 (25) 250 3364
Email: abauer@mmrp.org

Mrs. Cornelia Luer
NIMR-Mbeya Medical Research Center (MMRC)
Main laboratories
P.O. Box 2410, Mbeya, Tanzania.
Phone: 255 25 250 6164 / Fax: 255-25-250-3134
Email:cluermmrp.org

INS Maputo
Instituto Nacional de Saúde, Mozambique

Mr. Nelson Tembe
Lab coordinator
Instituto Nacional de Saúde, Ministério da Saúde
Maputo, Mozambique
E-mail: nelsontembe@yahoo.com.br
Phone:+258 823745020

Centro de Investigação e Treino em Saúde da Polana Caniço (CISPOC) laboratory
Mrs. Sandra Cândido
Lab coordinator
Centro de Investigação e Treino em Saúde da Polana Caniço (CISPOC)
Instituto Nacional de Saúde, Ministério da Saúde
Maputo, Mozambique
E-mail: sdrcandido@hotmail.com
Phone:+258 1201062

SMI Sweden
Prof. Gunnel Biberfeld, MD PhD
The Swedish Institute for Communicable Disease Control, SMI
Department of Diagnostics and Vaccinology
Nobels väg 18
171 82 Solna, Sweden
Phone: 00 46 8 457 2660
Email: gunnel.biberfeld@smi.se

Dr. Charlotta Nilsson, PhD
The Swedish Institute for Communicable Disease Control, SMI
Department of Diagnostics and Vaccinology
Nobels väg 18
171 82 Solna, Sweden
Phone: +46 8 4572612
Email: charlotta.nilsson@smi.se

LMU Germany
Dr. Christof Geldmacher, PhD
Klinikum der Universität München
Department for Infectious Diseases and Tropical Medicine
Section International Medicine and Public Health
Leopoldstrasse 5
80805 Munich, Germany
Tel: +49 89 2180 17614 /Fax: +49 89 336038
Email: geldmacher@lrz.uni-muenchen.de

Imperial College
Prof Francis Gotch, PhD
Imperial College, Chelsea & Westminster Hospital
Department of Immunology
369 Fulham Road, London SW10 9NH
Tel: +44 208 746 8257
Email:f.gotch@imperial.ac.uk.

- **External Monitor:**

Ms. Gail A. Smith,
Military HIV Research Program (MHRP)
6720-A Rockledge Drive, Suite 400
Bethesda, MD 20817
Email: gsmith@hivresearch.org

- **Vaccine suppliers**

DNA, MVA, CN54 rgp140 and GLA-AF will be supplied through:

Professor Britta Wahren, PhD
Department of Virology
The Swedish Institute for Infectious Disease Control
171 82 Solna
Telephone: 00 46 8 457 2630; 004670 6741527
Fax: 00 46 8 337272
Email: Britta.Wahren@ki.se

MVA will be donated to KI/SMI by the WRAIR under a Cooperative Research and Development Agreement with the Karolinska Institute/SMI

Tina Tong
Walter Reed Army Institute of Research (WRAIR), USMHRP (MHRP)
Military HIV Research Program (MHRP)
6720-A Rockledge Drive, Suite 400
Bethesda, MD 20817
Email: ttong@hivresearch.org

CN54 rgp140 and GLA-AF will be donated to KI/SMI by Imperial College London under a Material Transfer Agreement.

SAE AND IMPORTANT AE NOTIFICATION

Within 24 hours of becoming aware of an SAE or Important AE, please email, fax or telephone notification of the eventto Professors Eligius Lyamuya and Eric Sandstrom:

Tel: +255 (22) 2152489 or +255 (754) 495933
Fax: +255 (22) 2153027
Or email: elamuya@muhas.ac.tz
and
Eric Sandström Tel. +46 704 846037, Fax +46 8 616 2509 and eric.sandstrom@sodersjkuhuset.se

CONTENTS

1 Summary 17
1.1 Summary of trial design 17

2 Background 22
2.1 Background 22
2.2 Work leading to this trial: 25
2.3 Rationale for design and objectives 26
2.4 Investigational products/intervention(s) 27

3 Selection of Centres/Clinicians 31

4 Selection of Participants 33
4.1 Participant inclusion criteria 33
4.2 Participant exclusion criteria 34
4.3 Number and source of participants 35
4.4 Screening procedures and pre-randomisation investigations 36

5 Enrolment & Randomisation Procedure 39
5.1 Enrolment 39
5.2 Randomisation 39
5.3 Randomisation codes and unblinding 39
5.4 Co-enrolment guidelines 40
5.5 Replacements in the Enrolment and Randomisation Lists 40

6 Treatment of Participants 41
6.1 Vaccine products 41
6.2 Administration of vaccines 41
6.3 Accountability for used and unused supplies 42
6.4 Immunisation schedule 46
6.5 Dose modifications and discontinuation 47
6.6 Clinical management of adverse events 47
6.7 Non-trial treatment 47
6.8 Issues related to HIV 48

7 Assessments and follow-up 50
7.1 Duration of follow-up and schedule 50
7.2 Assessments at screening 51
7.3 Other laboratory assessments 52
7.4 Procedures for assessing immunogenicity 53
7.5 Procedures for assessing safety 55
7.6 Criteria for stopping treatment 58
7.7 Procedures at the end of the trial 60
7.8 Sub study: particpants and community related perceptions 60

8 Safety Reporting 62
8.1 Definitions and Classification 62
8.2 Adverse events that require expedited reporting by the centres 64

9 Statistical Considerations 67
9.1 Method of randomisation 67
9.2 Outcome measures 67
9.3 Sample size 68
9.4 Interim monitoring and analyses 69
9.5 Data analyses and presentations 69

10 Data Management 72

10.1 Data management at the Clinical Centre 72
10.2 Data management in the immunology laboratories 74
10.3 Data management at the Central Data Management Centre 74

11 Trial Monitoring 75
11.1 Continuous and Internal Study Monitoring 75
11.2 TMG and safety calls during the study 75
11.3 Clinical site monitoring 75
11.4 Monitoring by the Trial Management Group 76
11.5 Confidentiality 76
11.6 Quality Assurance and Quality Control of Data 76

12 Ethical Considerations and Approval 78
12.1 Ethical issues 78
12.2 Ethical Considerations 78

13 Indemnity 80
13.1 Insurance for investigators 80
13.2 Health care Insurance for trial volunteers 80

14 Finance 81
14.1 Compensation for study participation 81

15 Trial Committees 82
15.1 Trial Management Team (TMT) 82
15.2 Trial Management Group (TMG) 82
15.3 TaMoVac Steering Committee 83
15.4 Community Advisory Board (CAB) 83
15.5 Data and Safety Monitoring Board (DSMB) 84

16 Publication 85

17 Protocol Amendments 86

18 References 87

19 Apendices 91

List of Tables

Table 1: Schedule of doses, formulation and routes of immunisation (short) 17
Table 2: Solicited adverse events .... 18 & 57
Table 3: Mozambique HIV prevalence rate estimate 22
Table 4: Schedule of doses, formulation and routes of immunisation (long) 46
Table 5: Source Documents 74

LIST OF FIGURES

Figure 1: Flow diagram 21

APPENDICES

APPENDIX 1: STUDY TIMETABLE 90
APPENDIX 2: TAMOVAC II SCHEDULE OF EVENTS & LAB FLOW CHART.......................... 92
APPENDIX 3A1: TAMOVAC II INFORMATION SHEET (ENGLISH) TANZANIA..................... 94
APPENDIX 3A2: TAMOVAC II INFORMATION SHEET (ENGLISH) MOZAMBIQUE 94
APPENDIX 3B: TAMOVAC II INFORMATION SHEET (SWAHILI).... 95
APPENDIX 3C: TAMOVAC II INFORMATION SHEET (PORTUGUESE) 95
APPENDIX 4A: TAMOVAC II ASSESSMENT OF UNDERSTANDING (ENGLISH) 95
APPENDIX 4B: TAMOVAC II ASSESSMENT OF UNDERSTANDING (SWAHILI) 95
APPENDIX 4C: TAMOVAC II ASSESSMENT OF UNDERSTANDING (PORTUGUESE)............ 95
APPENDIX 5A: TAMOVAC II RISK ASSESSMENT OF VOLUNTEERS (ENGLISH) 95
APPENDIX 5B: TAMOVAC II RISK ASSESSMENT OF VOLUNTEERS (SWAHILI) 95
APPENDIX 5C: TAMOVAC II RISK ASSESSMENT OF VOLUNTEERS (PORTUGUESE) 95
APPENDIX 6A: TAMOVAC II DIARY CARD (ENGLISH) 95
APPENDIX 6B: TAMOVAC II DIARY CARD (SWAHILI).. 96
APPENDIX 6C: TAMOVAC II DIARY CARD (PORTUGUESE)... 96
APPENDIX 7A: TAMOVAC II CERTIFICATE ON HIV VACCINE TRIAL PARTICIPATION (ENGLISH).. 96
APPENDIX 7B: TAMOVAC II CERTIFICATE ON HIV VACCINE TRIAL PARTICIPATION (SWAHILI).. 96
APPENDIX 7C: TAMOVAC II CERTIFICATE ON HIV VACCINE TRIAL PARTICIPATION (PORTUGUESE)... 96
APPENDIX 8: DAIDS TABLE FOR CLINICAL AND LAB CRITERIA 97
APPENDIX 9: ADDITIONAL BACKGROUND INFORMATION 111
APPENDIX 10: PROTOCOL VERSION 2.0 LISTING OF CHANGES 117

ABBREVIATIONS AND GLOSSARY

Ab	Antibody
ADCC	Antibody-dependent cell-mediated cytotoxicity
AE	Adverse event
AIDS	Acquired immunodeficiency syndrome
ALT/AST	Alanine aminotranferase and aspartate aminotransferase liver enzymes
ALVAC-HIV	Canary pox Avipox vectored HIV vaccine candidate (ALVAC-HIV)
APC	Antigen-presenting cells
C	Centigrade or Celsius
CBC	Complete blood count
CD4+	A functional subclass of T cells, helper T lymphocytes (Th) that are necessary for augmentation and coordination of innate and adaptive effector responses, humoral and cellular
CD8+	Cytotoxic T-Cells that destroy host cells, which have become infected by viruses or other intracellular pathogens
CDC	United States Centers for Disease Control and Prevention
CEFC	Chick embryo fibroblast cells
CRC	Clinical Research Coordinator
GMP	Good Manufacturing Practices
CIOMS	Council of International Organization of Medical Sciences
CISPOC	Centro de Investigação e Treino em Saúde de Polana Caniço
CRF	Case report form
CRP	C Reactive Protein
CTL	Cytotoxic T lymphocyte
DAERS	DAIDS Adverse Experience Reporting System
DAIDS	U.S. Division of AIDS, NIAID, -
DCAC	Data Coordinating and Analysis Center
DNA	Deoxyribonucleic acid
DSMB	Data and Safety Monitoring Board
ECG	Electrocardiogram
EDTA	Ethylenediaminotetraacetic acid
EAE	Expedited Adverse Events
ELISA	Enzyme linked immunosorbent assay
ELISPOT	Enzyme-Linked Immunospot
EP	Electroporation
Env	Envelope
FDA	U.S. Food and Drug Administration
FTA	Fluorescent treponemal antibody
GCP	Good Clinical Practices
GLP	Good Laboratory Practices
HBsAg	Hepatitis B surface antigen
HCM	Hospital Central de Maputo
HIV and HIV-1	Human immunodeficiency virus, type 1
HLA	Human leukocyte antigen
HVTN	HIV Vaccine Trials Network
IAVI	International AIDS Vaccine Initiative
IB	Investigator's Brochure
IC	Imperial College of London
ICF	Informed Consent Form
ICH	International Conference on Harmonization
ICS	Intracellular cytokine staining
ID	Intradermal injection
IgG	Immunoglobulin G
IM	Intramuscular injection
IND	Investigational New Drug Application

INF-γ	Interferon gamma
INS	Instituto Nacional de Saúde
IRB	Institutional Review Board
LMU	Ludwig-Maximillians University of Munich
LPA	Lymphocyte proliferation assay
LSI	Lymphocyte stimulation index
MH	Muhimbili Hospital
MHC	Major histocompatibility complex
MHRP	US Military HIV Research Program
mg	Milligram
mL	Milliliter
mm	Millimeter
MMRC	NIMR-Mbeya Medical Research Center
MNH	Muhimbili National Hospital
MO	Medical Officer
MRC CTU	Medical Research Council Clinical Trial Unit
MRH	Mbeya Referral Hospital
MUHAS	Muhimbili University of Health and Allied Sciences
MVA-CMDR	Modified Vaccinia Ankara- Chiang Mai Double Recombinant
NAb	Neutralizing antibody
NIAID	National Institute of Allergy and Infectious Diseases
NIMR	National Institute for Medical Research
NYVAC	New York Vaccinia Virus Strain
PBMC	Peripheral blood mononuclear cells
PBS	Phosphate buffered saline
PCR	Polymerase chain reaction
pfu	Plaque forming units (equivalent to International Units)
PI	Principal investigator
PIN	participant identification number
PIR	post-injection reactogenicity
rMVA	Recombinant MVA
RNA	Ribonucleic acid
RPR	Rapid plasma reagin
SADR	Suspected adverse drug reaction
SAE	Serious adverse event
SC	Subcutaneous injection
SIV	Simian immunodeficiency virus
SMI	Swedish Medical Institute
SOE	Schedule of Evaluations
STEP	HVTN 502/Merck 023 trial
SUSAR	Suspected unexpected serious adverse reaction
TaMoVac	Tanzania Mozambique Vaccine Trial
TB	Tuberculosis
TBD	To be determined
TFDA	Tanzania Food and Drug Administration
TM01	TaMoVac 01 Trial
TM02	TaMoVac 02 Trial
TMG	Trial Management Group
TMT	Trial Mananagment Team at each clinical centre
UKHVC	UK HIV Vaccine Consortium
UNAIDS	The Joint United Nations Programme on HIV/AIDS
U.S.	United States
VRC	Vaccine Research Centre of the U.S. INS
VDRL	Venereal Disease Research Laboratory
WB	Western blot

WBC	White blood cell count
WHO	World Health Organization
WRAIR	Walter Reed Army Institute of Research

1 SUMMARY

1.1 Summary of trial design

1.1.1 Design

This is a randomised, three-centre, factorial design to assess a prophylactic HIV vaccine with the HIVIS DNA prime administered ID by the Zetajet® device with or without the Derma Vax™ electroporation device at a dose of 0.6mg in a concentration of 3mg/ml or in a concentration of 6mg/ml, followed by IM MVA-CMDR 10^8pfu per ml either alone or given at the same time as IM 100 μg CN54 rgp140 adjuvanted with 5μg GLA-AF.

Approximately 18 participants will be allocated to receive placebo. Clinical staff will not know who received placebo, but they cannot be blinded to the group, and will know whether the participant is allocated to electroporation, and receives one or two injections at each timepoint. Laboratory staff will be blind to the group and the allocation of placebo.

Disease/participants studied

198 healthy, HIV uninfected volunteers, 18 to 40 years old who are at low risk of HIV infection will be recruited with 80 from Mbeya and 80 from Dar es Salaam, Tanzania and 38 from Maputo, Mozambique. A minimum of 50 females will be enrolled, and they will be evenly distributed between the three groups.

1.1.2 Trial interventions

The doses, formulations, routes and schedule of immunisation are described in Table 1 below.

Table 1 Schedule of doses, formulation and routes of immunisation

Groups		DNA prime at weeks 0, 4, 12	MVA+/- CN54 rgp140/GLA-AFboost at weeks 24 and 40
I	**A** Vaccine (n=60)	**A** 2 inj. of 0.1mL DNA tot 600µg	**A.1:** 1x1 mL MVA 10^8 pfu IM and 1x0.4 mL [100 µg] CN54 rgp140/GLA-AF [5µg] IM (n=30)<br>**A.2:** 1x1 mL MVA 10^8 pfu IM (n=30)
	**B** Placebo (n=6)	**B** 2 inj. of 0.1mL saline ID Zetajet®	**B.1:** 1x1 mL saline IM and 1x0.4 mL saline IM (n=3)<br>**B.2:** 1x1mL saline IM (n=3)
II	**A** Vaccine (n=60)	**A** 2 inj. of 0.1mL DNA tot 600µg	**A.1:** 1x1 mL MVA 10^8 pfu IM and 1x0.4 mL [100 µg] CN54 rgp140/GLA-AF [5µg] IM (n=30)<br>**A.2:** 1x1 mL MVA 10^8 pfu IM (n=30)
	**B** Placebo (n=6)	**B** 2 inj. of 0.1mL saline ID Zetajet® +Derma Vax™ EP	**B.1:** 1x1 mL saline IM and 1x0.4 mL saline IM (n=3)<br>**B.2**: 1x1mL saline IM (n=3)
III	**A** Vaccine (n=60)	**A** 1 inj. of 0.1mL DNA tot 600µg	**A.1:** 1x1 mL MVA 10^8 pfu IM and 1x0.4 mL [100 µg] CN54 rgp140/GLA-AF [5µg] IM (n=30)<br>**A.2:** 1x1 mL MVA 10^8 pfu IM (n=30)
	**B** Placebo (n=6)	**B** 1 inj. of 0.1mL saline ID Zetajet® +Derma Vax™ EP	**B.1:** 1x1 mL saline IM and 1x0.4 mL saline IM (n=3)<br>**B.2:** 1x1ml saline IM (n=3)

DNA will be given in **combined** plasmid pools: 1 injection ID of 0.1 ml of 3mg/ml the combined Pool 1 (env) and Pool 2 (gag) into the left and right arm (Groups I and II) or only into the left arm (Group III), 0.1 ml of 6 mg/ml.

1.1.3 Objectives

Primary objectives:

1. To compare the safety and immunogenicity of three different priming regimens of 600µg HIVIS-DNA administered to healthy HIV uninfected volunteers as:

    a. Two 300µg injections (3mg/ml) ID via Zetajet® without Electroporation or
    b. Two 300µg injections (3mg/ml) ID via Zetajet® followed by ID Derma Vax™ Electroporation or
    c. A single 600µg injection (6mg/ml) via Zetajet® followed by ID Derma Vax™ Electroporation

2. To compare the safety and immunogenicity of boosting healthy HIV uninfected adult participants primed with HIVIS DNA with:

    a. IM MVA-CMDR with IM 100µg CN54 rgp140 adjuvanted with 5µg GLA-AF or

    b. IM MVA-CMDR alone

Secondary objectives:

1. To build expertise and capacity for the evaluation of HIV-1 vaccine candidates in Tanzania and Mozambique

2. To evaluate the perception, attitude and knowledge towards the TaMoVac vaccine trials within study participants and their social environment

1.1.4 Outcome measures

Safety

- Grade 3 or above local solicited adverse event **(Table 2)**
- Grade 3 or above systemic clinical and laboratory solicited adverse event **(Table 2)**
- Any grade of adverse event that results in a clinical decision to discontinue further immunisations
- Any grade of adverse event that occurs in a participant that has received at least one immunisation

Table 2 Solicited adverse events

Local AEs<br>(immunisation site)	Local Pain<br>Local Itching<br>Warmth<br>Redness in cm<br>Swelling (soft) in cm<br>Induration (hard) in cm<br>Blisters (blood or clear) in cm<br>Papule in cm
Systemic Clinical AEs	Temperature<br>Chills<br>Myalgia/flu-like general muscle aches<br>Aching joints<br>Malaise (excess fatigue)<br>Headache<br>Nausea<br>Vomiting
Systemic Laboratory AEs	Creatinine, ALT, total and direct bilirubin, glucose<br>Hb, total WBC, neutrophils, lymphocytes, platelets

Immunogenicity

- The presence and breadth of IFN-γ ELISpot responses to a pool of HIV-peptides encoded by the vaccine to which there was no response at baseline.
- The magnitude of IFN-γELISpot responses as measured by the number of spot forming cells per million PBMCs in response to pools of HIV-peptides in the IFN-γElispot assay.
- The presence of CD4+ and CD8+ T-cell cytokine responses to pools of HIV peptides assessed by ICS.
- The presence and endpoint titre of systemic CN54rgp140-specific IgG antibodies.
- The presence and endpoint titre of systemic IgG and IgA HIV-specific binding antibodies.
- The presence and endpoint titre of tier 1 and tier 2 neutralising antibodies.

1.1.5 Duration

The enrolment period is planned to be 10 months. Study participants will be monitored through week 12 (optionally week 24) after the last injection resulting into a total study duration after enrolment of 64 weeks for each participant.

1.1.6 Data recorded directly on case report forms (CRFs)

Data will be recorded directly onto the case report forms, which will provide the majority of source data for the trial or entered onto a source document checklist and transcribed to the appropriate case report form later. There will be some additional source data in the clinical notes, such as medical history related to eligibility, results of laboratory analyses and details of clinical management (description of adverse events and concomitant medication). The site research coordinator and internal monitors will crosscheck the completeness of the CRFs or the correctness of data transcription to the CRFs from source documents at the clinic. The original copies of all CRFs and source documents will be kept at the clinic in the volunteers/participants study binder, which is placed in a secured and lockable locker at the study site. The documents in the binder will be organized according to study number and study visit. Any changes to the CRFs should be signed and dated.

1.1.7 Organisation

The Swedish Institute for Communicable Disease Control (SMI) will act as the Sponsor in collaboration with The Muhimbili University of Health and Allied Sciences (MUHAS) for the TaMoVac II study. The trial is funded by the European and Developing Countries Clinical Trials Partnership Programme (EDCTP).

Involved Tanzanian institutions are the Muhimbili University of Health and Allied Sciences (MUHAS), Muhimbili National Hospital (MNH), National Institute for Medical Research (NIMR), NIMR-Mbeya Medical Research Center (MMRC) and the Tanzania Police Force.

Involved Mozambican institutions are the Instituto Nacional de Saúde (INS), INS-Centro de Investigação e Treino em Saúde de Polana Caniço (CISPOC) and the Hospital Central de Maputo (HCM).

European institutions are the Swedish Institute for Communicable Disease Control (SMI) and the Karolinska Institute (KI) in Stockholm in Sweden; the University of Munich (LMU) in Germany; and the Medical Research Council Clinical Trials Unit (MRC CTU) in London, United Kingdom.

The US collaborating institute is the Walter Reed Army Institute of Research (WRAIR), U.S. Military HIV Research Program (MHRP) in Rockville, Maryland.

The clinical sites will be MNH/MUHAS in Dar es Salaam and NIMR-Mbeya Medical Research Centre (MMRC) in Mbeya, Tanzania and CISPOC in Maputo Mozambique

Participating laboratories:

1. The Department of Microbiology and Immunology, MUHAS, Dar es Salaam.

2. NIMR-Mbeya MMRC, Mbeya.
3. The immunology laboratory of the Instituto Nacional de Saúde,Hospital Central de Maputo (HCM) and the CISPOC laboratories, Mozambique
4. The Swedish Institute for Communicable Disease Control, Stockholm, SWEDEN.
5. The Walter Reed Army Institute of Research (WRAIR), USA

1.1.8 Flow Diagram
Screening, randomisation and immunisations

Screen1 wk-8 to -4
Screen2 wk-4 to -2
Enrol/Randomise wk0

IA	IB	wk	IIA	IIB	wk	IIIA	IIIB
DNA* Zetajet®	Saline*Zetajet®	0	DNA* Zetajet® Derma Vax™ EP	Saline* Zetajet® Derma Vax™ EP	0	DNA** Zetajet® Derma Vax™ EP	Saline** Zetajet® Derma Vax™ EP
DNA*Zetajet®	Saline*Zetajet®	4	DNA* Zetajet® Derma Vax™ EP	Saline* Zetajet® Derma Vax™ EP	4	DNA** Zetajet® Derma Vax™ EP	Saline** Zetajet® Derma Vax™ EP
DNA*Zetajet®	Saline*Zetajet®	12	DNA* Zetajet®Derma Vax™ EP	Saline* Zetajet®Derma Vax™ EP	12	DNA** Zetajet®Derma Vax™ EP	Saline** Zetajet®Derma Vax™ EP
MVA10^8 pfu + CN54 rgp140/ GLA-AF MVA 10^8 pfu	Saline # Saline ##	24	MVA 10^8 pfu + CN54rgp 140/ GLA-AF MVA 10^8 pfu	Saline # Saline ##	24	MVA 10^8 pfu + CN54 rgp140/ GLA-AF MVA 10^8 pfu	Saline# Saline# #
MVA10^8 pfu + CN54 rgp140/ GLA-AF MVA 10^8 pfu	Saline # Saline ##	40	MVA 10^8 pfu + CN54 rgp140/ GLA-AF MVA 10^8 pfu	Saline # Saline ##	40	MVA 10^8 pfu + CN54 rgp140/ GLA-AF MVA 10^8 pfu	Saline# Saline# #

* 2 injections of 0.1mL with 3mg/mL DNA (600µg) or 2 injections of 0.1mL saline ID
** 1 injection of 0.1mL with 6mg/mL DNA (600µg) or 0.1mL saline ID
\# 1x1mL saline and 1x0.4mL saline IM
\## 1x1mL saline

2 BACKGROUND

2.1 Background

2.1.1 The need for a vaccine

The global AIDS epidemic continues to grow. In its *2009 Report on the global AIDS epidemic*, UNAIDS published the following estimates for 2008:[1] UNAID report 2009 http://http://wwwunaids.org/en/default/asp

- 33.4 million people were living with HIV
- There were 7,400 new infections per day
- Women were disproportionately susceptible to infection within sub-Saharan Africa, acquiring 60% of the infections
- Gains in expanding access to HIV treatment cannot be sustained without a reduction in the rate of new HIV infections

The International AIDS Vaccine Initiative (IAVI) has estimated that a vaccine has the potential to prevent over 70 million infections in 15 years (1).

2.1.2 HIV in Tanzania

The 2007-08 Tanzania HIV/AIDS and Malaria Indicator Survey (THMIS) found that 5.8% of the population age 15-49 in Tanzania were HIV/AIDS positive with prevalence higher among women than men (7% and 5%, respectively)(2). Among blood donors at Muhimbili National Hospital (MNH) the prevalence of HIV was 8.7% (3). In the Mbeya region, by 1995 the prevalence had increased substantially in all antenatal centers to 11.9% overall with higher prevalence in urban sites (17%). In addition to data in pregnant women, MMRC collected population-based HIV prevalence data during the recruitment of its study populations (cohorts). Data from the CODE Study (unpublished data) indicated HIV prevalence rate of 19.6% in the general urban population (18-45 years) and 14.4% in the general rural population. Overall, HIV prevalence data from Mbeya reveals a slightly higher HIV prevalence in women compared to men, especially in the younger age groups (20-30 years) with also the highest incidence rates for young females. Studies conducted in Dar es Salaam, Mbeya and Kilimanjaro have shown that the major circulating HIV-1 subtypes in Tanzania are clades A, C, D and their recombinant forms (4-7).

2.1.3 HIV in Mozambique

The first case of HIV/AIDS in Mozambique was diagnosed in 1986, where shortly after a national HIV program to battle the epidemic was established. The first HIV surveillance done in a population of pregnant women in 2002, showed an HIV prevalence of 13.6%. In 2007, the HIV prevalence in the same group was estimated at 16.2% and in 2009 15%. Regional differences are shown in Table 3.

Table 3: Mozambique HIV prevalence rate estimate (pregnant women (15-49 years) attending antenatal care by region, 2002-2009)

.

Region	2002	2004	2007	2009
South	16% (12-18%)	19% (14-21%)	21% (16-23%)	21% (17-25%)
Centre	18% (17-20%)	19% (17-21%)	18% (17-12%)	18% (14-21%)
North	8% (6-9%)	9% (14-16%)	9% (7-10%)	9% (7-11%)
**NATIONAL**	**15% (13-15%)**	**16% (14-16%)**	**16% (14-17%)**	**15% (14-17%)**

Source: MOH/INS, 2009

In 2009, a national population based survey (INSIDA) was organized and showed an estimated of 11.5% HIV prevalence in adults (15 - 49 years). Regional differences are notable with 5.6%, 12.5% and 17.8% for North, Center and South of Mozambique, respectively. A worrying fact shown from the INSIDA study is the difference in HIV estimate rates between young girls and boys (15-24 years): girls are highly affected at a younger age (11.1% 15-24 years) whereas boys keep a lower HIV prevalence in the same age group (3.7% 15-24 years) (8). Studies conducted in Mozambique show that the main circulating HIV-1 subtypes in Mozambique are clade C and their circulating recombinant forms.

2.1.4 The immune response to HIV

The study of experimental and natural infections suggest that both HIV-specific neutralising/functional antibodies and long lasting effector and central memory HIV-specific CD8+ and CD4+ T-cell responses are needed both in systemic and mucosal compartments to effectively control HIV infection (9-16). The recent results of the RV144 trial have further endorsed a vaccine centred on the development of broad, potent and durable cellular and humoral immune responses.

2.1.5 The status of the field

The first clinical trials of candidate HIV vaccines started over 20 years ago and despite sustained effort and four efficacy trials, there had been virtually no good news until 2009 when RV144 - the "Thai trial" reported modest protection in a cohort of low risk, predominantly heterosexual individuals (17). RV144 was a community-based, randomised, multicentre, double-blind, placebo-controlled trial consisting of four "priming" injections of a recombinant canarypox vector (ALVAC-HIV expressing *gagpro* and *env* genes) followed by two "boosting" injections with vaccine AIDSVAX B/E Env protein given together with the last two injections of ALVAC in ALUM. In the modified intention-to-treat analysis involving 16,395 subjects, the vaccine efficacy in terms of preventing acquisition of HIV was 31.2% (95% CI, 1.1-52.1; p=0.04). Vaccination did not affect viraemia or the CD4+ T-cell count in those who subsequently became infected (see Appendix 9 for more detail). Levels of two distinct types of serum antibody have been shown to correlate with the risk of infection: IgG against the V1/V2 loop of the viral envelope was negatively correlated with risk of infection whilst levels of monomeric serum IgA with the same specificity were positively correlated with risk of infection (18). The relatively modest effect size was seen as a major step forward in vaccine research, providing the first suggestion that the development of a safe and effective preventive HIV vaccine was possible. Interestingly, both ALVAC and AIDSVAX failed to show efficacy in previous trials and the only novel component of the trial design was the fact that the two were combined for the last two immunisations endorsing such so-called "combination strategies".

2.1.6 Current vaccination strategies

There have always been strong proponents of vaccines focussed on either B-cell or T-cell immune responses and the history of vaccine research has reflected this. RV144 has endorsed a vaccine strategy based on the development of a more balanced immune response and efforts are now also focussed on increasing the potency and quality of adaptive immune responses which are present concurrently with, if not before HIV-1 transmission occurs (19,20). Many groups are exploring multicomponent heterologous prime-boost vaccine regimens which include an adjuvanted envelope glycoprotein.

2.1.7 Heterologous prime boost regimens

Heterologous prime boost regimens involve priming the immune system to (a) target antigen(s) delivered by one vaccine and then selectively boosting the response by repeated administration of the antigen(s) by a second, distinct vaccine (21). Studies in non-human

primates showed that priming with DNA followed by boosting with modified viruses such as Adenovirus (Ad5) or recombinant MVA could reduce challenge virus replication and thus also prevent the development of SIV-induced disease (22-25). Several groups are in the process of optimising heterologous prime-boost regimens for use in humans using a variety of DNA plasmids and modified viral vectors. The strategy has proved particularly potent for T-cell responses – with more mixed success seen in stimulating antibody responses (26-32). In line with the renewed focus in the B-cell response to envelope glycoproteins, several groups now plan to incorporate adjuvanted envelope proteins into existing prime boost regimens and, as discussed above, there is a particular interest in the role of non-neutralising antibodies with specificity for the V1/V2 loop of the viral envelope.

2.1.8 Methods to increase the efficiency of DNA priming

After encouraging results in animals, DNA vaccines have proved relatively disappointing in human studies and there has been a focus on improving delivery to target cells.

2.1.8.1 Zetajet® Needle-Free device

By expelling contents under pressure through at high velocity, needle free injection devices have been shown to disperse plasmids to a wide area in the tissue and also to target the antigen-presenting cells (APC) in skin. Our group has previously used the Biojector® 2000 in a number of HIV vaccine studies. This device is driven by pressurised CO2 and has a 1mL syringe which makes it difficult to dispense the small volumes (0.1ml) typically administered ID. It has been superceded by the spring loaded Zetajet which uses a 0.5mL syringe. The Zetajet is being used in the current TaMoVac I study in Mozambique and in the HIVIS 07 study in Sweden. In the HIVIS 07 the Zetajet is being used in combination with ID electroporation. The Zetajet is thought to offer a more standardized inoculation and, being needle free, removes the risk of exposure to unsterilized needles. The most important advantage of the ID Zetajet is the ID delivery of DNA in a controlled manner. Biojector delivery has been shown to enhance the antibody response to Hepatitis A vaccine in humans (33) and to HIV DNA vaccines in prime/boost strategy with MVA in mice (34). Importantly, there is no increased risk of integration (35-36). Recent studies evaluating use for administration of the injectable antiretroviral medication enfurvirtide showed decreased injection site reactions and increased acceptability compared to standard needles (37). The IM or ID use Biojector® 2000 administration as used in recent HIV vaccine studies in Tanzania (RV172, HIVIS03, ongoing TaMoVac I) have shown excellent safety data and acceptability. The Zetajet device has been well tolerated delivering 0.1 or 0.2 mL intradermally in the HIVIS 07 and TaMoVac I trials in Stockholm and Maputo respectively.

2.1.8.2 Electroporation (EP)

Electroporation (EP) increases the transfection efficiency into antigen-presenting cells (APCs) by creating transient pores in the cell membranes increasing the uptake of DNA. To date there is no evidence of increased risk of integration of plasmid DNA into the genome in mice – even using high doses of DNA (38,39). The resultant (mild) tissue damage may actually serve to recruit APCs thereby accelerating the initiation of an inflammatory response (40). The ID Derma Vax™ DNA Vaccine Skin Delivery EP System has been used in preclinical studies in combination with needleless ID injections and found to induce greatly enhanced immune responses. Based on these studies it is now used in clinical studies of DNA vaccines against colon cancer and HIV (see below). The ID Derma Vax™ device delivers a series of electric pulses over 0.3 sec between two arrays of 2 mm long needles to the superficial skin usually over the deltoid. Skin resistance is registered for every pulse. It has so far been very well tolerated in ongoing human studies. Several human Phase I/II clinical trials exploring the use of EP devices from various manufacturers are currently being conducted to evaluate the safety and immunogenicity of this method. The first clinical trial to use EP for the delivery of a HIV DNA vaccine recently reported an encouraging augmentation of immunogenicity without any increased safety concerns paving the way for further larger studies (41). A phase I HIV study (HIVIS07, ISRCTN60284968) started in

November 2010 in Sweden in order to assess the safety and immunogenicity of the HIVIS DNA administered using the ID Derma Vax™ DNA Vaccine Skin Delivery EP System followed by MVA boost in HIV-uninfected adults. The study is investigating the same vaccine candidates and Electroporation device as proposed for use in TaMoVac II and safety and tolerability data will directly inform the design.The Dermavax device has been well tolerated in the ongoing HIVIS 07 in Sweden where the same DNA volume/delivery and procedure as proposed for this study is employed.

2.2 Work leading to this trial:

2.2.1 HIVIS trials

The HIVIS trials conducted in Sweden and Tanzania employed the same multiclade DNA and MVA vaccine candidates proposed for use in TM02. The trials have followed the same basic design following a vaccination regimen consisting of 3 priming injections with the 7 DNA plasmids given in two separate pools at weeks 0, 4 and 12 followed by 2 boosts with MVA-CMDR. The vaccination schedules modes/concentrations of DNA delivery and precise timings of the MVA boosts varied across the studies, but one of the main objectives was the optimisation of DNA priming. Taken together the results confirmed these vaccine candidates to be potent T and B-cell immunogens. For example, in HIVIS03, which compared the modes of delivery and doses of DNA, healthy volunteers in Dar es Salaam were vaccinated 3 times with 1mg DNA ID using the Biojector device or 3.8 mg IM and then boosted twice with MVA-CMDR. 100% of those vaccinated made γ-IFN Elispot responses to gag and 89% to env after the first boost with MVA given 6 months after the DNA. Higher response rates were seen in the ID DNA primed arms as compared to IM. 90% made gp160-specific antibodies after the second MVA boost which was given 48 weeks after the first MVA boost. (27).

2.2.2 TaMoVac 01

The TaMoVac I study (PACTR2010050002122368), funded by EDCTP and sponsored by MUHAS/SMI, is a randomized, placebo controlled double-blind phase II study. The regimen follows the same basic design as preceding trials and consists of priming 3 times with HIVIS DNA given ID by Biojector at weeks 0, 4 and 12 followed by 2x IM MVA-CMDR at weeks 30 and 46. The trial is focussed on optimization of DNA priming and explores the impact of (i) reducing the quantity of DNA (400μg versus 1000μg) and (ii) the impact of pooling the plasmid pools encoding env/rev and gag/pol peptides. Data from murine studies suggests that balanced ENV and GAG-specific immune responses are only generated when plasmid pools are kept separate and injected into distinct arms but this is obviously not ideal for larger studies. The trial is being carried out in two sites in Tanzania and healthy volunteers have been randomised to receive 400μg DNA plasmids pools encoding (env/rev) or (gag/pol) peptides, injected either as two separate pools injected into different arms or together in a combined pool. A third group receives 1.0mg DNA ID delivered with 600µg (env/rev) and 400µg (gag/pol) in separate pools.

Immunizations were completed at both sites in January 2012. Unblinded cellular immune response results did not show major differences between the separate versus combined plasmid pools for the DNA low dose groups as well as between the pooled DNA low dose groups versus the DNA high dose group in terms of proportion and magnitude of IFNg ELISPOT responses against env and gag for all peptide pools two weeks after the first and second MVA boost. These results provide the basic justification for the decision to proceed with 600µg of DNA in the TaMoVac II trial and to explore electroporation as a means of enhancing immune responses to DNA.

An amendment to the TaMoVac I protocol was submitted in order to add 2x IM CN54rgp140 adjuvanted with GLA-AF boosts given after completion of the DNA/MVA vaccine regimen.

This amendment was possible through collaboration with the AfrEVac consortium. The rationale of the extended vaccine regimen came up after results from the RV144 "Thai Trial" were published. Additionally, through collaboration with the AfreVacc MRC-CTU and the UK HIV Vaccine Consortium (UKHVC) the HIVIS/TaMoVac projects have broadened their networking and trial management capacities as well as options to extend lab capacity building in Tanzania for binding and neutralizing antibody assays.

At the new trial site in Mozambique, a subsequent trial of 24 volunteers was initiated in September 2011. In that study Zetajet is used to deliver two injections of 0.1 or 0.2mL to deliver 600μg DNA or 1200μg DNA prime respectively in order to get further indications if a higher DNA priming dose will result in better immune response after boosting with 2 MVA injections in a shorter schedule 3 months apart. The immunogens are the same as in TaMoVac I and II.

Furthermore, both the HIVIS-03 and TaMoVac I trials have enhanced the human, infrastructural, and regulatory capacity for the conduct of HIV vaccine trials in Tanzania and Mozambique as well as the community responsiveness towards such trials.

2.3 Rationale for design and objectives

A variety of strategies have been employed to enhance the efficiency of DNA priming including addition of adjuvants such as cytokines and methods to improve delivery including needle free devices such as the Biojector and Zetajet® and/or electroporation using devices such as the ID Derma Vax™.

The primary aim of the first randomisation is to assess the impact of electroporation (by Derma Vax™) used in combination with the needle free Zetajet® for DNA delivery after priming three times with 600μg DNA in a concentration of 3mg/ml (2 shots) or 600 μg DNA in a concentration of 6mg/ml (1 shot) followed by boosting twice with MVA-CMDR. The safety and immunogenicity of each of these regimens will be compared to 600 μg DNA in a concentration of 3mg/ml (2 shots) administered with the needle free Zetajet®, but without electroporation, a schedule that was employed in TaMoVac 01, and which was not significantly less immunogenic than the higher dose of 1000μg DNA. Administration of the DNA vaccine in a higher concentration (6mg/ml) as compared to the 3mg/ml concentration applied in TaMoVac 01 will reduce the overall number of shots in order to deliver the vaccine. An overall lower DNA dose priming as well as a reduction of shots for vaccine administration would make the DNA vaccine and delivery more feasibility and economic.

Within sub-studies knowledge and perception towards HIV vaccine trials in the recruitment communities will be assessed. Recruitment and acceptance for HIV vaccine trials are largely dependent on the knowledge, perceptions and attitudes towards HIV vaccine development within the volunteers' communities. Following experiences of HIV Vaccine trials in Mbeya (RV 172 and TaMoVac I) and Dar es Salaam (HIVIS-03 and TaMoVac I) members of the communities have misconceptions about HIV Vaccine trials thereby posing challenges towards recruitment and retention of trial participants. As these trials aim to eventually lead to efficacy studies being conducted within the communities, assessing community knowledge, perceptions and recommendations for strategies that will effectively engage volunteers in HIV Vaccine trials will help to establish trustful relations with trial researchers, minimizing misconceptions and consequently improving recruitment and retention of trial volunteers.

The rationale for the second randomisation to explore the MVA-CMDR boost with and without CN54 rgp140/GLA-AF boost is two fold:
Firstly, to build on the promising results on good safety and tolerability generated in this network in 40 Tanzanian participants from the TaMoVac I study, who were boosted with

the CN54 rgp140/GLA-AF following heterologous multiclade priming with DNA and an earlier boost with MVA. Local and systemic reactogenicity was mostly mild, with 4 participants reporting moderate pain or warmth after injection and 3 patients reporting moderate malaise or headache/joint/muscle ache during the 7 days post immunization. One grade 3 neutropenia probably not related to the immunogen was observed. The antibody titres were considerably boosted compared to those seen after the second MVA boost, and preliminary results suggest that the cellular responses are also boosted.
Secondly, to build on the RV144 results in which partial protection was seen following a combination regimen with a vector prime and vector+protein boost given simultaneously. Several groups are planning combinations of 2 immunogens, but we are in a unique position to generate timely data on the combination of DNA, and a simultaneous boost with the MVA vector, and adjuvanted protein (CN54 rgp140/GLA-AF) which we expect to set a new threshold for immunogenicity.

2.3.1 Hypotheses to be explored

Preclinical studies indicate that ID electroporation increases the efficiency of DNA priming. Although intramuscular electroporation is proven in humans effective it is hampered by discomfort. ID electroporation appears to be well tolerated and may thus be suitable for use in large trials in developing countries. Our first hypothesis is that electroporation will increase the efficiency of DNA priming in terms of immune responses and will lead to a dose sparing DNA vaccine regimen. Furthermore increased DNA vaccine concentration will reduce the number of shots necessary to deliver the full dose with the hypothesis to induce comparable immune responses as with lower DNA vaccine concentrations. Our second hypothesis is that simultaneous administration of MVA with CN54rgp140/GLA-AF after priming with DNA will result in significantly enhanced immune responses without compromising safety relative to MVA alone.

2.4 Investigational products/intervention(s)

2.4.1 DNA

The DNA vaccine immunogens are encoded in plasmids derived from puC8 which contains genes for kanamycin resistance and containing the promoter sequence from CMV, the poly (A) signal from HPV-16 and the *E.coli* origin of replication. The plasmids cover subtypes A, B and C but there is evidence for extensive cross-reactivity at the level of both humoral and T cell responses in the recognition of HIV-1 Env and gag subtypes, broadening the geographical scope of such a multiclade vaccine (41), (42), (43). The 7 plasmids are pKCMV**gp160**A, pKCMV**gp160**B, pKCMV**gp160**C, pKCMV**rev**B, pKCMV**p37**A (AB), pKCMV**p37**B, and pKCMV**RT**B.

The primary envelope gene is that of subtype B. This gene was used for homologous recombination to obtain variable sites of subtypes A and C. Thus the most variable regions of gp120 from subtypes other than the primary could be included, and the most conserved proteins conserved in the vaccine mixture.

Rev of subtype B is included to augment the expression of the envelope genes. The vaccine also includes a plasmid encoding **RT** of subtype B, but does not include **pol**.

The pKCMVgp160B/Aand pKCMV gp160B/C encode chimeric gp160B proteins with the hypervariable loops (V1-V5) exchanged for subtype A or C sequences, respectively.

The p37 gag subtypes A and B constructs encode the antigenically most relevant p17 matrix and p24 capsid proteins. p24 is the main vaccine target of the gag proteins and the exchange of p17 subtype A for the subtype B p17 will increase the response to conserved epitopes. The seven plasmids, expressing ten different HIV-1 genes, have previously been

injected at two distinct anatomical sites; with one pool containing the p37 (ABA and B) and RT genes and the other the gp160 (A, B and C) and rev genes. This was in order to mimimise the chances of immunodominance causing interference between the immune responses to different components (44).

2.4.2 MVA-CMDR Modified Vaccinia Ankara (MVA-CMDR)

MVA-CMDR is a recombinant attenuated modified vaccinia virus-vectored vaccine that has been genetically engineered to express HIV-1 gp150 (Subtype E, isolate CM235) and gag and pol (integrase-deleted and reverse transcriptase nonfunctional, Subtype A, isolate CM240) developed at the Laboratory of Viral Disease (LVD), that does not replicate in mammalian cells, National Institute of Allergy and Infectious Diseases (NIAID) by Bernard Moss and Patricia Earl. The vaccine preparation is formulated to contain 10^8 plaque forming units (pfu)/mL in sterile PBS (without Ca^{2+} and Mg^{2+}), 7.5% lactose, pH 7.4. The vaccine is filled in 2 mL glass vials in a volume of 1.2 mL (10^8 pfu/mL) or 0.4 mL (10^9 pfu/mL). The vials are stoppered with grey butyl rubber stoppers and sealed with aluminum crimp seals. Storage of the MVA-CMDR vaccine is at -80°C (± 10°C). The use of live attenuated Modified Vaccinia Ankara virus recombinants as expression vectors for heterologous HIV gene inserts has been shown in clinical trials to be safe and immunogenic (45-46). In particular, Modified Vaccinia Ankara (MVA)-based recombinants have been shown to induce antibodies and specific cellular immune responses mediated by CTLs. The Walter Reed Army Institute of Research/National Institutes of Health (WRAIR/INS) have produced a live recombinant poxvirus vector, MVA-CMDR, that is genetically engineered to express the following HIV genes: gp150 (Subtype E, CM235), gag, and pol (integrase-deleted and reverse transcriptase nonfunctional, Subtype A, CM240). This live attenuated vector vaccine candidate has been shown to be safe and immunogenic in two Phase I protocols, either administered alone (RV158 US, Thailand, WRAIR Protocol # 1143) or when used in a DNA prime/MVA boost immunisation strategy (47) including results from our own group from the phase I HIVIS01/02 study conducted in Sweden (48) and the phase II HIVIS03 study conducted in Dar es Salaam.

Several preclinical studies to evaluate the bio distribution of DNA vaccines (42) and MVA (Investigator's brochure, Edition 6, 6th December 2012) were performed in Sweden and in the United States and showed that DNA plasmids and MVA are quickly eliminated from different organs including the gonads. In further clinical trials of HIV vaccines conducted in Tanzania, using the same combination of vaccines, no adverse events were recorded in children of vaccinated mothers who became pregnant during the course of the study. However, in order to minimise the risk to the unborn child, all participants will be advised to avoid pregnancy (or pregnancy in partners) until 3 months after the last vaccination and contraception will be available at no cost. Verbal assurance is sought at the time of randomisation to confirm that pregnancy will be avoided until 3 months after the last vaccination.

2.4.3 CN54rgp140/GLA-AF

CN54rgp140: this is a recombinant C-clade Env protein, derived from a Chinese viral isolate. The C-clade HIV subtype is believed to cause more than 50% of worldwide HIV-1 infections, and is predominant in southern and eastern Africa and India. It comprises a sequence of 670 amino acids, and has been shown to be immunogenic in non-human primates and other animal models. CN54rgp140 has been manufactured (GMP) using a mammalian cell expression system by Polymun and purchased by Imperial College London.

GLA-AF: GLA is a synthetic lipid A adjuvant formulated in aqueous solution, manufactured to US GMP by IDRI and purchased by Imperial College. GLA-AF is an aqueous adjuvant formulation containing glucopyranosyl lipid A, a completely synthetic monophosphoryl lipid A (MPL®) like molecule. MPL is an adjuvant component of human vaccines licensed

worldwide. Both GLA and MPL adjuvants are potent stimulators of the innate immune system, specifically mobilizing antigen presenting cells through their binding and activation of the toll-like receptor 4 (TLR4).

To date, there have been three human clinical trials of the recombinant protein CN54 rgp140, known as MucoVac 1 (EudraCT number 2007-000781-20), MucoVac 2 (EudrafCT number) and the TaMoVac 01 v4.0 amendment. The MucoVac trials were carried out at two sites in the UK (London and York). Fifty three healthy women have received three immunizations with CN54rgp140 adjuvanted with GLA-AF via one or more of the following routes: intramuscular, intranasal and intravaginal. Adverse events were mainly mild, and there were no serious or severe adverse events related to the vaccine products.

Further data supporting the safety of CN54 rgp140 and GLA-AF comes from similar recombinant envelope proteins and MPL respectively. Recombinant HIV envelope proteins have been extensively studied in large scale efficacy trials including the VaxGen and RV144 trials in which over 10,000 individuals have been exposed. MPL is an active ingredient in GlaxoSmithKline's propriety adjuvant AS04, which is in Cervarix, a licensed vaccine to prevent certain types of human papilloma virus infection associated with cervical cancer. Cervarix is licensed for use in the European Union and contains 50µg MPL. Pre-clinical studies suggest that the potency of GLA-AF is 10 fold greater than MPL, which would imply that the equivalent dose to 50µg MPL is 5µg GLA-AF. GLA has also been formulated in oil and water emulsion (GLA-SE) and this has been used in one previous clinical trial. Four of the first cohort of 8 volunteers received one IM immunisation with fluzone plus GLA-SE 5µg formulated in the emulsion. Three of the four experienced multiple grade 2 or higher adverse events, mainly local reactions but also headache and malaise, considered to be dose limiting toxicity and attributed to the emulsion formulation, so the dose ranging study in 84 individuals proceeded with a maximum dose of 2.5µg GLA-SE. None of the reported events in the 54 individuals that received up to 2.5µg GLA-SE reached dose limiting toxicity, and the proportion with grade 2 or higher events was similar in those receiving GLA-SE to those receiving emulsion without GLA: 30% and 25% respectively. There was one severe event considered related to vaccine in the GLA-SE groups, a headache that started on the day of immunization lasting 24 hours occurring in a participant that received 2.5µg GLA-SE. Priming the immune system prior to administration of GLA-AF did not alter the adverse event profile of CN54 rgp140/GLA-AF in TaMoVac 01. This was expected because GLA-AF does not stimulate adaptive immune responses that could lead to a cascade of cytokines and enhanced reactogenicity.

2.4.4 Vaccine Delivery

2.4.4.1 Zetajet® Needle-Free Injection System

The Zetajet® uses sterile, single-use syringes that deliver the study material ID which is self powered by a spring. The syringes will be prepared by the pharmacist and delivered to the clinic for vaccination upon request (see section 6).The syringes will be loaded into the Zetajet by the trained pharmacists, study doctor or nurse according to manufacturer's instructions. The Zetajet will be applied on the disinfected skin with light pressure. The study agent is expelled under pressure through a micro-orifice at high velocity in a fraction of a second.The Zetajet® device will be provided by Bioject Medical Technologies, Inc., 20245 S.W. 95th Avenue, Tualatin, Oregon 97062.

2.4.4.2 Derma Vax™ DNA Vaccine Skin Delivery EP

The Derma Vax™ DNA Electroporation System is a device that delivers a tightly controlled and documented series of electrical pulses to the skin between two arrays of 2 mm long needles 4mm apart (see section 6).

2.4.4.3 MVA-CMDR

The CMDR-MVA/Placebo boost injection will be delivered IM in the deltoid muscle of the left arm according to standardised operational procedures.

2.4.4.4 CN54rgp140/GLA-AF

100μg CN54rgp140 will be formulated with 5μg GLA-AF in aqueous solution by the pharmacist in the 2 hours prior to use and administered IM into the deltoid muscle of the right arm.

2.4.5 Rationale for doses

The rationale for dose selection is based on previious HIVIS trials and the TaMoVac I trial. Results from the TaMoVac I did not show major differences between DNA vaccine deliveries of separate versus combined plasmid pools as well as between the higher and lower DNA doses. Therefore, the TaMoVac II will further investigate the lower TaMoVac I DNA dose (600 μg) with combined plasmid pools in a clinically more feasible (two shots) and less costly, dose sparing DNA vaccine concept (Group I). In addition the effect of electroporation will be investigated for the 600μg DNA dose (Group II) with the hypothesis to induce higher DNA vaccine efficacy in terms of immunological correlates. Furthermore, the options to decrease immunisation shots necessary to deliver the full dose will be evaluated by comparing administration of the DNA vaccine in one shot at a higher concentration of 6 mg/ml (Group III) as compared to previously used 2 shots at a concentration of 3 mg/ml as applied in Groups I and II. The CMDR-MVA and the CN54rgp140/GLA-AF doses are adapted from the previous HIVIS and TaMoVac I trials.

2.4.6 Risks and benefits

The DNA and MVA immunogens have been well tolerated in the HIVIS 01, HIVIS 03 and TaMoVac I studies and have now been given to a total of ~260 participants in several settings. Injection with Zetajet is associated with the same level of discomfort as traditional injections. Electroporation with the ID Derma Vax™ device is associated with a momentary unpleasant contraction of superficial muscles, but very little pain. The effect subsides immediately. There is no indication that the increased delivery of DNA is associated with integration that could be clinically relevant. Bloodletting can be unpleasant and result in bruises that resolve within days. Participation in a study offers regular medical checkups and intense counselling to avoid sexually transmitted infections and pregnancies.
Results from the study may contribute significantly to the development of an HIV vaccine.

→ ***Additional background information on HIV immune responses, HIV vaccine developments and vaccine delivery are provided in Appendix 9.***

3 SELECTION OF CENTRES/CLINICIANS

3.1.1 Muhimbili University of Health and Allied Sciences (MUHAS)

MUHAS has established in collaboration with the MNH a dedicated HIV clinic that offers comprehensive HIV care. Additionally, a dedicated HIV vaccine clinical trial unit is in place within MNH premises that is closely linked with the Department of Microbiology/Immunology at MUHAS. This allows for an ethical conduct of HIV vaccine trials by providing an experienced study team, community outreach team, pharmacy expertise, data management capabilities, as well as competent laboratories. The HIV vaccine clinic has built up experience and capacities throughout the conduct of the HIVIS03 trial and the ongoing TaMoVac I study. The clinical and laboratory study team is GCP and GCLP trained and repeat training is performed every other year. Throughout these studies good relationships have been established to a cohort among Police Officers which serves as the basis for the recruitment. Extended community involvement into the general population as a source of recruitment has been established throughout the TaMoVac I. The clinical and laboratory study team is GCP/GCLP trained and repeated training is performed every other year. Operators performing the ELISpot assay have received training and passed an operators validation test which involves running three known samples with low, medium and high IFN-gamma ELISpot reactivity to a peptide pool on three different occasions.

3.1.2 NIMR-Mbeya Medical Research Center (MMRC)

MMRC had been involved in past HIV vaccine and cohort trials and has established an experienced study clinic, community outreach team, pharmacy and laboratories. MMRC has built up trial experience throughout numerous past and ongoing HIV cohort (HISIS, CODE, RV217) and HIV vaccine trials (RV172, TaMoVac I and RV262). Clinical support is provided throughout close collaboration with the Mbeya Referral Hospital (MRH). The MMRC labs include a clinical laboratory (haematology, clinical chemistry, serology, CD4 count analysis, HIV testing and HIV RNA) as well as an immunological lab which has established FACScanto flow cytometry, ICS and ELISPOT assays throughout various studies. Both labs have been accredited by the College of American Pathologists (CAP) and participate in external QA/QC programs with UKNequas and CAP and follow therefore the highest standard of quality control and validation which is audited and monitored yearly by independent consultants. The MMRC labs have been recognized as one of the national reference laboratories. The clinical and laboratory study team are GCP/GCLP trained and repeat training is performed every other year.

3.1.3 Centro de Investigação e Treino em Saúde da Polana Caniço (CISPOC), Instituto Nacional de Saúde (INS) in Maputo.

The laboratory at the INS has been improved in terms of needed reagents and equipment to enable screening and safety evaluation (HIV serology, biochemical tests, as well as haematological tests) of potential volunteers as well as monitoring of immune responses following vaccination (Interferon-gamma ELISPOT assay). Local reference values were determined for T-lymphocyte subsets, haematological and biochemical parameters using samples from healthy youths. A new research clinic has been built and equipped to accommodate the TaMoVac trials and other research projects. Personnel have been trained in GLP and GCP and taken a pivotal role in adapting the TaMoVac protocol to local conditions.

Since the end of 2009, a cohort of youths (N=1380) attendees at Hospital Central de Maputo (HCM) have been recruited to assess its suitability for future HIV vaccine trials by determining the prevalence and incidence of sexually transmitted viral infections. Mozambique has a cohort of youths available for HIV vaccine trials. The Youth Clinic at HCM has an established team that works with Youths, and will serve as a base for the cohort of the proposed trial. In preliminary workshops around 200 youths have expressed a general

interest in vaccines. A subset have declared an explicit interest in HIV vaccine trials and been invited to more detailed workshops on HIV vaccines. Those that then express an interest to themselves participate in the planned trial are invited to the clinic for individual information, counselling and eligibility checks. In addition, individuals who attended the Youth Clinic for general services including HIV counselling and testing, STI screening and health education will also be invited to participate in the trial.

4 SELECTION OF PARTICIPANTS

There will be **no exceptions** to eligibility requirements at the time of randomisation. Questions about eligibility should be addressed prior to attempting to randomise the participant.

The eligibility criteria for this trial have been carefully considered. The eligibility criteria are the standards used to ensure that only appropriate participants are considered for this study. Participants not meeting the criteria should not join the study. For the safety of the participants it is important that no exceptions be made to these criteria for admission to the study.

Participants will be considered eligible for enrolment in this trial if they fulfil all the inclusion criteria and none of the exclusion criteria as defined below.

4.1 Participant inclusion criteria

1. Age: 18 to 40 years
2. Willing to undergo counselling and HIV testing
3. Have a negative antigen/antibody ELISA for HIV infection
4. Able to give informed consent
5. Basic abilities to read and write
6. Satisfactory completion of an assessment of understanding prior to enrolment defined as 90% correct answers after three opportunities to take test
7. Resident in Dar es Salaam, Mbeya or Maputo catchment areas, and willing to remain so for the duration of the study
8. At low risk of HIV infection, defined as the absence of an identifiable risk factor/ behaviour according to a risk assessment questionniare. The following criteria will be considered high risk leading to exclusion:
   - sexual partner with HIV
   - sexual partner with unknown HIV serostatus who is also unwilling to use protective condoms consistently in all sexual relations
   - sexual partner is known to be at high risk for HIV
   - more than two sexual partners in the last 6 months
   - history of being an alcoholic [as medically defined or more than 35 units /week] or intravenous drug abuse or ongoing other chronic drug abuse (e.g. marijuana)
   - history of STI within past 6 months
9. Verbal assurances that adequate birth control measures are used not to conceive/father a child during the study and up to 3 months afterthe last vaccine injection. Adequate birth control is defined as follows:
   - Contraceptive medications delivered orally, intramuscularly, vaginally, or implanted underneath the skin
   - Surgical methods (hysterectomy or bilateral tubal ligation)
   - Condoms, diaphragms
   - Intrauterine device (IUD)
   - Abstinence
10. Women shall have a negative urinary pregnancy test
11. Be willing to practice safe sex for the duration of the study to avoid sexually transmitted infections including HIV

12. Good health as determined by medical history, physical examination, clinical judgment and by key laboratory parameters as judged by the study physician
13. Laboratory criteria within 8 weeks prior to enrollment:
    - Hb >10.5 g/dl
    - White blood cell count >2000 to <13,000 cells/mm^3
    - Neutrophils >1,100 cells /mm^3
    - Lymphocytes >1.0 cells/mm^3
    - Platelets >125,000/ mm^3
    - Random Blood Glucose <6.44 mmol/L; if elevated, then a Fasting Blood Glucose <6.11 mmol/l
    - Bilirubin <1.1 x uln
    - ALT <1.25 x uln
    - Creatinine <1.1 x uln
    - Urine dipstick for protein and blood: negative or trace. Glucose negative.

4.2 Participant exclusion criteria

The following are the criteria for exclusion from the study:

1. At risk of HIV infection as mentioned above in the inclusion criteria
2. Active tuberculosis or other systemic infectious process elicited by review of systems, physical examination and laboratory detection (for example detection of Hepatitis B surface antigen or active syphilis)
3. A history of immunodeficiency, ongoing medical and/or psychiatric condition and/or chronic illness requiring continuous or frequent medical intervention
4. Autoimmune disease by history and physical examination
5. Hives or recurrent hives and severe eczema
6. Substance abuse problems (including traditional medicine) during the past 12 months that in the opinion of the investigator would preclude participation
7. History of grand-mal epilepsy, or currently taking anti-epileptics
8. Received blood or blood products or immunoglobulins in the past 3 months.
9. Receiving immunosuppressive therapy such as systemic corticosteroids or cancer chemotherapy
10. Use of experimental therapeutic agents within 30 days of study entry
11. Reception of any live, attenuated vaccine within 60 days of study entry. Medically indicated subunit or killed vaccines (e.g., Hepatitis A or Hepatitis B) are not exclusionary but should be given at least 2 weeks before or after HIV immunisation to avoid potential confusion of adverse reactions.
12. History of cardiac disease including:
    - Previous myocardial infarction (heart attack)
    - angina pectoris; congestive heart failure
    - valvular heart disease
    - cardiomyopathy
    - myo/pericarditis
    - stroke or transient ischemic attack
    - chest pain or shortness of breath at rest or with activity (such as walking up stairs)
    - arrhythmia/episodic palpitations (not excluded: sinus arrhythmia)
    - pacemaker or
    - other heart conditions under the care of a doctor

13. Abnormality in ECG that could indicate risk or make interpretation of vaccine effects difficult according to the study operating procedures. ECG with clinically significant findings, or features that would interfere with the assessment of myo/pericarditis, including any of the following:
    - conduction disturbance (complete left or complete right bundle branch block or nonspecific intraventricular conduction disturbance with QRS >120 ms, an AV block grade II or III, or QTc prolongation [> 440ms])
    - significant repolarisation (ST segment or T wave) abnormality
    - significant atrial or ventricular arrhythmia; frequent atrial or ventricular ectopy (e.g., frequent premature atrial contractions, 2 premature ventricular contractions in a row)
    - ST elevation consistent with ischemia; or evidence of past or evolving myocardial infarction
14. Previously received an HIV candidate vaccine.
15. History of severe local or general reaction to vaccination defined as:
    - **Local**: Extensive, indurated redness and swelling involving most of the major circumference of the arm, not resolving within 72 hours
    - **General**: Fever >= 39.5 ^{0}C within 48 hours; anaphylaxis; bronchospasm; laryngeal oedema; collapse; convulsions or encephalopathy within 72 hours
16. A positive pregnancy test or breastfeeding at screening or enrolment.
17. Study site employees who are involved in the protocol and may have direct access to the immunogenicity results
18. Unlikely to comply with protocol as judged by the principal investigator or his designate.

4.3 Number and source of participants

4.3.1 Recruitment in Dar es Salaam

A total of 80 volunteers will be recruited from the Dar es Salaam site. They will come from a number of sources, including Police Officers, Prison's officers, and the general public. Police Officers' (PO's) volunteers will be recruited with the assistance of an already established "core group" of about 400 PO's who will assist with educational activities on HIV/AIDS and HIV vaccine studies in the force. The same procedures as in HIVIS-03 and TaMoVac-01 studies will be used, as these have proven useful in avoiding coercion and social discrimination. Meetings at the Police stations, efforts by the "core group" of collaborators and a one to one contact, will be the main modalities to reach potential volunteers. Also volunteers from the Prison force Officers in Dar es Salaam will be addressed for recruitment, first experiences and contacts have been made throughout the TaMoVac I recruitment process. Volunteers will also be sought from the general public including young adults attending the Infectious Disease Clinic (IDC) who would have been assessed by the clinical team regarding their availability for long-term follow up. Information and briefing sessions will be provided after review by the institutional ethics committee to all interested and invited volunteers that will contain general information on HIV and AIDS, HIV Vaccines, and HIV vaccine clinical trials through power point presentations, information sheets, as well as the opportunity for individual questions and counselling. Additionally, volunteers in the ended HIVIS-03 and TaMoVac-01 studies will be asked to assist with recruitment from their respective communities.

4.3.2 Recruitment in Mbeya

Volunteers will be recruited from the Mbeya urban environment through advertisement using poster, newspaper advertisement, flyers and local radio. MMRC has developed over the years throughout the past cohort studies a well-established relationship to the

communities. Community leaders and community advisory boards of various areas in the Mbeya region will help in the advertising and recruiting process. Recruitment of the required number of female participants had not been a problem throughout the past study recruitment procedures (RV172, TaMoVac I and RV262). Interested volunteers will be invited to an information and briefing session. These sessions will contain general information through power point presentations about the study. Information sheets as well as the opportunity for individual questions and counselling will be provided. There will also be time for questions on an individual basis without ties to the particular group involved. Interested volunteers will receive an appointment for the first screening visit or contact details for later screening assignment. Information sessions will also be provided for the community leaders and the community advisory board. The briefing and information sessions after review by the institutional ethics committee will take place at the MMRC vaccine trial location in Mbeya and at designated meeting places in communities.

4.3.3 Recruitment in Maputo

Volunteers will be recruited at the Youth Clinic at HCM. The established Youth Clinic will be used to assist with educational activities on HIV/AIDS and HIV vaccine studies as well as recruitment. Meetings at HCM Youth Clinic, efforts by the "core group" of collaborators and a one to one contact, are the main modalities to reach potential volunteers. The interest in participating in the trial will be expressed individually and anonymously. Information and briefing sessions containing general information through power point presentations about the study, information sheets as well as the opportunity for individual questions and counselling will be offered before and during the trial. Information sessions will also be provided for the community leaders and the community advisory board. The briefing and information sessions after review by the institutional ethics committee will take place at the Youth Clinic at HCM and all study visits at CISPOC. The evaluation of substance abuse will be made through anamnesis (by the study doctor) and through the use of the risk assessment form (Appendix 5). The use of corticosteroids in any medical condition is an exclusion criterion for participation in this study. Volunteers with a history of asthma in the childhood/ adolescence that do not show acute episodes (in adulthood) may be included in the study depending on the clinical evaluation by the study doctors. Volunteers with recurrent asthmatic bronchoconstriction will be carefully evaluated by the medical team to ensure that volunteers who may potentially require treatment with glucocorticoids are not included in the study. If necessary, a pulmonologist can be consulted for a final decision.

4.4 Screening procedures and pre-randomisation investigations

At the screening visit the volunteer will be allocated a number from the register. The trial will be discussed in detail, and a check of eligibility conducted using a case report form to standardise this procedure. Any questions about the study will be answered. If volunteers are still eligible, willing and interested they will be asked to sign the informed consent form (***Appendix 3***).

To ensure informed consent, volunteers will go through the following processes in detail with a trained member of the study team:

1) The rational of the vaccine trial
2) Study timelines, visit schedules and availabilities
3) The concept of blinding and randomization
4) Pre-HIV risk assessment & discussion
5) Safe sex counselling. High quality hypoallergenic condoms will be provided free of charge to volunteers throughout the trial.
6) That it is unknown whether or not the study vaccines will protect against HIV infection
7) That repeated HIV tests will be performed

8) That following immunisation they may develop antibodies that will produce a positive reaction in a routine HIV test, but that provisions have been made to distinguish between a post vaccination response and HIV infection during and after the trial.
9) The level of care that will be made available to them should they be found to be HIV infected at any time during their participation in the study, including the screening period.
10) That they, or their partner should continue to use a reliable form of contraception for 14 days prior to the immunisation period and for 3 months afterwards
11) That they should have no high risk partners and continue to use condoms with sexual partners whose HIV status is not known
12) That they may be subject to social risk if they develop HIV antibodies, or by revealing their participation in the study
13) The risks and benefits to participate in the trial.
14) Risk associated to the study products (vaccines). The concept of safety monitoring and adverse events/reactogenicity reporting will be explained.
15) That blood will be taken for safety and immunological tests. The amount of blood will be indicated. Explanations will be provided that tests will be performed at the study sites or at other specialized laboratories in Europe and the US.
16) That blood tests might include genetic testing. The risks of genetic testing and how to prevent them within the study will be explained.
17) Thatparticipants can withdraw study participation at any time without any disadvantages.
18) Confidentiality and trial surveilance

Upon this information about the trial and the informed consent procedures, consenting volunteers will be requested to successfully pass a test of understanding ***(Appendix 4)*** with at least 90% of correct answers with the possibility of maximum 3 repeated attempts.

Study personnel will assess volunteers for past and current risk of HIV infection using the Risk Assessment Questionnaire ***(Appendix 5)***, followed by counselling for those who will consent to undergo HIV test prior to collecting blood. The counselling process will ensure that volunteers have sufficient knowledge about HIV infection to understand what the test is for, the implications of a positive, negative and equivocal result and the standard of care available for HIV infection locally. They will also be informed on how and when they will receive the result.

After written informed consent has been collected, assessments and investigations will be undertaken according to the schedule in ***Appendix 2***. These include demographic, sexual and medical histories, general examination, ECG and collection of urine and blood samples for routine laboratory investigations. Screening investigations for sexually transmitted infections including HIV will be collected ***(see section 7.2*)**.

As soon as the medical report is received and all required test results are available:

- data will be entered onto the screening CRFs and the results of the screening investigations will be reviewed and eligibility signed off by a physician
- completed screening CRFs for all volunteers, whether or not they are eligible will be entered onto the trial database.

Screening will be performed during two screening visits. Additional screening visits to follow-up on medical conditions might be necessary. If more than 3 months have elapsed since the first screening visit, lab examinations (CBC and chemistry) as well as a medical status examination will have to be repeated. The latest visit should be 2-4 weeks before study visit 3, with an HIV serology no older than 8 weeks.If a volunteer is not found to be eligible a Screening Outcome Form will be filled indicating reasons for ineligibility. A study photo identification card will be produced and provided. Finger print identification will be

used at some sites. The photo ID will be kept by the study participant and will have to be presented at each study clinic contact for identification purposes, unless other unequivocal identification can be documented. For screened out volunteers Study ID will be retained.
For volunteers enrolled in the study, the photo ID card will be collected at the last study visit and will be replaced by the TaMoVac II certificate on HIV vaccine trial participation (Appendix 7).

5 ENROLMENT & RANDOMISATION PROCEDURE

5.1 Enrolment

The Enrolment visit (Study Visit 3) will be counted as Week/Day 0 of the study schedule. During the Enrolment Visit confirmation will be made and recorded that the volunteer fulfils all inclusion/exclusion criteria including laboratory results and eligibility criteria using a checklist. If a volunteer is not found to be eligible a Screening Outcome Form will be filled indicating reasons for ineligibility. Enough time has to be planned for the visit so that all questions from the volunteer about the study can be answered or reflected upon. A senior investigator will confirm if the volunteer understands the concepts of the study and will also assess the willingness of the study volunteer to participate. For enrolment the initial screening number will be used as the study number. This means that study numbers may be discontinuous. The participant will then be randomized into one of the Study Groups.

5.2 Randomisation

There will be two randomization steps. During the first step prior to any vaccination, participants will be randomized according to different DNA immunizations (Groups 1, 2 or 3) and according to vaccine or placebo (A or B). During the second step prior to MVA, vaccination participants will be randomized according MVA (Group A1) or MVA plus gp140/GLA (Group A2) boosting maintaining the placebo affiliation (Groups B1 or B2). Randomisation lists will be drawn up under the supervision of Dr Moshiro. These will be stratified by centre and gender to ensure a balance between the three active groups and matched placebos. The assignment for each subject will be placed in an envelope with a unique sequential number. Six sets of plain envelopes will be prepared, two for each centre (for males and females) and couriered to the Study Pharmacist for that centre who will take the prepared envelopes and put them in a box that is kept under lock.

The pharmacist will randomise participants accepted into the study, after receiving a copy of the participant's signed informed consent from nurse/doctor, by selecting the next consecutive envelope containing the vaccine or placebo assignment in accordance with the generated randomization list. The Pharmacist will make a record of the assignment and study number in a register held under lock. In the case a subject has to be replaced this is noted in the register; their assignement should not be given to the next volunteer to be randomized. The next volunteer should be allocated the next assignment. The Pharmacist will then inform the Study Nurse/Medical Officer at the clinic of the randomization group, I – III of the participant, but will not inform on whether this is vaccine or placebo. The Study Nurse/Medical Officer will record the group assignment in the study record of the participant's file. Procedures for the second randomisation will be similar. In addition to centre and gender, allocation will be stratified on the outcome of the first randomisation.

5.3 Randomisation codes and unblinding

The vaccines will be kept in the department of Microbiology/Immunology at MUHAS, the study pharmacy at MMRC and the pharmacy at CISPOC under appropriate storage conditions according to manufacturer's specifications. The study pharmacist who has access to the envelopes will draw the study vaccine and the placebo into syringes and label them with the study number, the centre, the randomization group, I to III, date and time and hand them over to the study nurse/study doctor for injection. The study nurse/study doctor administering the vaccines, all other site investigators and lab personnel as well as the participants themselves will be blinded to the allocation of vaccine or placebo.

In the unlikely event that an investigator requests unblinding in order to manage an adverse event, the case will be discussed amongst the Trial Management Group. If a consensus to unblind is reached, then the Study Pharmacist will inform the clinician

managing the event of the allocation, and document this on the pharmacy register. Ideally this individual will be independent of the trial team. Laboratory personnel and other members of the trial team will remain blind to the allocation.

5.4 Co-enrolment guidelines

Participants will be advised that they cannot enrol in any other trials during the period from screening to the final visit in the TaMoVac II study. If staff discover that a participant is enrolled on another study during this period, they must immediately contact the Principal Investigator or Clinical Research Coordinator of the study centre for advice. Decisions will be reviewed on a case by case basis by the TaMoVac II Trial Management Group Coordinating Committee (TMG) and participant safety will be the primary concern.

5.5 Replacements in the Enrolment and Randomisation Lists

Study participants who withdraw consent, are lost to follow up or are terminated from the vaccination schedule (e.g. because of pregnancy) should not be replaced in the randomisation list. The next consecutive assignment number on the list should be allocated to the next participant. The randomisation lists at each centre will be long enough to allow this. Whilst enrolment is ongoing, every effort will be made to achieve the planned number of 198 participants completing all five immunisation timepoints, and therefore slightly more than 198 may be enrolled.

6 TREATMENT OF PARTICIPANTS

6.1 Vaccine products

198 participants in sixgroups will receive three DNA or saline injections followed by two MVA-CMDR with or without CN54rgp140 adjuvanted with GLA-AF or saline injections in a schedule described in **Table 4 (section 6.4)**. Each participant will receive a total of five or seven immunisations.

6.2 Administration of vaccines

The immunizations will take place in an outpatient setting. Prior to each immunization participants will receive a baseline medical status check up including vital signs assessment to confirm the medical eligibility before vaccine administration. Safety lab procedures, urine analysis as well as HIV testing will be performed before vaccine administration. A pregnancy test for female participants will have to show negative results before each immunization.

The study pharmacist will draw the vaccines or placebos into the applicable syringes for the Zetajet devices in case of DNA vaccinations or into syringes for IM use in the case of the MVA and CN54 rgp140/GLA-AF vaccinations according to standardized operational procedures. Prior to use the vials will be thawed at room temperature. Thawed vials will then gently be swirled. Care will be taken not to shake or invert the vials.

Upon receipt of the syringes the study nurse/study doctor will deliver the vaccine.
The DNA vaccines/placebo will be administered in one or two injections ID using the needle free Zetajet® device at weeks 0, 4 and 12. The prepared syringes will be loaded into the Zetajet by the study pharmacist according to manufacturer's instructions. The Zetajet will be applied on the disinfected skin with light pressure. The study agent is expelled under pressure through a micro-orifice at high velocity in a fraction of a second. The study product should be administered as soon as possible after preparation. If this is not possible, the syringe may be stored under refrigeration (2 to 8ºC) but the product must be administered within 4 hours after being drawn up into the syringe.

For study groups II and III electroporation using the Derma Vax™ device will be applied immediately after immunization over the injection site according to manufacturer's instructions and SOP. The Derma Vax™ DNA Electroporation System is a device that delivers a tightly controlled and documented series of electrical pulses to the skin between two arrays of 2 mm long needles 4mm apart.

The CMDR-MVA vaccine/placebo boosting will be administered IM using suitable needles and syringes at weeks 24 and 40 into the the deltoid muscle of the left arm. The MVA will be given in a dose of 10^8 pfu infectious doses in 1 ml volumes (groups IA, IIA, IIIA) or 1 ml sterile saline will be injected (groups IB, IIB, IIIB).

The CN54rgp140 has to be mixed with the adjuvant GLA-AF for each of the two immunisations. This will be carried out by the pharmacists at each clinical research centre, under sterile conditions, according to standard operating procedures which are summarised as follows. The preparation will involve drawing 0.3mL of GLA-AF with a 0.5-1mL sterile syringe and transferring it into the cryovial containing 0.3mL of 0.5 mg/mL CN54 rgp140 solution. After gentle mixing of the two solutions, 0.4mL of this mixture will be drawn up using a 0.5–1 mL syringe, for administration. The vials containing the mixed formulation may be stored at room temperature during the period between mixing and administration; the mixture is stable for 2 hours. However, the rgp140/GLA-AF will be mixed just before each immunisation is about to take place, in sterile conditions in the clinic pharmacy and

passed to the study nurse for immediate use. The dose volume will be 0.4mL injected into the deltoid muscle of the right upper arm, using a 21–25 gauge needle long enough to reach deep into the muscle. The date and time of administration will be recorded in the CRF.

After each immunization participants will be closely observed at the clinic for 30 minutes. Vital signs and any local or systemic reaction will be recorded and participants will be seen briefly by the study doctor to document the post vaccination status. The needles, syringes and other materials will be disposed according to MNH/MMRC/CISPOC biohazard regulations. As with any parenteral vaccine, epinephrine and corticosteroids will be available for immediate use should an immediate hypersensitivity reaction, such as anaphylaxis, occur. Intravenous injections of vaccines will NOT be performed. Study vaccine will not be administered to individuals with hypersensitivity to any component of the vaccine. All immediate hypersensitivity reactions will be managed according to the developed SOP's.

Participants will be contacted by phone, home visit or clinical contact at the first day following immunization for a brief adverse reaction interview. Additionally, participants will complete diaries over events for the evening of the vaccination day and the 7 days following each vaccination. A diary card **(*see Appendix 6*)** will be provided after each immunization, with instructions and a full verbal explanation for volunteers to record local and systemic adverse events following immunization, as well as any medication taken. A folder with a diary card, thermometer and a ruler will be issued to each volunteer. The clinical team will provide contact information as well as a telephone number to call 24 hours, in order to assure contact with the study personnel if needed. Furthermore the study clinic will contact participants by phone within 48 hours after each immunization to assure their well being.

All participants will be asked to contact the study site in the case of adverse events of suspected grade 2 or more for evaluation. A decision to offer the partcipants a visit to the clinic should be made and documented in the source documents. All volunteers with suspected grade 3 or more should be seen by the study physician and documented in the source documents.

Safety follow-up visits are scheduled at week 2 and week 4 after each immunization visit. Diary cards will be reviewed and discussed at the scheduled 2 weeks follow-up visit after each vaccination. Diary entries will be transcribed after revision and discussion with the participant during the follow up visits after each vaccination into a Vaccine/Reaction Form.

6.3 Accountability for used and unused supplies

The PI must ensure that all IMP supplies are kept in a secure area accessible only to authorised individuals, and maintained in storage that guarantees the required temperatures.

Supply, storage, composition and labelling of the DNA vaccines

Vecura Company (Karolinska University Hospital at Huddinge, Stockholm, Sweden) will be responsible for bulk manufacture of the DNA vaccine, release testing and technical release of vialled product and labeling. All procedures will be according to Good Manufacturing Practice. The presentation is in liquid form which should be stored at –20 ^{0}C until use. Care will be taken not to break the cold chain.

The DNA plasmids were approved for human use on November 27th 2004 by the Swedish Medical Products Agency, (Svenska Läkemedelsverket), and the second revision of the protocol ethically cleared by the Swedish Internal Review board, (Regionala forskningsetiska kommitten) in December 2004. The new batch of the same plasmids will

need to have a further approval by the Tanzania Food and Drugs Authority (TFDA) and by the Pharmaceutical Department (Regulatory Authority) and Ministry of Health of Mozambique.

DNA will be packaged by Vecura. The trial products will be in vials pre-labeled according to standardized operating procedures. Each vial will be packed in a box labeled with the name of the clinical site and principal investigator, the storage details and the name of the supplier of the product, the name of the manufacturer, as well as the manufacture and expiry dates. The labels should also have the caption "for clinical trial only".
Cartons will be supplied to the designated pharmacist at MUHAS, MMRC and CISPOC for storing the used vials, labeled with the supplier, the name of the trial and protocol version, the name of the clinical site and principal investigator and the content of the material (DNA vaccine or placebo).

Having been manufactured by Vecura Company, the DNA vaccine will be supplied to the study by the Swedish Institute for Communicable Disease Control, 171 82 Solna, SWEDEN.

Sterile commercially available normal saline for human use will be used as the placebo.

Supply, storage, composition and labelling of CMDR MVA
Walter Reed Army Institute of Research (WRAIR), USA, will be responsible for the bulk manufacture of the clinical material, release testing, as well as the technical release of vialled product, and labeling. The full address of the manufacturer is: *Walter Reed Army Institute of Research (WRAIR), Forest Glen Section, Building 501, Department of Biologics Research The Pilot Bioproduction Facility, Silver Spring, MD 20910, USA.* All procedures will be according to Good Manufacturing Practice. The presentation is in liquid form at 10^8 pfu per mL with an extractable volume of 1ml vials, which should be stored at -20 ^{0}C up to 3 months (-80°C if longer than 3 months) until use. Care should be taken not to break the cold chain. The product has already received approval from the USA Food and Drugs Authority (FDA), and the Swedish Medical Products Agency and will be presented to the TFDA for approval before use in Tanzania as well as to Regulatory Authority (Pharmaceutical Department) and Ministry of Health for approval before use in Mozambique.

Sterile commercially available normal saline for human use will be used as the placebo.

Each vial will be packed in a box labeled with the name of the clinical site and principal investigator, the storage details and the name of the supplier of the product, the name of the manufacturer, as well as the manufacture and expiry dates. The labels should also have the caption "for clinical trial only".

Supply, storage, composition and labelling of CN54 rgp140 GLA-AF

CN54 rgp140 has been manufactured GMP by Polymun Scientific, Vienna, Austria. GLA-AF is manufactured and formulated GMP by the Infectious Disease Research Institute, Seattle, USA. The CN54 rgp140 will be supplied at 0.5 mg/mL, and the GLA-AF at 25 µg/mL (0.5mL/vial).

The products should be maintained in storage that guarantees the following temperatures:
- CN54 rgp140 protein at 2-8°C
- GLA-AF at 2-8°C

Conditions for Transport of material

- CN54rgp140 should be shipped at 4°C.
- GLA-AF should be shipped at 4°C.

6.3.1 Measures to ensure cold-chain maintenance and storage

Cold chain will be maintained by transportation packages according to manufacturer's instruction during shipment. This will be documented by temperature registration devices. The packing in Sweden will be divided into different parcels (one parcel for each site) to avoid re-opening the parcel before transporting to Mbeya site in Tanzania. Vaccines will be transported directly to Mozambique. The storage facilities have automatic backup generators to maintain storage temperature. At Dar es Salaam site, transport of the investigational product from the microbiology laboratory to clinic site will be done using study van in a container packed with ice bags. At the MMRC site investigational products will immediately be transferred and stored into the existing freezer at the MMRC pharmacy. At CISPOC the investigational products will immediately be transferred and stored in the existing freezer at the local pharmacy. The study pharmacy and study clinic are located within the same building.

Transportation to the Mbeya Site

The Pharmacist at Dar es Salaam site will be responsible for transporting the investigational products allocated to Mbeya site after being handed to him/her by world courier at Dar es Salaam Airport. Inventory of the consignment for Tanzania will be done by the Pharmacist at the Dar es Salaam site by filling the accountability form. Products allocated to MMRC will be shipped in their original boxes. The products will be stored according to specified storage conditions at MUHAS until the investigational products are transported on dry ice/cool packs to the Mbeya site. Transport to Mbeya will be done as early as possible to avoid delay or change of vaccination schedule for volunteers at the Mbeya site. The pharmacist at Mbeya site will receive the products handled to him/her by filling the handling documents before storing into the respective storage temperatures.

6.3.2 Dispensing records and disposal of unused product

A record must be kept of all DNA, MVA-CMDR, CN54rgp140 and GLA-AF used during the trial. This will include the description (lot numbers and expiry dates) and quantity of IMP received at the trial site and date of receipt, as well as a record of when (date of administration) and to whom (subject number) it was dispensed.

The designated pharmacist will, upon receipt of supplies prior to commencement of the trial, conduct an inventory and complete a receipt, one copy of which will be retained at the site, and the original returned to the supplier (Swedish Institute for Communicable Disease Control, Sweden).

During the trial the pharmacist will be responsible for reviewing the dispensing log. On the day of the vaccination the study nurse/study doctor will fill in the Vaccine Request Form with the study visit number, the study site and participant code, the date as well as the vaccination number and randomized study group. The study pharmacist will check the vaccination number and date against the study number in the dispensing log. He will draw the vaccines or placebos into the applicable syringes for the Zetajet devices in case of DNA vaccinations or into syringes for IM usein the case of the MVA vaccinations according to standardized operational procedures. The study pharmacist will label the syringes with the study number, the site, the randomization group and participant code, date and time and hand them over to the study nurse/study doctor for injection. Labelling and dispensing log procedures will be counter checked by a second person. Upon receipt of the syringes the study nurse/study doctor will deliver the vaccine. The vial label will be cross-checked against the details on the vaccine request form by two individuals at the clinic (e.g. study doctor and study nurse) prior to the administration of the vaccine/placebo.

The Pharmacist will be responsible for ensuring that the used vials are recorded in the dispensing log at the end of the clinical session, and that they are placed in the appropriate

volunteer carton. All supplies (used and unused) will be retained at the trial site until the Sponsor gives instructions for their return/destruction in agreement with the supplier of the vaccines. At the end of the trial all used and unused vials will be checked against the inventory by the designated member of staff responsible for the inventory and the monitor before disposal on site according to MNH/MMRC/CISPOC biohazard regulations. Documentation will be provided to the monitor, Sponsor and supplier. During the trial, product accountability will be monitored by the vaccine request form, the dispensing log, the returns, the trial register and data collected on the clinical research forms.
The Sponsor and the PI will retain copies of the complete IMP accountability records and copies will be provided to the supplier of the vaccines.

6.4 Immunisation schedule

Table 4 Schedule of doses, formulation and routes of immunisation

**Study week**		**0**	**4**	**12**	**24**	**40**
**Injection schedule in days +/ window**		0	28 days (+/-3) post DNA I	84 days (+/- 7) post DNA I	84 days (+/-7) post DNA III	112 days (+/-14) post MVA I
**Study Group**	**N**	**1st vaccination**	**2nd vaccination**	**3rd vaccination**	**4th vaccination**	**5th vaccination**
**IA**	60	**DNA I**<br>*Zetajet®*<br>2x 0.1ml 3mg/ml [300µg] ID of combined Pools (env/gag) in left (1x 0.1 ml) and right (1x 0.1 ml) arm	**DNA II**<br>*Zetajet®*<br>2x 0.1ml 3mg/ml [300µg] ID of combined Pools (env/gag) in left (1x 0.1 ml) and right (1x 0.1 ml) arm	**DNA III**<br>*Zetajet®*<br>2x 0.1ml 3mg/ml [300µg] ID of combined Pools (env/gag) in left (1x 0.1 ml) and right (1x 0.1 ml) arm	**MVA I left arm**<br>1x 1ml of 10^8 pfu MVA IM<br>with (n=30) or without (n=30)<br>**CN54 rgp140GLA-AF right arm**<br>1x 0.4ml [100µg] rgp140/[5µg] GLA-AF	**MVA II left arm**<br>1x 1ml of 10^8 pfu MVA IM<br>with (n=30) or without (n=30)<br>**CN54 rgp140GLA-AF right arm**<br>1x 0.4ml [100µg] rgp140/[5µg] GLA-AF
**IB**	6	**DNA placebo I**<br>*Zetajet®*<br>2x 0.1ml saline ID in left (1x 0.1 ml) and right (1x 0.1 ml) arm	**DNA placebo II**<br>*Zetajet®*<br>2x 0.1ml saline ID in left (1x 0.1 ml) and right (1x 0.1 ml) arm	**DNA placebo III**<br>*Zetajet®*<br>2x 0.1ml saline ID in left (1x 0.1 ml) and right (1x 0.1 ml) arm	**MVA placebo I left arm**<br>1x 1ml of saline IM<br>with (n=3) or without (n=3)<br>**CN54 rgp140GLA-AF placebo right arm**<br>1x 0.4ml of saline	**MVA placebo II left arm**<br>1x 1ml of saline IM<br>with (n=3) or without (n=3)<br>**CN54 rgp140GLA-AF placebo right arm**<br>1x 0.4ml of saline
**IIA**	60	**DNA I**<br>*Zetajet®+DermaVax™EP*<br>2x 0.1ml 3mg/ml [300µg] ID of combined Pools (env/gag) in left (1x 0.1 ml) and right (1x 0.1 ml) arm	**DNA II**<br>*Zetajet®+DermaVax™EP*<br>2x 0.1ml 3mg/ml [300µg] ID of combined Pools (env/gag) in left (1x 0.1 ml) and right (1x 0.1 ml) arm	**DNA III**<br>*Zetajet®+DermaVax™EP*<br>2x 0.1ml 3mg/ml [300µg] ID of combined Pools (env/gag) in left (1x 0.1 ml) and right (1x 0.1 ml) arm	**MVA I left arm**<br>1x 1ml of 10^8 pfu MVA IM<br>with (n=30) or without (n=30)<br>**CN54 rgp140GLA-AF right arm**<br>1x 0.4ml [100µg] rgp140/[5µg] GLA-AF	**MVA II left arm**<br>1x 1ml of 10^8 pfu MVA IM<br>with (n=30) or without (n=30)<br>**CN54 rgp140GLA-AF right arm**<br>1x 0.4ml [100µg] rgp140/[5µg] GLA-AF
**IIB**	6	**DNA placebo I**<br>*Zetajet®+DermaVax™EP*<br>2x 0.1ml saline ID in left (1x 0.1 ml) and right (1x 0.1 ml) arm	**DNA placebo II**<br>*Zetajet®+DermaVax™EP*<br>2x 0.1ml saline ID in left (1x 0.1 ml) and right (1x 0.1 ml) arm	**DNA placebo III**<br>*Zetajet®+DermaVax™EP*<br>2x 0.1ml saline ID in left (1x 0.1 ml) and right (1x 0.1 ml) arm	**MVA placebo I left arm**<br>1x 1ml of saline IM<br>with (n=3) or without (n=3)<br>**CN54 rgp140GLA-AF placebo right arm**<br>1x 0.4ml of saline	**MVA placebo II left arm**<br>1x 1ml of saline IM<br>with (n=3) or without (n=3)<br>**CN54 rgp140GLA-AF placebo right arm**<br>1x 0.4ml of saline
**IIIA**	60	**DNA I**<br>*Zetajet®+DermaVax™EP*<br>1x 0.1ml 6mg/ml [600µg] ID of combined Pools (env/gag) in left arm	**DNA II**<br>*Zetajet®+DermaVax™EP*<br>1x 0.1ml 6mg/ml [600µg] ID of combined Pools (env/gag) in left arm	**DNA III**<br>*Zetajet®+DermaVax™EP*<br>1x 0.1ml 6mg/ml [600µg] ID of combined Pools (env/gag) in left arm	**MVA I left arm**<br>1x 1ml of 10^8 pfu MVA IM<br>with (n=30) or without (n=30)<br>**CN54 rgp140GLA-AF right arm**<br>1x 0.4ml [100µg] rgp140/[5µg] GLA-AF	**MVA II left arm**<br>1x 1ml of 10^8 pfu MVA IM<br>with (n=30) or without (n=30)<br>**CN54 rgp140GLA-AF right arm**<br>1x 0.4ml [100µg] rgp140/[5µg] GLA-AF
**IIIB**	6	**DNA placebo I**<br>*Zetajet®+DermaVax™EP*<br>1x 0.1ml saline ID in left arm	**DNA placebo II**<br>*Zetajet®+DermaVax™EP*<br>1x 0.1ml saline ID in left arm	**DNA placebo III**<br>*Zetajet®+DermaVax™EP*<br>1x 0.1ml saline ID in left arm	**MVA placebo I left arm**<br>1x 1ml of saline IM<br>with (n=3) or without (n=3)<br>**CN54 rgp140 GLA-AF placebo right arm**<br>1x 0.4ml of saline	**MVA placebo II left arm**<br>1x 1ml of saline IM<br>with (n=3) or without (n=3)<br>**CN54 rgp140GLA-AF placebo right arm**<br>1x 0.4ml of saline

6.5 Dose modifications and discontinuation

There are no planned modifications to dose, other than discontinuation.

The schedule may be modified if a participant has symptoms or signs on the day of scheduled immunisation, and the investigator considers it best to defer the immunisation. The participant will be asked to return for review within the window period of the scheduled immunisations. Examples of the type of symptoms and signs are given in **Table 2** (section 7.5.5 of the protocol).

Any discontinuation and reason for early termination of the immunisation schedule will be discussed with the monitors and the TMG and should be reported to the Sponsor and DSMB within the next 2 working days. Trial participants who are discontinued from additional study injections will continue to be followed according to the schedule to further evaluate safety and monitor adverse events. They will also continue to be covered by the medical/trial insurance until the end of the study period. The frequency of laboratory investigations, e.g. for immunogenicity assessments may be reduced on consultation with the monitors and TMG.

6.6 Clinical management of adverse events

The Trial Management Team (TMT) at each study site will assess, manage and treat adverse events as appropriate. Clinical study members include well trained doctors and nurses who are supervised by senior physicians. For adverse events management which exceeds the capacities of the study clinics participants will be referred to appropriate health facilities or independent physicians for further care and treatment at Muhimbili National Hospital (MNH), the Mbeya Referral Hospital (MRH) or Hospital Central de Maputo (HCM). The study clinic will however be responsible for follow up on the medical process of referred participants and will be responsible to report as appropriate as outlined in ***section 8.*** The Trial Management Group (TMG) will meet at regular intervals during conference calls to discuss any relevant medical or adverse events or reactogenicity and will provide support and advice to the study sites.

6.7 Non-trial treatment

Participants will be asked at each visit about medications taken. All medication including other vaccinations (e.g. tetanus toxoid) or herbal/traditional remedies will be recorded in the case record form. Documentation should include the generic name of the drug, indication for use, dose and route of administration, frequency, start and stop dates. For medication available over the counter or other sources, the maximum information available on questioning the volunteer will be recorded. For combination medication it is desirable to include all generic drug names into one entry (e.g. Coartem® = Artemether + Lumefantrine). For herbal or traditional remedies as much information as possible will be sought about the content, the indication or the indigenous name.

The need for the following medications will lead to study discontinuation

- Immunomodulatory agents (i.e. immunoglobulin)
- Immunosuppressive agents (i.e., systemic steroids, chemotherapy)
- Live, attenuated vaccines. Other vaccines (subunit or killed) should be given at least two weeks before or two weeks after HIV immunisation.

6.8 Issues related to HIV

Samples will be tested at the MUHAS Department of Microbiology/Immunology, the MMRC laboratories and the INS Maputo laboratories using HIV antigen/antibody ELISA. A negative first ELISA will be regarded as a negative HIV test result. Positive results will be confirmed by a duplicated second ELISA. In the case of discordant ELISA results, HIV PCR or Immuno Blot analysis will be performed. Positive or indetermined Immuno Blot results will also be confirmed by HIV PCR. A negative Western Blot or PCR result (HIV RNA negative for qualitative PCR tests or HIV RNA <50 copies/µl for ultrasensitive quantitative PCR test) will be interpreted as HIV uninfected indicating a false positive HIV test response due to HIV vaccination. HIV test results after enrolment will be reported as "infected" or "not infected" to the study clinic in order to maintain blinding procedures for vaccine or placebo recipient.

6.8.1 HIV counselling and testing

Only volunteers with a non-reactive antigen/antibody HIV ELISA result will be enrolled. After enrolment HIV testing will be performed before each vaccination and at the long-term follow-up visit at study weeks 52 (Visit 17) and 64 (Visit 18) and whenever a volunteer has the need to establish his/her HIV status. Study participants will receive pre- and post test counselling as well as risk reduction counselling by a trained nurse/counsellor according to country Guidelines. It is possible that participants may develop antibodies and test 'positive' in routine commercial HIV ELISA assays following immunisation. The laboratories at MUHAS, Department of Microbiology/Immunology and MMRC and INS Maputo, will conduct molecular tests (HIV-1 PCR) to distinguish between infection and a post-immunisation response required either for clinical management, or at the request of a volunteer.

During the study, participants will be strongly advised to turn to the study clinic if there is any reason to suspect HIV infection for any reason. Should positive routine serological tests be reported from any other clinic participants are stongly urged to get a second opinion from the study clinic. After study completion participants will be advised to attend the study clinic for any further HIV testing to avoid confusion of false positive test results. After the completion of the trial with consecutive unblinding of the Study Groups, participants will receive an individual unblinding session, indicating the Immunisation Group plus false positive HIV test results. In the event of ongoing post-immunisation positive ELISA, the volunteers will be invited to attend a specified centre until such time as this response has disappeared. All participants will be provided with a Trial Participation Card indicating that they have participated in an HIV Vaccine Trial with the potential of false positive HIV tests due to immunization (***see Appendix 7)***. This card has no expiration date and must be presented by the volunteer whenever he/she desires to have an HIV test.

6.8.2 Management of HIV, Syphilis and Hepatitis B infections during screening and after enrolment

Individuals who test HIV positive during the screening process will be referred for HIV care, treatment and support at the HIV/AIDS clinics at MNH, Mbeya Referral Hospital or Hospital Central de Maputo for clinical care and counselling. These individuals will be followed by an experienced physician on HIV/AIDS and will undergo a full discussion of the clinical aspects of HIV infection and the plans for future care. In the HIV clinics, counseling for HIV/AIDS, clinical and laboratory monitoring, management of symptoms and opportunistic infections and antiretroviral drugs, if indicated, will be offered in accordance with the guidelines of the National Health Service. In Maputo, the CD4+ count will be done at the study site and the result will be forwarded to the HIV/AIDS clinic for follow-up of these individuals.

In Maputo, volunteers with a positive syphilis result will be sent to the youth clinic at HCM for treatment and follow up. The medication for syphilis treatment will be provided by the study. Volunteers infected with hepatitis B virus will be referred for follow-up at the Gastroenterology Service of HCM. Any other participant with a medical condition that requires monitoring and is an exclusion criteria for study participation will be referred for care.

Referral for clinical care
Volunteers will be referred to a study HIV specialist physician at MUHAS/MNH or NIMR-MMRC/Mbeya Referral Hospital or HospitalCentral de Maputo (HCM) for a full discussion of the clinical aspects of HIV infection and planning for further care. Further investigations will be undertaken as necessary and supported by the study clinics. At the HIV clinics sustained counselling about HIV/AIDS, clinical and laboratory monitoring, management of symptoms and opportunistic infections as well as provision of antiretroviral drugs if indicated will be provided.

Referral for counselling
A referral to a counsellor at the HIV clinic at MNH/Mbeya Referral Hospital/Hospital Central de Maputo will be arranged by the specialist physician. The counselling process will assist the volunteer in the following issues:

a. Psychological and social implications of HIV infection
b. Who to inform and what to say
c. Implications for sexual partners
d. Facilitation of risk reduction plan
e. Avoidance of risk to others in the future
f. Information on available medical treatment

Immunological follow-up
Follow-up of HIV infected individuals who have received study vaccine products will be performed according to the study schedule for safety assessment. However, if a participant is found to be HIV-infected during the trial, he/she will be excluded from further immunisations in the trial. The study clinics will work in close collaboration with the HIV clinics providing all relevant clinical and lab information and assisting in CD4 count and viral load monitoring as appropriate. The intensity of assessments will be dependent on clinical progression, including changes in surrogate markers such as CD4 count.

Social discrimination as result of a post-vaccine response
The aim is to minimize the possibility of social discrimination in participants who develop a positive HIV ELISA test by providing HIV testing by PCR and certification for volunteers as required. An identification card stating that the individual has participated in a vaccine trial, with a contact number in case of medical emergency, will be provided.

7 ASSESSMENTS AND FOLLOW-UP

7.1 Duration of follow-up and schedule

An overview on study procedures are provided in the TaMoVac II Schedule ***(Appendix 2)***.

Participants will be required to make a minimum of 17 scheduled outpatient visits over the course of 52 weeks, with an optional extension to 18 visits over 64 weeks. Time 0 will start on the day of enrolment, which is also the day of randomisation and first immunisation.

Screening can take place up to 8 weeks (56 days) before time 0.

Immunisations will take place at weeks 0, 4, 12, 24 and 40.

Clinical safety follow up visits will be conducted 2 and 4 weeks after each immunization.

Long term immunogenicity and safety visits will be performed for a minimum of 12 weeks after the last immunization or are expected to be conducted until 24 weeks after the last immunization (optional).

Serum and peripheral blood mononuclear cells (PBMC) will be collected for immunogenicity at screening (Visit 2), enrolment (Visit 3), and weeks 14 (Visit 9), 26 (Visit 12), 28 (Visit 13), 42 (Visit 15), 44 (Visit 16) and 64 (Visit 18). Optionally the week 64 (Visit 18) immunogenicity tests will be performed at week 52 (Visit 17) if Visit 18 is not expected to be conducted.

Adverse events will be assessed during the enrolment visit following immunisation and at every visit thereafter. Routine laboratory safety parameters will be collected at screening, baseline, at every visit thereafterand at any other unscheduled visit if clinically indicated.

7.1.1 Additional visits

Additional visits and assessments may be required to evaluate an adverse event, and/or identify a diagnosis including additional safety lab tests. These are compatible with the protocol. Referral to an independent specialist with the appropriate expertise will be arranged if there is uncertainty about the relationship to vaccine.

7.1.2 Visit windows

Immunisation visits are compliant with the protocol if they comply with the following target dates and visit windows:

DNA I (Visit 3, week 0): Day 0
DNA II (Visit 5, week 4):28 days (+/-3) post DNA I
DNA III (Visit 8, week 12):84 days (+/- 7) post DNA I
MVA I+/- CN54rgp140/GLA-AF (Visit 11, week 24): 84 days (+/-7) post DNA III
MVA II+/- CN54rgp140/GLA-AF (Visit 14, week 40): 112 days (+/-14) post MVA I+/- CN54 rgp140/GLA-AF

If there is a delay>4 weeks, a decision will be made by the TMG as to whether immunisation will continue.

Safety follow up visits performed 14 days (2 weeks) after each immunization (Visits 4, 6, 9, 12 and 15) will be compliant with the protocol if they take place -3 to +3 days of the target date.

Safety follow up visits performed 28 days (4 weeks) after each immunization (Visits 7, 10, 13, 16) will be compliant with the protocol if they take place -5 to +5 days of the target date.

Long term immunogenicity and safety follow up visits are performed 84 days (12 weeks) and optional 168 days (24 weeks) after the last immunization and will be compliant with the protocol if they take place -14 to +14 days of the target date.

The primary endpoint and follow-up visits are scheduled for 2 and 4 weeks after the second MVA immunisation or at week 42 if immunisations have been discontinued will be compliant with the protocol if they take place ±7 days either side of the target date respectively.

7.1.3 Labeling procedures

Scheduled study visits will be numbered 01, 02, etc. with the addition of 'A-I' for post immunisation assessments for solicited adverse events and also recorded in the diary cards (e.g. 03A, 03B etc.). Unscheduled visits will refer to the last scheduled visit with the addition of 'J-Z' (e.g. 03J, 03K etc.). Site and participant code number will contain 4 digits indicating the first digit 5 for Dar es Salaam, 6 for Mbeya and 8 for Maputo followed by 3 digits for participant identification (e.g. Dar: No. 5001, Mbeya: No. 6001, Maputo 8001). Laboratory samples labels will contain the study visit number, the site and participant code, the date and time of collection, the type of sample (serum, plasma, blood etc.) and a unique sample identification number assigned by the site laboratory.

7.2 Assessments at screening

7.2.1 Demographics, medical history and examination

Demographic information such as age, ethnic origin, smoking, alcohol and illicit drug history will be collected at screening and entered onto the screening CRF.

A past and current medical history will be collected at screening during a face to face structured interview using a case report form, including details of any previous reaction to vaccination, allergies, history of epileptic fit, history of vaccinia (presence of vaccinia scar), reproductive and respiratory symptoms and contraceptive practices (current method and the length of time using the method).

A complete physical examination will be performed. The general examination will include weight (kg), height (cm), temperature, blood pressure, respiratory rate, inspection of the skin to exclude severe eczema and respiratory, cardio-vascular (including ECG, see **section 7.5.3**), abdominal, and neurological examination. An assessment of cervical and axillary lymph nodes will also be undertaken at every visit.

7.2.2 Sexual history and genital infection screen

A sexual history will be taken as part of the HIV Risk Assessment (***see Appendix 5)*** during screening. In addition participants will be asked and examined for signs and symptoms of sexually transmitted infections.

The following will be collected in all participants
- serology for syphilis
- serology for markers of hepatitis B surface antigen

Additional tests (e.g. for Neisseria gonorrhoea, , Trichomonas vaginalis) will be performed as indicated on account of risk and symptoms, according to site specific clinical standardised operating procedures.

7.2.3 Routine laboratory parameters

Blood collections for routine laboratory parameters will be performed according to **Appendix 2.** Samples will be analysed at:

1. The Department of Microbiology and Immunology, MUHAS in Dar es Salaam, Tanzania.
2. The main laboratories of the NIMR-MMRC in Mbeya Tanzania.
3. The laboratories at INS in Maputo, Mozambique

Peripheral blood will be collected by clinical staff experienced in phlebotomy into the appropriate containers and transported to the local laboratory.

Mid-stream urine will be collected into a sterile container and either tested on site or transported to the local laboratory.

The following parameters will be collected in all participants:

- Hb, total WBC, neutrophils, lymphocytes and platelets
- Creatinine, total and direct bilirubin, ALT and glucose
- Urine alysis using a dipstick for protein andblood
- Urine test to exclude pregnancyfor females only

A urine specimen will be sent to the clinical lab for microscopic analysis and validation if abnormal results are seen (≥ +1 for proteins or blood). Results will be graded by a Study Physician as normal, abnormal/clinically insignificant and abnormal/clinically significant.

All lab values will be interpreted according to established MMRC, MUHAS or INS/HCM local reference ranges. Solicited lab values as indicted below for safety assessment will be graded according to the DAIDS Table for Clinical and Lab Criteria for Definitions ***(see Appendix 8). Any grade 3 and 4 lab value needs to be confirmed by a second test within 48 hours.*** In order to harmonize with local reference ranges, the cut-off for Grade I lab toxicity reporting has been modified for neutrophils from ≤1.300 cells/µl (DAIDS) to ≤1.100 cells/µl (local values).

7.3 Other laboratory assessments

7.3.1 Antibodies against Vaccinia

Serum samples will be stored at MUHAS, Department of Microbiology/Immunology, MMRC and INS for later testing.

7.3.2 Immunology

Determination of CD4 counts and percentages only at the enrolment visit is not regarded for safety assessment.

7.3.3 Serology

Syphilis (RPR, and when necessary TPPA) and HBsAg will be performed during screening and if reactive lead to exclusion for study participation. Tests might be repeated during the study if clinically indicated.

7.3.4 Pregnancy test

Pregnancy tests will be performed from urine during screening, at each vaccination day and 3 months after the last vaccination visit (study Visit 17). Urinary pregnancy test results at

immunisation days will be available before vaccine administration. Positive pregnancy results after enrolment will not be reported as an adverse event but in a Pregnancy Report CRF. Pregnant women will be excluded from the immunisation schedule but continued to be followed-up for safety assessments.

7.3.5 Lab analysis following adverse event

Other assessments (e.g. extended clinical chemistry, malaria microscopy, stool analysis, urine culture, TB culture) may be performed as clinically indicated at the sites due to an adverse event. There is the possibility to ship blood samples for specific investigation to specialized laboratories in Europe for further diagnosis.

7.4 Procedures for assessing immunogenicity

Blood collections for immunological tests will be performed according to **Appendix 2.** Samples will be analysed at the:

1. The Department of Microbiology and Immunology, MUHAS, Dar es Salaam.
2. The main and immunological laboratories of the NIMR-Mbeya Medical Research Programme, Mbeya.
3. INS laboratories in Maputo
4. The Swedish Institute for Communicable Disease Control, Department of Immunology and Vaccinology in Stockholm, Sweden.
5. The Walter Reed Army Institute of Research (WRAIR), USA

Analysis will be performed using fresh or frozen samples. Frozen specimens will be prepared, labelled, and stored at the appropriate temperature. Specimens to be shipped will be correctly prepared, labelled, and kept at the correct temperature. A Material Transfer Agreement for samples shipped outside Tanzania or Mozambique will be submitted for approval to the appropriate authorities. All biological samples to be analyzed by the research laboratory will be collected and processed according to institutional guidelines and regulations regarding biobanks.

7.4.1 Cellular responses

The **primary immunogenicity endpoint** will be measured by a standard IFN-γ ELISPOT assay using fresh PBMC and pools of overlapping peptides representing Env, Gag and Pol proteins. ELISPOT responses are considered positive if the numbers of spot-forming cells (SFC) are more than four times the background (RPMI medium only) and > 55 SFC per million PBMC (Merck definition). Quality control and exchange of specimens with international laboratories will be performed.

Other T-cell assays

- CD8 and CD4 T cell responses
  - Multicolor ICS staining using fresh or cryopreserved cells for assessing phenotype and function of vaccine induced T cells. This includes but is not limited to the capacity for production of multiple cytokines/chemokines (IFN-γ, IL-2, TNF, Mip-1β and degranulation (CD107).
  - Other optional assays may include, but are not limited to the lymphocyte proliferation assay (LPA) to the immunizing antigens using a standard ^{3}H-thymidine uptake assay and /or a flow cytometry-based assay. Direct antiviral activity of vaccine induced T cells will be measured with the viral inhibition assay.

7.4.2 Antibody responses

The **primary immunogenicity endpoint** will be measured using standard assays and frozen serum/plasma samples: ELISAs for the detection of binding antibodies and functional assays for the detection of neutralising antibodies. The endpoint titre of binding antibodies with specificity for HIV proteins (Clade A, C and E) and those specific for the immunogen CN54rgp140 will be determined by ELISA.

ELISA absorbance readings for binding antibodies will be classified as 'response' or 'no response' relative to pre-defined cut-offs. Only samples with a detectable antibody response will be subjected to endpoint titration. In cases where a sample is positive for the presence of binding antibodies to CN54rgp140, the absolute concentration of antibody will also be determined. The concentration will be calculated relative to a commercially available standard purified antibody which will be titrated on the same plate as sample sera. Assays for the detection of neutralizing antibodies to HIV subtypes A, C and E will be performed using standardised assays including those using the TZM-bl cell line and PBMCs.

The primary endpoint will be determined from the following results:

- o the endpoint titre and/or concentration of systemic IgG CN54rgp140 specific antibodies measured 2 and/or 4 weeks after the final vaccination
- o the presence of neutralising antibodies to tier 1 and tier 2 viruses and the titre when present

Details of the primary endpoint after the second randomisation will be described more fully in the statistical analysis plan and maybe informed by pending results of the TaMoVac 01 trial.

Other antibody assays

Other assays of antibody phenotype/function may include, but will not be limited to: the duration and avidity of Envelope specific binding antibody responses, the frequency and titre of ADCC responses, the frequency and titre of ADCVI responses, the breadth of Envelope specific antibody responses, the frequency and titre of binding V1V2 specific IgG antibodies (linear and/or scaffolded protein), the extent to which systemic antibodies bind CD4.

Other antibody assays might be carried out from stored samples at collaborating specialized laboratories outside Tanzania and Mozambique which include laboratories in Europe (Prof. Robbin Shattock, Group of Mucosal Infection and Immunity, Department of Medicine, Imperial College, London) and the US (HIV research laboratory of the Military HIV Research Program - MHRP, Laboratory for AIDS Vaccine Research and Development at Duke University). Preferentially, Tanzanian and Mozambican lab scientists are directly involved in assays performed outside Tanzania and Mozambique as part of exchange agreements with collaborating laboratories.

The full panel of laboratory assays performed will be detailed in a TaMoVac II analysis plan approved by the TaMoVac Steering Committee. All samples shipped outside Tanzania will be subject to written Material Transfer Agreements submitted and approved by Tanzanian authorities.

7.4.3 Storage of Cell pellets for HLA testing

Lymphocyte cell pellets will be stored at the MUHAS Department of Microbiology/Immunology, the MMRC Immunological Lab and at INS Labs for later HLA typing which will be performed by molecular methods at a later date if required.

7.5 Procedures for assessing safety

Safety assessments will include solicited local and systemic reactogenicity, adverse events and safety lab assessments.

7.5.1 Adverse event assessment

Information on adverse events will be collected through questions at every visit from and including the enrolment visit as indicated in the schedule of events (**Appendix 2)**. Examples of questions are 'have you had any problems or seen a doctor since your last visit?' and 'have you taken any medicines since your last visit, and if so, what for?'

The investigator will record the diagnosis or the symptoms if a diagnosis is not apparent, the date of onset and the date of resolution if appropriate. For events not specified in the DAIDS Toxicity Table (***Appendix 8***) the severity will be determined according to the CTCAE table version 4.02 http://www.acrin.org/Portals/0/Administration/Regulatory/CTCAE_4.02_2009-09-15_QuickReference_5x7.pdf

The relationship to vaccine will be determined by the investigator according to the definitions provided in **section 8**. All of this information will be recorded in the adverse event CRF.

7.5.2 General examination and vital signs

A general examination, and assessment of vital signs as described in **section 7.2.1**, will be performed at time points specified in **Appendix 2**, and at additional time points if indicated according to the answers given during the adverse event assessment. Physical examination will be directed during following visits based on signs and symptoms, vital signs and cervical/axilliary lymphadenopathy assessements will be performed at every visit.

7.5.3 ECG safety assessment

ECGs will be done for the safety assessment of MVA associated myo/pericarditis. ECG will be performed during screening/before enrolment to exclude volunteers with significant ECG findings. ECG will be performed during the 14 days that follow the MVA immunisation timepoint, at visits Visits 12 and 15 if there is clinical evidence for cardiologic disease or as indicated by the clinical investigator. Significant ECG findings leading to exclusion are defined as "Abnormality in ECG that could indicate risk or make interpretation of vaccine effects difficult according to the study operating procedures." According to this definition volunteers will be excluded who have:

1. Evidence in the ECG for cardiologic disease
2. Variations in the ECG which might not be clinically significant but will complicate later interpretation for post vaccination ECG results.

ECG with clinically significant findings, or features that would interfere with the assessment of myo/pericarditis, including any of the following:

- conduction disturbance (complete left or complete right bundle branch block or nonspecific intraventricular conduction disturbance with QRS >120 ms, an AV block grade II or III, or QTc prolongation [> 440ms])
- significant repolarization (ST segment or T wave) abnormality
- significant atrial or ventricular arrhythmia; frequent atrial or ventricular ectopy (e.g., frequent premature atrial contractions, 2 premature ventricular contractions in a row)
- ST elevation consistent with ischemia; or evidence of past or evolving myocardial infarction.

ECGs will be interpreted by competent study physicians and be graded as *normal*, *abnormal/clinically insignificant* and *abnormal/clinically significant.* Any abnormal finding will be commented in the case report form. All ECGs will be sent prior to enrolment to a cardiologist panel for review and approval. The final verdict on the ECG with regards to enrolling or not enrolling the volunteer into the study will rely on the cardiology panel decision. Members of the cardiologist panel will be named before study initiation.

An ECG will be repeated 14 days post MVA / saline vaccinations if there is clinical evidence for cardiologic disease or as indicated by the clinical investigator or at anytime whenever a volunteer comes with symptoms and signs suggestive of myo/pericarditis. Results will be interpreted and compared to baseline ECG results by competent study physicians and sent to the cardiologist panel for review and approval. Post vaccination ECGs will be graded as*normal*, *abnormal/clinically insignificant* and *abnormal/clinically significant*. Post vaccination ECG findings will be graded as *no changes, changes/not significant* and *changes/significant*as compared to baseline results. Any abnormal finding and changes will be commented in the case report form.

7.5.4 Routine laboratory parameters and urinalysis

A pregnancy test will be performed by analysis of a urine sample for Human Chorionic Gonadotrophin (HCG) collected from female participants at screening, the day of each immunisation and at the final visit. The analysis will be conducted by a member of the study team according to trial-specific standardised operating procedures.

Peripheral blood and urine will be collected and analysed, as described in **section 7.2.3**, for the following parameters at the time points specified in ***Appendix 2***, and at additional time points if indicated to further evaluate or follow up adverse events:

7.5.4.1 Blood

- Creatinine, ALT, total and direct bilirubin, glucose
- Hb, total WCC, neutrophils, lymphocytes, platelets
- Troponin I will be performed 14 days post MVA / saline vaccinations (Visits 12 and 15) if there is clinical evidence for cardiologic disease or as indicated by the clinical investigator.

7.5.4.2 Urine

- protein, blood

7.5.5 Solicited local and systemic clinical and laboratory adverse events

Various local and systemic adverse events are known to be associated with licensed vaccines, and are referred to as **'solicited adverse events'**. These include disturbances in routine laboratory parameters, and are described in **Table 2** below.

Information on solicited adverse events will be collected at the clinics before and 30 minutes after each immunisation through direct questions asked during a structured face to face interview and through examination. This information will be recorded directly into the CRF. Participants will also be asked to complete a diary card **(Appendix 6)** recording solicited adverse events that start within 7 days of each immunisation, regardless of the route of immunisation they receive. Diary cards will be reviewed and discussed later at the scheduled 2 weeks follow-up visit after each immunisation, when the clinician will also enter solicited adverse event data onto the CRF. Diary card entries (e.g. severity grade of symptoms) might

be revised after discussion between the participant and the clinician. Any alterations should be dated and initialled by the study clinician directly onto the diary card. Where there is a discrepancy for the grade assigned on the same day as the visit, it is assumed that the grade assigned by the clinician is more accurate. Any discrepant report between the diary card and the CRF entry will be foot noted in the study report and analysis tables

All participants will be asked to contact the study site in the case of adverse events of suspected grade 2 or more for evaluation within the 7 days post immunisation period. A decision to offer the volunteer a visit to the clinic should be made and documented in the source documents. All participants with suspected grade 3 or more events should be seen by the study physician for further evaluations and confirmation of the reported grade.

In addition, systemic laboratory adverse events will be collected through routine laboratory testing according to the schedule **(Appendix 2)**. These will be recorded on the standard laboratory reports and may be transcribed into the CRF or a copy of the laboratory report attached to the CRF.

Table 2 Solicited adverse events

Type	Event
Local AEs (immunisation site)	Local Pain<br>Local Itching<br>Warmth<br>Redness in cm<br>Swelling (soft) in cm<br>Induration (hard) in cm<br>Blisters (blood or clear) in cm<br>Papule in cm
Systemic Clinical AEs	Temperature<br>Chills<br>Myalgia/flu-like general muscle aches<br>Aching joints<br>Malaise (excess fatigue)<br>Headache<br>Nausea<br>Vomiting
Systemic Laboratory AEs	Creatinine, ALT, total and direct bilirubin, glucose<br>Hb, total WBC, neutrophils, lymphocytes, platelets

The events will be **graded** according to the toxicity table in **Appendix 8**.

Relationship will be recorded in the immunisation CRF but not on the diary card, on the assumption that any of these events starting within 7 days of an immunisation are at least possibly related. If the onset of the event is beyond 7 days, the event will be recorded on the adverse event CRF and a relationship determined by the investigator reviewing the event.

7.5.6 Procedure for follow-up of adverse events and pregnancy

The Investigator will make every effort to monitor all adverse events, regardless of severity, until resolution or stabilisation and to obtain documentary evidence of the outcome of pregnancy, in order to report this on the CRF during the trial. For volunteers who become pregnant before completing the vaccine series, no further immunisations will be given. A positive urinary pregnancy test result will be communicated to the participant as well as her ineligibility to continue with the immunisation schedule. Pregnant participants will be referred

to the Obstetrics and Gynaecology Department for the Mbeya Referral Hospital, MNH for further management and care. In Maputo, pregnancies occuring during the course of the study will be followed by obstetricians (study doctors) at the youth clinic at HCM, until delivery. The participant will be followed for all the remaining scheduled visits according to the schedule of procedures for safety evaluation. The maternal follow up during pregnancy, labour, delivery and post-delivery periods as well as the infant outcome will be documented in a Pregnancy Report CRF at various time points. Female participants will be required to use an effective contraceptive method until 3 months after the last vaccination, which is Visit 17. At this point the last pregnancy test will be performed according to the study flow schedule. After the database is locked and the trial is closed, any additional information about adverse events or pregnancy that comes to the attention of the investigator should be reported by email to the TMG and the Chief Investigator on behalf of the Sponsor. The study site will have available, free of charge, a variety of contraceptive methods (male and female condoms, oral and injectable contraceptives, etc). Counseling to prevent pregnancy and to use contraception will be performed on all study visits.

7.5.7 Serious adverse events

A serious adverse event is defined in **section 8**.

All serious adverse events should be reviewed by the Principal Investigator for the clinical centre, and discussed at the next Trial Management Group call.

A serious adverse event might cause a PI to consider discontinuing dosing or withdrawing a participant from the trial.

For all serious adverse events, the investigator will complete a serious adverse event CRF and report it to Monitors, TMG, Sponsor and DSMB, within 24 hours of learning of the event as described in **section 8.2**, and on to the ethical committees and regulatory authorities in the timelines required by each authority.

7.5.8 Other adverse events

Any other systemic or local events which are not listed as solicited within the 7 days post immunisation observation, or which are listed but occur after the 7 days post immunisation observation period will be regarded as an adverse event and need to be recorded in the Adverse Events CRF. These will be recorded as reported following an open question to volunteers, with the dates of commencement and resolution, the outcome at the end of the study, required treatment, relationship to the study agent and potential effects on the vaccination schedule. Where a diagnosis is possible, it is preferable to report this rather than a series of terms relating to the diagnosis. When reporting a syndrome, the associated signs and symptoms will be indicated in the comment section of the CRF following the syndrome rather than as separate events. All grade 3 or 4 adverse events or serious events and/or those that necessitate a physician's visit or are treated with a prescribed medication will be evaluated. The participants will be followed carefully until the condition is resolved and/or the cause is identified. Any medication or other therapeutic measure taken to relieve symptoms of the medical problem must be recorded on the appropriate case report form.

7.6 Criteria for stopping treatment

Volunteers/participants may discontinue further immunisations at any time if they wish to do so, for any reason. The date of discontinuation and reason for doing so will be recorded in a case record form.

The clinical investigator may decide that it is not in the best interests of the volunteer/participant to proceed to the next immunization if the investigator deems that

continuing might compromise participant wellbeing or interfere with the achievement of the trial's objectives. Participants who are discontinued due to a clinical or social adverse event (AE) will be followed up until the event has stabilised. The event should be reported as described in **sections 8.2.1 and 8.2.2.**

The following are examples of circumstances in which the clinical investigator would discontinue the injection schedule in a participant:

1. HIV infection
2. Pregnancy
3. Type 1-hypersensitivity associated with immunization.
4. Any medical condition that can recur/deteriorate with further immunizations.
5. Need for systemic glucocorticoids or immunomodulators other than NSAIDs for any reason.
6. The Sponsor, vaccine provider or principal investigator decides to stop or cancel the study.
7. The ethical or regulatory authorities decide that the study be stopped.

Trial volunteers who are discontinued from additional study injections will continue to be followed according to the schedule to further evaluate safety and monitor adverse events, unless they wish to withdraw from the study entirely, in which case they are free to do so. The frequency of visits and laboratory investigations may be reduced on consultation with the Principal Investigator and TMG. Participants who have received any immunisations should be asked to undergo the procedures scheduled for the primary endpoint visit as a minimum. They will also continue to be covered by the medical insurance until the end of the study period.

No serious adverse reactions are expected for these immunogens, and over 200 subjects have been exposed. However, this is the first time the DNA is being administered with electroporation, and the first time MVA-CMDR and CN54rgp140/GLA-AF will be administered at the same timepoint. In the event of a suspected serious adverse reaction this will be unexpected and will therefore be classified as a SUSAR (Suspected Unexpected Serious Adverse Reaction). Such events must be reported to the regulatory authoritiy within 24 hours if fatal or an immediate threat to life (see **section 8** for further details). The Chief Investigator will inform the Sponsor's Medical Expert and organise the expert multi-disciplinary review within 3 working days, and further immunisations in all participants will be suspended pending this review.

A component of the expert review will be to consider whether or not further immunisations for that group, or for all groups should be held whilst further investigation into the event is undertaken, and whether or not to call an unscheduled meeting of the Data Safety Monitoring Board (DSMB) to review the accumulated safety data for one or all treatment groups.

The DSMB will be asked to make a recommendation with respect to further immunisations in the trial. This recommendation will be shared with the local and national ethics committees and regulatory authorities.

The decision to stop further immunisations rests with the Sponsor, and the Sponsor reserves the right to stop the whole trial at any time, which could include following a recommendation from the DSMB, the local or national ethics committees, or the national regulatory authorities.

7.7 Procedures at the end of the trial

The trial will be closed when all participants have made their final follow-up visit (Visit 17 or optionally visit 18), the data entered into the database and the database locked. There will be a final monitoring/closeout visit to the clinical sites between the last visit and the database lock.

7.8 Sub study: particpants and community related perceptions

7.8.1 Rational and Backgroud of the sub study

Recruitment and acceptance for HIV vaccine trials are largely dependent on the knowledge, perceptions and atitude towards HIV vaccine development within the volunteers' communities. Following experiences of HIV Vaccine trials in Mbeya (RV 172 and TaMoVac I), Dar es Salaam (HIVIS-03 and TaMoVac I) and Maputo (TaMoVac I); members of the communities have misconceptions about HIV Vaccine trials thereby posing challenges towards recruitment and retention of trial participants. As these trials aim to eventually lead to efficacy studies being conducted within the communities, assessing community knowledge, perceptions and recommendations for strategies that will effectively engage volunteers in HIV Vaccine trials will be helpful in establishing trustful relations with trial researchers, minimizing misconceptions and consequently improving recruitment and retention of trial volunteers. The community awareness sub study will be the topic of the thesis of Mrs. Doreen Pamba from the NIMR-Mbeya Medical Research Center (MMRC) within her Master of International Public studies at the University of Liverpool, UK throughout TaMoVac II. The study will therefore mainly focus on Mbeya but the interpretation is expected to involve collaboration with the social science sections of the TaMoVac II Dar es Salaam and Maputo group. Any involvement of volunteers and researchers in Mozambique will be preceded by the respective ethics approval by the National Health Bioethics Committee of Mozambique.

7.8.2 Sub study objectives

1. To assess community awareness and perceptions regarding ongoing HIV Vaccine trials
2. To explore study participants' perceptions about HIV Vaccine trials
3. To inform researchers with community driven strategies that would effectively engage communities in HIV Vaccine trials and establish trust towards trial researchers.

7.8.3 Sub study population

Communities residing in areas where former HIV Vaccine trial recruitment has taken place as well as those who were not addressed by recruitment strategies but reside within the trial catchment area. In addition, potential study participants attending briefing sessions for TaMoVac II will also be included in the study sample.

7.8.4 Sub study methods

Data collection

Focus Group Discussions and questionnaires will be used to generate information regarding knowledge as well as perceptions of both communities and potential study participants towards HIV Vaccine trials. Such methods will also explore strategies recommended by communities and potential study participants to effectively engage them in HIV Vaccine trials as well as those that help to build up and maintain trust towards HIV Vaccine trial researchers. Data from focus group discussions will be recorded using tape recorders.

7.8.5 Ethical consideration and confidentiality

All interview and focus group discussion related data will be used coded to maintain confidentiality of interviewees. No personal identifiers will be used throughout data collection. Participants will be requested to withhold fellow participant responses, comments and identity within the group discussions.
Structured questionnaires and procedures for recruiting volunteers will be submitted to the local IRB at Mbeya, MUHAS and the National Health Bioethics Committee of Mozambique (as applicable) as ammendements for approval prior to sub study related activities.

8 SAFETY REPORTING

Tanzanian and Mozambican clinical trials regulations require that both investigators and sponsors follow specific procedures when notifying and reporting adverse events/reactions in clinical trials. These procedures are described in this section of the protocol. **Section 8.1** lists the definitions; **section 8.2** describes details of the responsibilities of the institutions/investigators.

8.1 Definitions and Classification

An **Adverse Event (AE)** is any untoward medical occurrence in a patient or clinical investigation subject administered a pharmaceutical product and which does not necessarily have a casual relationship with this treatment. An AE can therefore be any unfavourable and unintended sign (including abnormal laboratory finding), symptom or disease temporally associated with the use of a medicinal (investigational) product, whether or not related to the medicinal (investigational) product (ICH E6). An adverse event in this study is any adverse experience occurring during the course of the study starting after enrolment.

An **Adverse Reaction** is any untoward and unintended response in a subject to an investigational medicinal product which is related to any dose administered to that subject.'Related' means possibly, probably or definitely as defined below.

An **Unexpected Adverse Reaction** is defined as an adverse reaction of which the nature or severity is not consistent with the information about the medicinal product in question set out in the Investigator's Brochure. Any AR that is considered possibly, probably or definitely related to study product according to the classification below meets the criteria for "unexpected".

A **Serious Adverse Event (SAE)** or Serious Adverse Reaction (SAR) or Suspected Unexpected Serious Adverse Reaction (SUSAR) is any adverse event or adverse reaction if it results in the following (ICH E6 and E2A):

1. Death,
2. A threat to life at the time of the event
3. Requires in-patient hospitalization or prolongs existing hospitalization (hospitalization for an elective treatment is not considered a Serious Adverse Event)
4. Results in persistent or significant disability or incapacity
5. Is a congenital anomaly
6. Is any other important medical condition*

*Examples of conditions regarded as "any other important medical condition" include allergic bronchospasm requiring intensive emergency treatment, seizures or blood dyscrasias, which did not result in hospitalization or development of drug dependency.

Note: Grade 4 lab abnormalities are not serious adverse events, unless they are related to a clinical event matching the SAE definition. Any grade 3 and 4 lab event needs to be confirmed within 48 hours.

Note: Interventions or hospitalization for elective treatment, e.g. cosmetic surgery, tubal ligation or sterilization, hospitalization due to labour are not considered as adverse events unless complications related to the elective treatment occurs.

Any HIV infection occurring throughout the study period will not be defined as a Serious Adverse Event but needs to be reported within 2 working days of awareness (see section 8.2)

Any Social Harm related to study participation or vaccination will be defined and graded as an Adverse Event *(e.g. denial of health and life insurance, employment, immigration and marriage relationships).*

8.1.1 Adverse Event Grading and Relationship

The severity of adverse events will be determined by the investigator, based on the DAIDS Table for Clinical and Lab Criteria for Definitions **(*see Appendix 8*)** or if the event is not listed the event will be graded following these guidelines:

- Mild (Grade 1): Transient or mild symptoms; no limitation in activity; no intervention required. Symptoms causing no or minimal interference with usual social and functional activities
- Moderate (Grade 2): Symptoms causing greater than minimal interference with usual social and functional activities. No or minimal intervention required. It is uncomfortable or an embarrassment.
- Severe (Grade 3): Symptoms causing inability to perform usual social and functional activities. Medical intervention often required.
- Potentially life-threatening (Grade 4): Symptoms causing inability to perform basic self-care functions OR Medical or operative intervention indicated to prevent permanent impairment, persistent disability, or death.

The relationship of adverse events to study product will be classified as:

**Unrelated**	adverse events that can be clearly explained by extraneous causes and for which there is no plausible association with study product, or adverse events for which there is no temporal relationship
**Unlikely to be**	adverse events that may be temporally linked, but which are much more likely to be due to other causes than study product and which do not get worse with continuing use of product
**Possibly**	adverse events that could equally well be explained by study product or other causes, which are usually temporally linked and may improve when not using study product but do not reappear when using study product
**Probably**	adverse events that are temporally linked and for which the study product is more likely to be the explanation than other causes, which may improve when not using study product
**Definitely**	adverse events that are temporally linked and for which the study product is the most likely explanation, which disappear or decrease when not using study product and reappear when using study product

A severe adverse event is any **graded 3 or 4** by the criteria in the DAIDS Toxicity Table (Appendix 8). Some, but not all grade 3 and 4 adverse events will be "serious" by ICH GCP criteria above (section 8.1).

8.2 Adverse events that require expedited reporting by the centres

Adverse and serious adverse events will be recorded on the Adverse Events case record form. Any severe clinical adverse event (grade 3 or 4), any serious adverse reaction or unexpected adverse reaction should be reported within 24 hours after site awareness to the Study Monitors, Sponsor, TMG, WRAIR and DSMB, and on to the Tanzanian and Mozambican ethics committees and regulatory authories according to each authority's requirements. Any grade 3 or 4 fever must persist for 48 hours to meet the definition of severe fever..

Although post immunisation solicited local and systemic adverse events are documented separately from AEs, they are reported as above if they meet SAE or study pause rule definitions. Therefore the PI or designee must review both Vaccine/Reaction and Adverse Event CRFs to ensure prompt and complete identification of all the events described above that require expedited reporting.

Expedited reporting within 24 hours of the site awareness of the event is warranted for:

1. A clinical or social harm event resulting in a decision to discontinue the vaccination schedule
2. Administration of the incorrect vaccine or placebo
3. Any serious adverse events
4. Pregnancy
5. HIV infection

8.2.1 Procedures at the centres for reporting expedited events

Reporting of events that require expedited reporting by the centres, including serious adverse events, will mostly be done by email to the group listed below, but can be done by telephone or fax to the Chief Investigator, Prof. Lyamuya and the Sponsor's Medical Expert, Prof. Eric Sandstrom. . The minimum criteria required in reporting an event are the volunteer identifiers (trial number, site and participant code, date of birth), reporting source (name of the site, the reporting site investigator and the site PI), a narrative description of the event, grading of the adverse event, the onset of the adverse event and current status, the relationship to the study agent and why the adverse event merits expedited reporting. . Associated medication, lab information and other relevant clinical information shall be provided as soon as possible.

Adverse Event Reporting contacts are:

Name	***Position***	***email***
For the TMG		
Eligius Lyamuya	Chief Investiagator, MUHAS	eligius_lyamuya@yahoo.com
Eric Sandström	Protocol chair and SMI's Medical Expert, KI	eric.sandstrom@sodersjukhuset.se
Kisali Pallangyo	MUHAS's Medical Expert	kpallangyo@gmail.com
Arne Kroidl	Protocol co-chair, LMU	akroidl@mmrp.org
Sheena McCormack	Protocol co-chair, MRC CTU	TaMoVac@ctu.mrc.ac.uk
Muhammad Bakari	Site PI, MUHAS	drbakari@yahoo.com
Leonard Maboko	Site PI, MMRC	lmaboko@mmrp.org
Ilesh Jani	Site PI, INS/CISPOC	Ilesh.jani@gmail.com
Bahati Kaluwa	Co-PI, MMRC	bkaluwa@mmrp.org

Merlin Robb	Consultant for MVA, WRAIR	mrobb@hivresearch.org
Britta Wahren	Consultant for DNA, SMI	britta.wahren@smi.se
Patricia Munseri	Site CRC, MUHAS	pmunseri@yahoo.com
Marco Missanga	Site co-CRC, MMRC	mmissanga@mmrp.org
Philipp Mann	Site co-CRC, MMRC	pmann@mmrp.org
Edna Viegas	Site CRC, CISPOC	edna_viegas@hotmail.com
Gunnel Biberfeld	Consultant for immunology, SMI	gunnel.biberfeld@smi.se
Sayoki Mfinanga	Consultant for database,NIMR	gsmfinanga@yahoo.com
For the Sponsor		
Eva Netterlid	SMI, Sponsor	eva.netterlid@smi.se
Muhsin Aboud	MUHAS Sponsor	maboud@muhas.ac.tz, abouduhsin@gmail.com
For the External Monitor		
Gail Smith	External monitor, WRAIR	gsmith@hivresearch.org
For the DSMB		
Innocent Semali	Co-Chair of DSMB; Tanzania	isemali@muhas.ac.tz
Esperança Sevene	Co-Chair of DSMB; Mozambique	esevene68@gmail.com

The reporting clinicians cannot assume that the report has been received without at least one of the Chief Investigator, Medical Experts or Protocol co-Chairs having acknowledged receipt of the report, and endorsement of onward reporting to the authorities.

8.2.2 Reporting Requirements to the Tanzanian and Mozambican IRB

Each site PI will be responsible for providing all Safety Reports, Continuous Progress Reports and reporting all SAEs, unexpected AEs, social harms, and major deviations or consent withdrawal/dropouts to their local and national authorities in a timely manner. These include MUHAS and MMRC local IRBs, NIMR and TFDA in Tanzania, and the National Bioethics Committee of Mozambique and the Mozambican Regulatory Authority (Pharmaceutical Department).

Chairman, Medical Research Coordinating Committee
National Institute for Medical Research (NIMR)
P.O. Box 9653, Dar es Salaam, TANZANIA
E-Mail: headquarters@nimr.or.tz

Chairman, Mbeya Medical Research and Ethics Committee
Mbeya Referral Hospital
P.O. Box 419, Mbeya Tanzania
E-Mail: esamky@yahoo.com
Tel: +255 25 2503577

Chairman, Senate Research and Publication Committee
Muhimbili University of Health and Allied Sciences (MUHAS)
P.O. Box 65001, Dar es Salaam, Tanzania
E-Mail: drp@muhas.ac.tz
Tel: +255 22 2151467

Director General, Tanzania Food and Drugs Authority (TFDA)
P.O. Box 77150, Dar es Salaam
Tel: +255 22 2450512 or 2450751
Fax +255 22 2450793
E-Mail: info@tfda.or.tz

Chairman, National Health Bioethics Committee of Mozambique
Ministry of Health P.O. Box 264, Maputo, Mozambique
Tel: +258 21 306620, Ext: 382
E-Mail: jo.ter@tvcabo.co.mz

Pharmaceutical Department of Mozambique
AV. Salvador Allende/Av. Agostinho Neto, Maputo, Mozambique
Tel: +258 21 303473
Fax: +258 21 326547
E-Mail: farmacovigilanciacimmocambique@gmail.com

9 STATISTICAL CONSIDERATIONS

By the end of this study up to 198 study participants will have been exposed to DNA, MVA with and without CN54rgp140 adjuvanted with GLA-AF or saline. Participants will be randomized to vaccine or placebo in a 10:1 ratio.

9.1 Method of randomisation

Participants will be block-randomised centrally using a computer-generated algorithm.

9.2 Outcome measures

9.2.1 Primary outcomes

9.2.1.1 Immunogenicity

- The primary **immunogenicity** endpoint will be determined from the following results:
    - a positive response in the HIV peptide-specific γ-IFN Elispot assay using freshly isolated PBMCs collected 2 weeks after the final vaccination. A positive result will be defined as the numbers of spot-forming cells (SFC) more than four times the background (RPMI medium only) and > 55 SFC per million PBMC (Merck definition)
    - the magnitude of the γ-IFN Elispot assay response which will be restricted to those samples which have been defined as positive, as measured by the SFC 2 weeks after the final vaccination
    - the endpoint titre or concentration of systemic IgG CN54rgp140 specific antibodies measured 2 and/or 4 weeks after the final vaccination
    - the presence of neutralising antibodies to tier 1 and tier 2 viruses and the titre when these are present

9.2.1.2 Safety

The primary **safety** endpoint is a grade 3 or above local or systemic clinical and laboratory solicited adverse event (see **section 7, Table 2**) or any grade of adverse event that results in a clinical decision to discontinue further immunisations.

9.2.2 Secondary outcomes

9.2.2.1 Immunogenicity

- Breadth of γ-IFN Elispot cellular immune responses to gag at 2 weeks after the final immunisation
- Frequency, breadth and magnitude of cellular immune responses at other time points (2 weeks after last DNA, 2, 12 and optionally 24 weeks after the first MVA +/- CN54rgp140/GLA-AF timepoint.
- CD4+ and CD8+ T-cell responses to HIV peptides as measured by ICS. The frequency and the functional quality of vaccine induced T cell responses will be studied at different time points
- The magnitude of T cell proliferative responses to HIV peptides at different time points.
- The frequency and magnitude of systemic IgG and IgA binding antibody responses to HIV antigens of other clades at other time points.

Other assays of antibody phenotype/function may include, but will not be limited to: the duration and avidity of envelope specific binding antibody responses, the frequency and titre of ADCC responses, the frequency and titre of ADCVI responses, the breadth of Envelope specific antibody responses, the frequency and titre of binding V1V2 specific IgG antibodies (linear and/or scaffolded protein) and the extent to which systemic antibodies bind to CD4.

9.2.2.2 Safety

- Any grade of adverse event that occurs in a participant that has received at least one immunisation.

9.3 Sample size

One hundred and eighty (180) individuals will be randomised to an active product group for the purpose of the analysis, with a further 18 individulas randomly allocated to receive saline for the purpose of blinding the laboratory and clinical staff and to reinforce the message that participants should not considered themselves protected against HIV. There will be two randomisations, the first to compare the three DNA treatment groups, each of which will have approximately 60 participants, and the second to compare two groups, namely MVA-CMDR given with CN54rgp140/GLA-AF and MVA-CMDR alone, each of which will have approximately 90 participants.

Endpoint considerations for the purpose of calculating sample size
The primary endpoints for the first and second randomisations can be expressed as a proportional outcome, such as the proportion of responders, or as a summary characteristic of a continuous outcome, such as the mean or median magnitude of the SFC response to any CMDR pool, or the mean or median titre of binding or neutralising antibodies. A further example of a proportional outcome would be the proportion of subjects that had a clinically relevant cellular response AND a clinically relevant antibody response. For the purpose of calculating sample size we considered two possibilities:

Continuous variable (Magnitude of cellular response)
An important difference in the magnitude of cellular responses will be defined as a doubling of mean SFC/10^6response in any CMDR pool, excluding the negative responders.

Proportion (Frequency of responders)
An important difference in proportions will be defined as a 30% absolute difference in the frequency of responders by group.

Statistical assumptions
Alpha will be reduced to 2.5% (p=0.025) to adjust for multiple comparisons of the first randomisation, and will be 5% for the second randomised comparison. The following tables illustrate the power to detect a difference in a continuous variable or proportion with 60 or 90 subjects per group, using magnitude of response, and frequency of responders respectively.

Power to detect differences in a <u>continuous variable</u>:

Increase in SFC	Power when n=60 per group	Power when n=90 per group
100% increase (doubling)	85	98
75% increase	66	90
50% increase	38	66

Note: Based on $\log_{10}$ transformed SFC, assuming a standard deviation of 0.5.

Power to detect a range of differences in a proportion:

Increase in absolute difference (control to group of interest)	Power when n=60 per group	Power when n=90 per group
40% to 70%	86	98
40% to 60%	48	77
50% to 80%	89	99
50% to 70%	50	78

60 participants per group provides adequate power (≥85%) to detect a doubling in mean magnitude or to detect a 30% absolute difference in the proportion of responders however the latter is defined. However, there is insufficient power to detect smaller differences, which could still be clinically meaningful. Ninety participants per group also provides adequate power for detecting a 75% increase in the magnitude of immune responses.

9.4 Interim monitoring and analyses

Analyses will be performed at MUHAS by the Trial Statistician, who will be supported bythe Statistical Mentor from the MRC CTU in London.

The accumulating safety data will be reviewed once by the DSMB (see also **section 15.5**) after half of the participants have completed the immunisation schedule. The DSMB will make a recommendation to the statistical team should any modifications to the safety analysis plan or statistical tables be required.

An unscheduled meeting of the DSMB may be required **(see section 7.6)**, in which case the DSMB will make a recommendation to the Sponsor about whether or not to continue further immunisations in the trial.

9.5 Data analyses and presentations

A full statistical analysis plan will be developed before the main analysis of the trial is performed. It will be based on the following populations and variables.

9.5.1 Participant populations

- Intention-to-treat (ITT) population: all participants randomised and given at least one immunisation in the trial.
- Per-protocol (PP) population: all participants randomised and immunised with all scheduled immunisations, and who complete the trial with no major protocol deviations.

9.5.2 Immunogenicity variables

IFN-γ Elispot responses will be measured using freshly isolated PBMCs which will be purified from whole blood. Cells will be stimulated using pools of peptides derived from the vaccine and also negative and positive control stimuli. The number of spot-forming cells (SFC), indicative of IFN-γcytokine release will be enumerated using an automated Elispot reader. A positive result will be defined as numbers of spot-forming cells (SFC) more than four times the background (RPMI medium only) and > 55 SFC per million PBMC (Merck definition).

The proportion of CD4+ and CD8+ T-cells producing particular cytokines/chemokines and expressing cytolytic markers in response to HIV-1 peptides (as used in the Elispot assay) will be assessed. Frozen PBMCs will be used and thawed prior to analysis according to established methods. PBMCs will be stimulated *in vitro* and T-cell subsets and cytokine responses described using multi-parameter flow cytometry. Subsets of T-cells will be identified on the basis of the expression of cell surface markers and the percentages of CD4+ and CD8+ T-cells producing cytokines/chemokines will be determined with reference to the responses to control stimuli.

Binding antibodies with specificity for HIV antigens (Clade A, C and E) including those specific for the immunogen CN54rgp140 will be measured using standard ELISAs. ELISA absorbance readings for binding antibodieswill be classified as 'response' or 'no response' using pre-defined cut-offs. Only samples with a detectable antibody response will be subjected to endpoint titration. In cases when a sample is positive for the presence of binding antibodies to CN54rgp140, the absolute concentration of antibody will also be determined. The concentration will be calculated relative to a commercially available standard purified antibody which will be titrated on the same plate as sample sera. Assays for the detection of neutralizing antibodies to HIV subtypes A, C and E will be performed using standardised assays including those using the TZMBL cell line and PBMCs.

All the immunogenicity endpoints will be signed off by the Laboratory Investigators. In the unlikely event that the Laboratory Investigators cannot reach consensus on a response, an expert Endpoint Panel will be assembled to make a final decision.

Prior to the main analysis, the Statistical Analysis Plan will be finalised describing the methods for comparing the groups in detail. The decision to compare the proportion of responders, or the magnitude of response, and the definition for these parameters will be informed by the full analysis of TaMoVac 01 and the results of other relevant clinical trials, with the aim of defining a 'clinically meaningful result'. Proportions will be presented with a confidence interval and groups compared using Fisher's exact test. Response magnitude will be compared between groups using rank tests.

9.5.3 Safety variables

The original verbatim terms used by the investigator to identify AEs in the CRFs will be coded using an appropriate medical coding scheme (MedDRA). In all summaries, if a participant reports the same system organ class or preferred term more than once then the worst severity and worst relationship to trial vaccine will be taken. Discrepancies between diary card and CRF reports will be queried and resolved. It is assumed that the grade assigned by the clinician is more accurate, and this will be the grade reported in the tables. If the diary card grade is worse, this will be foot-noted.

All safety end-points will be graded by the Clinical Investigators and reviewed by the Trial Management Group. In the unlikely event that the TMG cannot reach consensus about grade and relationship to study product, an expert Endpoint Panel will be assembled.

Safety outcomes will be reported overall with proportion and 95% confidence interval, and by group and time-point, and by relationship to study product.

For the primary analysis of safety endpoints (as defined in **section 9.2.1**), results will be expressed as a proportion with confidence interval, and groups compared using Fisher's exact test.

10 DATA MANAGEMENT

Data management, analysis and reporting of all trial data will be prepared according to detailed Data Management and Statistical Analysis Plans. NIMR will be the Data Coordinating Centre (DCC) with primary responsibility for data coordination, supported by the MRC CTU in London.

Any data analyses carried out independently by investigators should be submitted toNIMR before publication or presentation and be reviewed by the TaMoVac Steering Committee (see **sections 15.3 and 16**).

10.1 Data management at the Clinical Centre

Staff at the clinical centre will be responsible for:

- Entering relevant information in the clinical notes and holding a record for each participant which includes the CRFs with any changes made signed and dated
- The accurate completion of the CRFs
- Collection of the diary cards from participants
- Notification of SAEs within 2 working days of becoming aware of the event as indicated in Section 8.2
- Notification of pregnancy within 2 working days of becoming aware of the pregnancy as indicated in Section 8.2

Data will be recorded directly onto the clinical notes and the CRFs in the most logical order, which may not necessarily always be the clinical notes followed by the CRF. For example the recording of the solicited adverse events will be done directly onto the CRF with additional details provided in the clinical notes if required. Similarly if the screening CRF does not reveal any medical history of relevance then this negative finding will not be duplicated in the clinical notes. Additional detail will be expected should there be a clinical abnormality relevant to eligibility. The dates of visits including immunisation, dates and results of pregnancy tests, and details of clinical management (description of adverse events and concomitant medication) will be detailed in the clinical notes.Other source data are described in **Table 5**. Data entry from CRFs will be performed at the sites by data entry clerks who are under supervision of a site data manager. All data will be double entered into a computerized database. An internal data monitoring with inconsistency and logic checks will run after double data entry. The resolution of queries and clearance of inconsistent data will be performed by the site data manager, and may require communication with the clinical team. Consistency checks and range checks will be performed at the data entry level on a daily basis.

Table 5: The following table identifies the source documents:

Type of data	Source document
Informed consent	Signed informed consent form
Medical history	Clinical notes, screening CRF and Previous Condition CRF Forms
Physical examination, including height and weight	Clinical notes and Physcial Examination CRF Form, visit checklist
Demographics	Registration sheet and Screening CRF Forms
Laboratory data	Laboratory reports
Date/time of routine bloods; urinalysis dipstick and urine pregnancy test sampling and results	Clinical notes or visit checklists and Laboratory CRF Forms
Date/time/result of solicited AEs including vital signs measurements post immunisation	Clinical notes or visit checklists and Vaccine/Reation CRF Form
Date/time of immunisations	Clinical notes or visit checklists and Vaccine/Reation CRF Form
Date/time of immunogenicity sampling	Clinical notes or visit checklists
Immunogenicity results	Core Immunogenicity Laboratory File
IMP accountability	Pharmacy File
Adverse events	Clinical notes and Adverse Events CRF Form
Concomitant medication	Clinical notes and Concomittant Medication CRF Form
Protocol deviations	Clinical notesand Protocol Devistion CRF Form
Participant reported solicited AEs and concomitant medication	Diary cards

CRFs will be designed by Data Coordinating Center and provided to the site.

All data will be entered legibly in blue or black ink with a ball-point pen. If an error is made, the error will be crossed through with a single line in such a way that the original entry can still be read. The correct entry will then be clearly inserted and the alterations will be initialed and dated by the person making the alteration. Overwriting or use of correction fluid will not be permitted.

To preserve confidentiality, the CRFs will not bear the participant's name. The participant's date of birth and trial number (which will have been given at screening **section 4.4**) will be used for identification.

Laboratory reports must be signed by a member of the clinical trial team. In the event of an abnormality, an indication should be given whether or not action was taken, the date of review and the signature of the clinician reviewing the result.

CRFs and clinical notes should be kept in a secure location for 2 years after the last approval of a marketing application or until 2 years have elapsed since formal discontinuation of product development, and at least 15 years after the clinical trial has ended.

10.2 Data management in the immunology laboratories

Standardized operating procedures will be followed in all laboratories to ensure the quality of data. Data will be stored in both hard copies and electronically in an agreed format. Standard operating procedures will be followed in all laboratories to ensure the quality of the data. Data will be stored electronically in an agreed format and datafiles transferred to the Trial Statistician for the main analysis.

10.3 Data management at the Central Data Management Centre

The Data Coordinating Center (DCC) is located at NIMR, Dar es Salaam under the supervision of Dr Mfinanga. Mentorship will be provided by the MRC CTU under the supervision of Dr McCormack. NIMR will be responsible for the development and support of the trial database at each centre, and for the combined database held centrally at NIMR on behalf of the investigators. Guidance will be provided from the MRC CTU.

Central checks of the combined database will focus on the parameters required by the Trial Statistician for the analyses. Queries will be raised with the site senior data managers.Copies including documentation detailing all the queries and changes will be stored in a study specific binder at the DCC.

11 TRIAL MONITORING

11.1 Continuous and Internal Study Monitoring

Continuous study monitoring will be performed by the site teams, the Medical Experts and protocol co-Chairs and the Trial Management Group (TMG) who will closely monitor and review the study progress, adverse events reports, reactogenicity reports, protocol deviations, lab data and any other data available from the database. Internal study monitoring will be performed continuously at the study site by assigned internal monitors and the CRC. In addition regular internal monitoring visits will be performed by the Sponsor's Medical Expert. . This will include review of the study binders, source documents and case report forms, the study clinics and pharmacies, trial procedures and the need for training. Internal laboratory monitoring at the study laboratories will be done by Charlotta Nilsson from the SMI.

11.2 TMG and safety calls during the study

Telephone conference calls will be held every 2-4 weeks and upon request/need. Calls will be organized by the UK MRC Clinical Trials Unit.TMG members will meet through these calls to up-date on the study progress, discuss adverse events, reactogenicity and protocol deviations and assist the site TMT in case management and resolutions. Prepared data management reports will be heard and reviewed. Laboratory and organisational issues will be discussed. Conference call agendas, minutes and follow-ups will be generated and distributed.

11.3 Clinical site monitoring

External trial monitoring will be conducted by WRAIR. External monitoring will be performed at the study sites through regular monitoring visits as agreed with the Sponsor. The external study monitor will be responsible to ensure that the trial is conducted according to ICH GCP guidelines including monitoring vaccine data consistency, vaccine accountability, dispatch and arrival of immunological specimens. A monitoring plan will be established prior to study initiation.

WRAIR will produce a detailed Monitoring Plan. The trial site will be monitored to ensure:

- the completeness and accuracy of the data entered on the CRFs
- compliance with the protocol and principles of GCP
- proper maintenance of all trial documentation
- complete IMP accountability
- smooth day-to-day running of the trial

All documents generated by the trial site which form part of this trial, and the ensuing data, must be made directly available in order that the monitor can verify, using source documents, the data in the CRFs. This procedure is termed Source Document Verification (SDV).

The PI agrees to allow GCP audits of the trial site and all trial documentation by the Sponsor or its representatives.

11.3.1 Direct Access to Data

Participating investigators should agree to allow trial-related monitoring, including audits, ethics committee review and regulatory inspections by providing direct access to source data/documents as required. Participants' consent for this is obtained as part of the consent process.

11.3.2 Archiving and Data ownership

Study related documents including clinical notes, completed CRFs and original laboratory results, hard copies and electronic copies will be kept in a secure location at MUHAS, MMRC and INS. The data generated in this study will be the property of the TaMoVac Investigators and will be held on their behalf by MUHAS, NIMR, MMRC and INS until completion of the study or until it is stopped. Thereafter the data will be passed over to MUHAS, NIMR, MMRC and INS for archival purposes according to accepted regulation for a minimum of 15 years. Data for e.g. continuous progress reporting or presentations during the internal TaMoVac investigator meetings will be available during the study. In general the use of study data for any purpose will need to be approved by the TaMoVac Steering Committee.
WRAIR will require access to all data in order to meet obligations with full reporting of data to the FDA regarding any use of the MVA product. KI/SMI will maintain data in accordance with US FDA guidelines until 2 years after licensure or retiring the IND.

11.4 Monitoring by the Trial Management Group

The Trial Management Group (described in section 15.1) will monitor the following using a reporting template:

- Screening, enrolment and screen:enrolment ratio for each clinical centre
- Immunisations completed (first, second, third, fourth, fifth) and any missed or outside the window
- Adverse events of note
- Missed visits and loss to follow-up
- Logistical difficulties at the clinical centres
- Data management issues (timeliness of CRFs completeness)
- Immunology core lab issues (completeness of specimens, next batch transfer or analysis)
- GCP issues (minor or other breaches)

11.5 Confidentiality

All personal details of the participants and the results of the trial will be kept strictly confidential. The Sponsor, as represented by Eric Sandstroem, will not keep any material on file containing the volunteers' full names; this information will be kept by PIs in the clinical trial facilities in a secure location. The confidentiality of volunteers will be respected and maintained at all times.

Each participant's physician will be informed of the nature and timing of the trial and asked to complete a brief questionnaire.

11.6 Quality Assurance and Quality Control of Data

Sites must not screen any volunteers until all approvals appropriate to the site are in place, and the sponsor or representative has conducted a site initiation visit. The Sponsor approval is dependent on receipt of an approval from the Tanzanian FDA, the Mozambican Regulatory Authority (Pharmaceutical Department) and approval from the Research Ethics Committee (REC) in MUHAS, MMRC, NIMR and INS.

It will be the responsibility of the PIs to ensure the accuracy of all data entered in the CRFs at their respective sites. They must conduct the trial personally, or delegate to members of

their research team specific tasks using a delegation log. They must ensure that each member of their research team is suitably qualified to perform delegated tasks by education, training and experience, and must ensure that written procedures are followed to enable collection high quality data.

12 ETHICAL CONSIDERATIONS AND APPROVAL

12.1 Ethical issues

There are two aspects of this trial that raise ethical issues

Firstly, because the product under investigation is a **candidate HIV vaccine**, and HIV is transmitted sexually, it is necessary to collect sensitive personal information and the volunteers might need to undergo a genital examination. The nature of the product may lead volunteers to erroneously conclude they are protected against HIV and to engage in riskier behaviour as a consequence. It is possible that following immunisations, participants may have equivocal results in the standard laboratory tests for HIV. However, it will be possible for any accredited laboratory to distinguish between a post-vaccination response and natural infection using routine assays.

Secondly **the reimbursement** to compensate for the intense follow-up schedule, which is a feature of healthy volunteer trials, could be sufficient incentive for individuals to take part against their better judgement.

12.2 Ethical Considerations

This study will be conducted according to ICH GCP guidelines and the Declaration of Helsinki (Version 2008), and it is the responsibility of the Clinical Investigators to ensure adherence. Before the study starts all personnel involved in the clinical and laboratory trial units will be trained for Good Clinical Practice (GCP) and/or Good Laboratory Practice (GLP) respectively. Study personnel will also be trained on standard operating procedures (SOPs) that are related to the protocol and will follow the GCP and GLP guidelines.

This protocol has been submitted to the National Ethics Committee at the Tanzanian National Institute of Medical Research (NIMR) for ethical approval. NIMR is registered with the Office for Human Research Protections (OHRP), US (FWA00002632). In parallel the proposals will be submitted to the institutional ethical committees. These are the Research and Publications Committee at the Muhimbili University of Health and Allied Sciences (MUHAS) having the FWA 00004301, and the Mbeya Municipal Medical Authorities in Mbeya (FWA00002469), and subsequently to the appropriate ethics committee in Sweden and Germany. In Mozambique the protocol has been submitted to the National Health Bioethics Committee (FWA00003139). The DNA and MVA vaccines, which will be donated by the KI/SMI in Sweden, have been approved by the Swedish Medical Products Agency and are currently used in the ongoing clinical trials in Sweden. The US Food and Drug Authority has previously approved the MVA which is in ongoing clinical trials in the USA. Approval of these immunogens will also be undertaken independently by the Tanzania Food and Drug Authority (TFDA) and the Mozambican Regulatory Authority (Pharmaceutical Department). The KI/SMI in Sweden will be responsible for providing the Investigator's Brochure (IB) for DNA as well as the Investigator's brochure for MVA as well as generating the randomization list.

Any changes or amendments in the protocol will be reported and approved as Memo to the IRBs or re-submission to the ethical and regulatory authorities. Safety assessment, monitoring and reporting will be assured as lined out in this protocol and responsibilities are defined. Continuous progress reporting will be provided as requested.

No study materials or data will be obtained from study volunteers before approval from the relevant bodies. Volunteer/participant information and data will be protected and coded and

only de-identified data will be provided for data entry. Access to volunteer/participant information for study schedule administration and tracing will be restricted to selected individuals only at the study sites. Access to study binders will be restricted to the clinical study site members and the internal and external monitors during monitoring visits. Representatives from the sponsor or ethical and regulatory authorities will be allowed to access study binders during inspections or audits and will be bound to confidentiality rules. Access to de-identified volunteer/participant data from the database outside the study sites will be restricted to identified study monitors and members of the Data Management Centres.

13 INDEMNITY

13.1 Insurance for investigators

The Principal Investigators will ensure that relevant clinical and laboratory staff engaged in the study are covered for possible legal action against the investigators for harm through indemnity insurance schemes, which will be paid for by the project during the study period.

13.2 Health care Insurance for trial volunteers

In Tanzania, all trial volunteers will receive health insurance provided by the National Insurance Corporation of Tanzania Limited which will cover for injury or death related to the trial through insurance schemes. These will be confined to the period of the study. For Mozambique, all events, whether immediate or late events (up to 2 years after the end of the study), which are proven to be associated to the study product, including death, will be covered by Trial Insurance.

Vecura Company in Sweden will have an insurance that covers any defect in the production of the DNA vaccine only.

SMI will in the role of sponsor assume the liability for the DNA vaccine and through separate collaborative agreements for the MVA-CMDR, the CN54rgp140 and GLA-AF.

14 FINANCE

14.1 Compensation for study participation

Regular compensations will be made to all volunteers/participants to cover their travel expenses as well as time and inconvenience compensation. For scheduled visits this will amount to 20,000 TShs (~ 12 USD) for Dar es Salaam and 15,000 TShs (~ 10 USD) for Mbeya where transport and living costs are lower compared to Dar es Salaam. In Maputo compenstation for scheduled visits will be 400,00 Mts (~ 14 USD) . Unscheduled visits will be compensated with 10,000 TShs in Dar es Salaam and 8,000 TShs in Mbeya and 200,00 Mts in Mozambique. Compensation for unscheduled visits might be rejected in the case of assumed abuse of compensation, which will be up to the study team's decision. During the study volunteers/participants will receive hypoallergenic latex condoms free of charge. Treatment and care for minor diseases can be provided by the study provided free of charge and might include diagnostic procedures as well as medications such as painkillers, antipyretics, antibiotics or anti malaria treatment, iron substitution etc. In case of hospitalizationor if further care will be required, these will be covered under the medical insurance provided by the National Insurance Corporation of Tanzania Limited. In Mozambique ageements with the HCM and the Polana Caniço Health Center will assure that the volunteers who need hospitalization and/or further medical requirements are assisted; these will be confined to the period of the study.

15 TRIAL COMMITTEES

15.1 Trial Management Team (TMT)

This group will oversee the day-to-day site procedures of the trial and the members will be primarily the clinical, laboratory, pharmacy and data management teams from each study site. Each TMT will be chaired by the site PI and/or his designee supported by the clinical coordinator, study secretary, community outreach officer, laboratory coordinator, study pharmacist, study nurses and data manager. Regular TMT meetings will be conducted. Notes will be taken and will form the basis of the progress report to the Trial Management Group. It will be the responsibility of the PI and/or his designee to assure on timely adverse events deviations or other unexpected events reporting, on the study progress as well as on regular continuous progress reporting to the ethical committees and regulatory authorities as requested also communicated to the TMG and TaMoVac Steering Committee. The TMT will assist and present during the Community Advisory Board (CAB) meetings.

15.2 Trial Management Group (TMG)

The supervision of the trial will be a responsibility of the Trial Management Group (TMG). The members will include the Chief Investigator, the Study Chairs, the site Principal Investigators, clinical coordinators, representative from WRAIR and IC, laboratory coordinators and data managers. The TMG will meet through research safety calls as indicated as well as during annual investigator meetings. The TMG will oversee and recommend on the study progress, clinical, data management, laboratory and pharmacy issues as well as study coordination procedures. This committee will be the central body to oversee adverse events and protocol deviations, to generate decisions about grade of adverse events and relationship to study vaccine as well as support for the TMT in case management. The TMG will also be the central body to collect and discuss progress reports on triggering adverse events providing information and recommendations to resume or terminate enrolment and vaccination after study pauses. Notes of TMG meetings will be kept and reported to the TaMoVac Steering Committee and Study Sponsor. Routine reports such as 6-monthly progress reports will be generated by the site PI's or his designee and sent to the the ethical committees and regulatory authorities in Tanzania and Mozambique including notification for the Study Sponsor and the TaMoVac Steering Committee. Expedited reports will be generated by the site PI or his designee and reviewed by the TMG before sending these to the Study Sponsor, TaMoVac Steering Committee, DSMB and to the ethical committees and regulatory authorities in Tanzania and Mozambique.

Members of the TMG are

Name	***Position***	***Email***
Eligius Lyamuya	Chief Investiagtor, MUHAS	eligius_lyamuya@yahoo.com
Eric Sandström	Protocol co-Chairand SMI's Medical Expert,KI	eric.sandstrom@sodersjukhuset.se
Kisali Pallangyo	MUHAS's Medical Expert	kpallangyo@gmail.com
Arne Kroidl	Protocol co-Chair, LMU	akroidl@mmrp.org
Sheena McCormack	Protocol co-Chair, MRC CTU	TaMoVac@ctu.mrc.ac.uk
Muhammad Bakari	Site PI, MUHAS	drbakari@yahoo.com
Leonard Maboko	Site PI, MMRC	lmaboko@mmrp.org
Ilesh Jani	Site PI, INS/CISPOC	Ilesh.jani@gmail.com
Bahati Kaluwa	Co-PI, MMRC	bkaluwa@mmrp.org
Michael Hoelscher	Senior investigator, LMU	hoelscher@lrz.uni-muenchen.de
Merlin Robb	Consultant for MVA, WRAIR	mrobb@hivresearch.org
Britta Wahren	Consultant for DNA, SMI	britta.wahren@smi.se

Patricia Munseri	Site CRC, MUHAS	pmunseri@yahoo.com
Marco Missanga	Site co-CRC, MMRC	mmissanga@mmrp.org
Philipp Mann	Site co-CRC, MMRC	pmann@mmrp.org
Edna Viegas	Site CRC, CISPOC	edna_viegas@hotmail.com
Nilesh Bhatt	Senior investigator, CISPOC	nbhatt.mz@gmail.com
Gunnel Biberfeld	Consultant for Immunology, KI/SMI	gunnel.biberfeld@smi.se
Candida Moshiro	Trial Statistician	cmoshiro@yahoo.com
Charlotta Nilsson	Immunologist, SMI	charlotta.nilsson@smi.se
Christof Geldmacher	Immunologist, LMU	geldmacher@ lrz.uni-muenchen.de
Sayoki Mfinanga	Consultant for Database, NIMR Muhimbili	gsmfinanga@yahoo.com
Gail Smith	External monitor, WRAIR	gsmith@hivresearch.org
Sue Fleck	Data management mentor, MRC CTU	slf@ctu.mrc.ac.uk

15.3 TaMoVac Steering Committee

The TaMoVac Steering Committee will be chaired by the Director General, Tanzania Commission for Science and Technology (or by MUHAS's Medical Expert if the substantive Chair is absent) and include one representativefrom each the sponsors, the collaborating institutions and WRAIR. The Steering Committee will meet during regular conference calls as well as during the annual investigator meetings. The Steering Committee will form the Scientific Advisory Board overseeing the scientific rational and further developments of the study and study products, as well as other projects undertaken in the TaMoVac network. The Steering Committee may initiate interim analyses provided blinding is maintained and will lead the final analysis of trial results. Presentations and publications of study data will be coordinated and approved by this body. The Steering Committee will lead the communications, financial and progress reporting to the funding bodies and will decide on financial and project management issues. The Steering Committee will receive notes and updates from the TMG concerning milestones and relevant reports of the trial. The committee will request continuous progress reports from the TMG/site PIs to be submitted to the Tanzanian and Mozambican ethical committees and TFDA and the Mozambican Regulatory Authority.

15.4 Community Advisory Board (CAB)

At each study site there will be a Community Advisory Board, drawing representative members from the Police Force and general population in Dar es Salaam and representative members from the generalpopulation in Mbeya and in Maputo. There may also be members from the media, legal representation and study participants. In Mbeya a CAB has been established since 2005 and regular meetings are ongoing since then concerning current and past research activities. In Dar es Salaam and Maputo the CAB will meet before the onset of the study for a general study information session, discussion of recruitment strategies, outreach activities and tracing, participant identification, insurance, and compensation issues. The CAB will be informed in detail about benefits and risks concerning study participation, informed consent procedures as well as HIV risk questionnaires. The purpose of this meeting is also to receive comments about the comprehensibility and clarity of the proposed participant study information/briefing sessions informed consent and recruitment procedures. Regular CAB meetings will then be held at 3 monthly intervals throughout the trial. Up-dates or changes of the study status and information of general HIV vaccine related issues will be provided for the CAB members. Problems concerning individual study participants also in respect to community related topics (e.g. stigmatization) will be

discussed in an anonymous fashion and additional CAB meetings might be called at any time in the case of special circumstances.

15.5 Data and Safety Monitoring Board (DSMB)

An independent Data and Safety Monitoring Board will be appointed by the sponsor of the study, MUHAS and SMI. The role of the DSMB will include:

1. To review the research design and the plans for data and safety monitoring
2. To receive and review at intervals the progress reports from the investigators
3. To monitor safety data
4. To monitor and evaluate trial progress, including
    a. Periodic assessment of data quality and timelines
    b. Performance of the study sites
5. To recommend to the TMG, Sponsor, Tanzanian and Mozambican ethical committees and regulatory authorities concerning continuation or termination of the study.

The DSMB will meet:

- Whenever a safety report has been submitted and to provide recommendation to the TMG, Sponsor, Tanzanian ethical committees and TFDA, Mozambican ethical committee and regulatory authority within 7 days as to the future conduct of the trial.
- If three or more participants experience an unexplained, unexpected grade 3 or 4 clinical or laboratory event (confirmed on repeat testing) not resolved within 72 hours and considered probably or possible and definitely to be related to vaccine product.
- On one occasion to review the safety data after half the volunteers have received the first immunisation in order to make recommendations to the sponsors of the study.

The DSMB will be chaired by:

- Innocent Semali; Co-Chair of DSMB (Tanzania); isemali@muhas.ac.tz
- Esperança Sevene; Co-Chair of DSMB (Mozambique); esevene68@gmail.com

16 PUBLICATION

It is intended that the results of this study will be published in an appropriate peer-reviewed journal, with the aim of submitting a paper for publication within 6 months of the study's completion.

The TaMoVac Steering Committee, the Sponsor, and partners supplying products and devices will have 60 days to comment on any manuscript. No other publications, whether in writing or verbally, will be made before the definitive manuscript has been agreed and accepted for publication, without the prior approval of this committee. A final report of the study will be prepared by the Investigators and circulated to the Steering Committee for comments.
The presenting author for the first conference presentation of the main trial results will be one of the Tanzanian principal investigators or their deputy.

Apart from the international publication(s), results from the trial will be presented at various national and international conferences. Regular updates will be provided to the study participants, relevant local authorities as well as local media for wider dissemination.

In addition, the wider Tanzanian community has, since the first high profile National HIV Vaccine Strategy Plan Workshop in 2001, been informed through the mass media on the need and on plans for HIV vaccine trials in Tanzania. In Mozambique, the first HIV Vaccine Trial (TaMoVac I) was announced by the Minister of Health in September 2011 and the MOH will continue to take a principal role in the spread of information regarding HIV vaccines. The public will continue to be informed through press releases and through other channels on all the stages of the TaMoVac trial.

17 PROTOCOL AMENDMENTS

The TMG will be responsible for preparing protocol amendments. Amendments to the protocol will be made only after consultation and agreement between sponsors and Principal investigators. The only exception is where any of the site PIs considers that a volunteer's safety is compromised without immediate action. All amendments that have an impact on volunteer risk or the study objectives, or require revision of the informed consent document, must receive approval from the relevant ethical committees prior to their implementation.

18 REFERENCES

1. IAVI, Estmating the global impact of an AIDS vaccine. IAVI policy brief, 2006.
2. Tanzania commission for AIDS (TACAIDS), Z.A.c.Z., National bureau of statistics. Tanzanian HIV/AIDS and malaria indicator survey. 2008 [cited; Available from: http://www.ihi.or.tz/docs/TzHIV-malariaindicatorsurvey-07-08-preliminaryreport.pdf.
3. Matee, M.I., et al., Prevalence of transfusion-associated viral infections and syphilis among blood donors in Muhimbili Medical Centre, Dar es Salaam, Tanzania. East Afr Med J, 1999. **76**(3): p. 167-71.
4. Hoelscher, M., et al., High proportion of unrelated HIV-1 intersubtype recombinants in the Mbeya region of southwest Tanzania. Aids, 2001. **15**(12): p. 1461-70.
5. Kiwelu, I.E., et al., HIV type 1 subtypes among bar and hotel workers in Moshi, Tanzania. AIDS Res Hum Retroviruses, 2003. **19**(1): p. 57-64.
6. Lyamuya, E., et al., Evaluation of a prototype Amplicor PCR assay for detection of human immunodeficiency virus type 1 DNA in blood samples from Tanzanian adults infected with HIV-1 subtypes A, C and D. J Clin Virol, 2000. **17**(1): p. 57-63.
7. Renjifo, B., et al., Epidemic expansion of HIV type 1 subtype C and recombinant genotypes in Tanzania.AIDS Res Hum Retroviruses, 1998. **14**(7): p. 635-8.
8. Inquérito Nacional de Prevalência, Riscos comportamentais, e Informação sobre HIV e SIDA em Moçambique (INSIDA), Relatório Preliminar, INS 2010
9. Walker, B.D. and D.R. Burton, Toward an AIDS vaccine. Science, 2008. **320**(5877): p. 760-4.
10. Barouch, D.H., Challenges in the development of an HIV-1 vaccine. Nature, 2008. **455**(7213): p. 613-9.
11. Sodora, D.L., et al., Toward an AIDS vaccine: lessons from natural simian immunodeficiency virus infections of African nonhuman primate hosts. Nat Med, 2009. **15**(8): p. 861-5.
12. Johnston, M.I. and A.S. Fauci, An HIV vaccine--challenges and prospects. N Engl J Med, 2008. **359**(9): p. 888-90.
13. Kaufman, D.R. and D.H. Barouch, Translational Mini-Review Series on Vaccines for HIV: T lymphocyte trafficking and vaccine-elicited mucosal immunity. Clin Exp Immunol, 2009. **157**(2): p. 165-73.
14. Franchini, G., Choosing the right memory T cell for HIV. Nat Med, 2009. **15**(3): p. 244-6.
15. Masopust, D., Developing an HIV cytotoxic T-lymphocyte vaccine: issues of CD8 T-cell quantity, quality and location. J Intern Med, 2009. **265**(1): p. 125-37.
16. Coutsinos, Z., et al., [Designing an effective AIDS vaccine: strategies and current status]. Rev Med Interne, 2008. **29**(8): p. 632-41.
17. Rerks-Ngarm, S., et al., Vaccination with ALVAC and AIDSVAX to prevent HIV-1 infection in Thailand. N Engl J Med, 2009. **361**(23): p. 2209-20.
18. Haynes, B.F., Case-control study of the RV144 for immune correlates:the analysis and way forward. AIDS vaccine 2011, 12015 September 2011, Bangkok, Thailand [Abstract PL01.04], 2011.
19. McMichael, A.J., et al., The immune response during acute HIV-1 infection: clues for vaccine development. Nat Rev Immunol, 2010. **10**(1): p. 11-23.
20. McElrath, M.J. and B.F. Haynes, Induction of immunity to human immunodeficiency virus type-1 by vaccination. Immunity, 2011. **33**(4): p. 542-54.
21. Woodland, D.L., Jump-starting the immune system: prime-boosting comes of age. Trends Immunol, 2004. **25**(2): p. 98-104.
22. Robinson, H.L. and R.R. Amara, T cell vaccines for microbial infections. Nat Med, 2005. **11**(4 Suppl): p. S25-32.

23. Letvin, N.L., et al., Preserved CD4+ central memory T cells and survival in vaccinated SIV-challenged monkeys. Science, 2006. **312**(5779): p. 1530-3.
24. Makitalo, B., et al., Enhanced cellular immunity and systemic control of SHIV infection by combined parenteral and mucosal administration of a DNA prime MVA boost vaccine regimen. J Gen Virol, 2004. **85**(Pt 8): p. 2407-19.
25. Kim, J.H., et al., HIV vaccines: lessons learned and the way forward. Curr Opin HIV AIDS, 2010. **5**(5): p. 428-34.
26. McCormack, S., et al., EV02: a Phase I trial to compare the safety and immunogenicity of HIV DNA-C prime-NYVAC-C boost to NYVAC-C alone. Vaccine, 2008. **26**(25): p. 3162-74.
27. Sandstrom, E., et al., Broad immunogenicity of a multigene, multiclade HIV-1 DNA vaccine boosted with heterologous HIV-1 recombinant modified vaccinia virus Ankara. J Infect Dis, 2008. **198**(10): p. 1482-90.
28. Excler, J.L., et al., A strategy for accelerating the development of preventive AIDS vaccines. Aids, 2007. **21**(17): p. 2259-63.
29. Bakari, M., et al., Broad and potent immune responses to a low dose intradermal HIV-1 DNA boosted with HIV-1 recombinant MVA among healthy adults in Tanzania. Vaccine, 2011. **29**(46): p. 8417-28.
30. Paris, R.M., et al., Prime-boost immunization with poxvirus or adenovirus vectors as a strategy to develop a protective vaccine for HIV-1. Expert Rev Vaccines, 2010. **9**(9): p. 1055-69.
31. Harari, A., et al., An HIV-1 clade C DNA prime, NYVAC boost vaccine regimen induces reliable, polyfunctional, and long-lasting T cell responses. J Exp Med, 2008. **205**(1): p. 63-77.
32. Levy, Y., et al., Optimal priming of poxvirus vector-based regimens requires 3 DNA injections; results of the randomised multicentre EV03/ANRS Vac20 Phase I/II trial., in 17th Conference on Retroviruses and Opportunisitic Infections (78LB). 2010.
33. Zaharoff, D.A., et al., Electromobility of plasmid DNA in tumor tissues during electric field-mediated gene delivery. Gene Ther, 2002. **9**(19): p. 1286-90.
34. Prud'homme, G.J., et al., Electroporation-enhanced nonviral gene transfer for the prevention or treatment of immunological, endocrine and neoplastic diseases. Curr Gene Ther, 2006. **6**(2): p. 243-73.
35. Aihara, H. and J. Miyazaki, Gene transfer into muscle by electroporation in vivo. Nat Biotechnol, 1998. **16**(9): p. 867-70.
36. Otten, G.R., et al., Potent immunogenicity of an HIV-1 gag-pol fusion DNA vaccine delivered by in vivo electroporation. Vaccine, 2006. **24**(21): p. 4503-9.
37. Mir, L.M., et al., Long-term, high level in vivo gene expression after electric pulse-mediated gene transfer into skeletal muscle. C R Acad Sci III, 1998. **321**(11): p. 893-9.
38. Mir, L.M., et al., High-efficiency gene transfer into skeletal muscle mediated by electric pulses. Proc Natl Acad Sci U S A, 1999. **96**(8): p. 4262-7.
39. Miyazaki, J. and H. Aihara, Gene transfer into muscle by electroporation in vivo. Methods Mol Med, 2002. **69**: p. 49-62.
40. Vasan, S., et al., In vivo electroporation enhances the immunogenicity of an HIV-1 DNA vaccine candidate in healthy volunteers. PLoS One, 2011. **6**(5): p. e19252.
41. Brave, A., et al., A new multi-clade DNA prime/recombinant MVA boost vaccine induces broad and high levels of HIV-1-specific CD8(+) T-cell and humoral responses in mice. Mol Ther, 2007. **15**(9): p. 1724-33.
42. Sheets, R.L., et al., Biodistribution of DNA plasmid vaccines against HIV-1, Ebola, Severe Acute Respiratory Syndrome, or West Nile virus is similar, without integration, despite differing plasmid backbones or gene inserts. Toxicol Sci, 2006. **91**(2): p. 610-9.
43. Loutfy, M.R., et al., A large prospective study assessing injection site reactions, quality of life and preference in patients using the Biojector vs standard needles for enfuvirtide administration. HIV Med, 2007. **8**(7): p. 427-32.

44. Ljungberg, K., et al., Enhanced immune responses after DNA vaccination with combined envelope genes from different HIV-1 subtypes. Virology, 2002. **302**(1): p. 44-57.
45. Rollman, E., et al., The rationale behind a vaccine based on multiple HIV antigens. Microbes Infect, 2005. **7**(14): p. 1414-23.
46. Wahren, B., et al., HIV subtypes and recombination strains--strategies for induction of immune responses in man. Vaccine, 2002. **20**(15): p. 1988-93.
47. Dorrell, L., et al., Safety and tolerability of recombinant modified vaccinia virus Ankara expressing an HIV-1 gag/multiepitope immunogen (MVA.HIVA) in HIV-1-infected persons receiving combination antiretroviral therapy. Vaccine, 2007. **25**(17): p. 3277-83.
48. Mulligan, M.J., et al., Excellent safety and tolerability of the human immunodeficiency virus type 1 pGA2/JS2 plasmid DNA priming vector vaccine in HIV type 1 uninfected adults. AIDS Res Hum Retroviruses, 2006. **22**(7): p. 678-83.

APPENDIX 1: STUDY TIMETABLE

Target day	Week	Visit	Visit/Assessment type	Visit Window
	-4--8	V1	Screening I	4-8 weeks before V3 enrolment
	-2--4	V2	Screening II + baseline immunogenicity I	2-4 weeks before V3 enrolment
0	0	V3	Enrolment + DNA I + baseline immunogenicity II	Week 0
0		V3A	30 min post vaccination	
0		V3B	Evening of vaccination day	
1		V3C	Day 1 post vaccination	
2		V3D	Day 2 post vaccination	
3		V3E	Day 3 post vaccination	
4		V3F	Day 4 post vaccination	
5		V3G	Day 5 post vaccination	
6		V3H	Day 6 post vaccination	
7		V3I	Day 7 post vaccination	
14	2	V4	Follow-up safety (+ immunogenicity)	14 days post V3 +/- 3 days
28	4	V5	DNA II + Follow-up safety	28 days post V3 +/- 3 days
28		V5A	30 min post vaccination	
28		V5B	Evening of vaccination day	
29		V5C	Day 1 post vaccination	
30		V5D	Day 2 post vaccination	
31		V5E	Day 3 post vaccination	
32		V5F	Day 4 post vaccination	
33		V5G	Day 5 post vaccination	
34		V5H	Day 6 post vaccination	
35		V5I	Day 7 post vaccination	
42	6	V6	Follow-up safety + immunogenicity	14 days post V5 +/- 3 days
56	8	V7	Follow-up safety	28 days post V5 +/- 5 days
84	12	V8	DNA III	84 days post V3 +/- 7 days
84		V8A	30 min post vaccination	
84		V8B	Evening of vaccination day	
85		V8C	Day 1 post vaccination	
86		V8D	Day 2 post vaccination	
87		V8E	Day 3 post vaccination	
88		V8F	Day 4 post vaccination	
89		V8G	Day 5 post vaccination	
90		V8H	Day 6 post vaccination	
91		V8I	Day 7 post vaccination	
98	14	V9	Follow-up safety + immunogenicity	14 days post V8 +/- 3 days
112	16	V10	Follow-up safety	28 days post V8 +/- 5 days
168	24	V11	MVA I +/- CN54rgp140+ GLA-AF + immunogenicity	84 days post V8 +/-7 days
168		V11A	30 min post vaccination	
168		V11B	Evening of vaccination day	
169		V11C	Day 1 post vaccination	
170		V11D	Day 2 post vaccination	
171		V11E	Day 3 post vaccination	
172		V11F	Day 4 post vaccination	
173		V11G	Day 5 post vaccination	
174		V11H	Day 6 post vaccination	

175		V11I	Day 7 post vaccination	
182	26	V12	Follow-up safety + immunogenicity	14 days post V11 +/- 3 days
196	28	V13	Follow-up safety	28 days post V11 +/- 5 days
280	40	V14	MVA II +/- CN54rgp140 + GLA-AF + immunogenicity	112 days post V11 +/-14 days
280		V14A	30 min post vaccination	
280		V14B	Evening of vaccination day	
281		V14C	Day 1 post vaccination	
282		V14D	Day 2 post vaccination	
283		V14E	Day 3 post vaccination	
284		V14F	Day 4 post vaccination	
285		V14G	Day 5 post vaccination	
286		V14H	Day 6 post vaccination	
287		V14I	Day 7 post vaccination	
294	42	V15	Follow-up safety + immunogenicity	14 days post V14 +/- 3 days
308	44	V16	Follow-up safety + immunogenicity	28 days post V14 +/- 5 days
364	52	V17	Follow-up immunogenicity	84 days post V14 +/- 14 days
448	64	V18	Follow-up immunogenicity	168 days post V14 +/- 14 days

APPENDIX 2: TAMOVAC II SCHEDULE OF EVENTS& LAB FLOW CHART

		**Weeks**	**-4-8**	**-1-4**	**0**	**2**	**4**	**6**	**8**	**12**	**14**	**16**	**24**	**26**	**28**	**40**	**42**	**44**	**52**	**64**
		**Target days**			**0**	**14**	**28**	**42**	**56**	**84**	**98**	**112**	**168**	**182**	**196**	**280**	**294**	**308**	**364**	**448**
		**Visits**	**V1**	**V2**	**V3**	**V4**	**V5**	**V6**	**V7**	**V8**	**V9**	**V10**	**V11**	**V12**	**V13**	**V14**	**V15**	**V16**	**V17**	**V18**
			**Screening 1**	**Screening 2**	**Enrolment + DNA 1**	**Safety FU**	**DNA 2**	**Safety FU**	**Safety FU**	**DNA 3**	**Safety FU**	**Safety FU**	**MVA 1+/- rgp140/GLA**	**Safety FU**	**Safety FU**	**MVA 2+/- rgp140/GLA**	**Safety FU**	**Safety FU**	**Long term FU**	**Long term FU**
**Clinical Procedures**																				
Informed Consent			x																	
Test of Understanding			x																	
HIV Risk Assessment			x																	
Medical History			x	x	x	x	x	x	x	x	x	x	x	x	x	x	x	x	x	x
Complete Physical Exam			x																	
Directed Physical Exam				x	x	x	x	x	x	x	x	x	x	x	x	x	x	x	x	x
Immunization					x		x			x			x			x				
Adverse Events assessments						x	x	x	x	x	x	x	x	x	x	x	x	x	x	x
Diary Card Revision						x		x			x			x			x			
ECG			X*		(x)									(x)			(x)			
**Lab Investigations**	**Amount (ml)**	**Type and # of tubes**																		
**Haematology (H)**																				
CBC	3 ml	Purple top EDTA	6ml		6ml	6ml	6ml	6ml	6ml	6ml	6ml	6ml	6ml	6ml	6ml	6ml	6ml	6ml	6ml	6ml
**Biochemistry (BC)**																				
ALT, bilirubin, creatinine, glucose	6 ml	Red top coagulant	6ml		6ml	6ml	6ml	6ml	6ml	6ml	6ml	6ml	6ml	6ml	6ml	6ml	6ml	6ml	6ml	6ml
Troponin I	From BC													(x)**			(x)**			
**Serology**																				
HbsAg, Syphillis	From BC	Red top coagulant	from BC																	
**HIV test algorithm**																				
HIV ELISA/WB/PCR	From H	Purple top EDTA	from H		from H		from H			from H			from H			from H			from H	from H

*Screening ECG can be performed on visit 1 or 2
**Troponin I test can be performed after MVA vaccination if clinicaly indicated

		Weeks	-4-8	-1-4	0	2	4	6	8	12	14	16	24	26	28	40	42	44	52	64
		**Target days**			**0**	14	**28**	**42**	**56**	**84**	**98**	**112**	**168**	**182**	**196**	**280**	**294**	**308**	**364**	**448**
		**Visits**	**V1**	**V2**	**V3**	**V4**	**V5**	**V6**	**V7**	**V8**	**V9**	**V10**	**V11**	**V12**	**V13**	**V14**	**V15**	**V16**	**V17**	**V18**
**Lab Investigations**	**Amount (ml)**	**Type and # of tubes**	**Screening 1**	**Screening 2**	**Enrolment + DNA 1**	**Safety FU**	**DNA 2**	**Safety FU**	**Safety FU**	**DNA 3**	**Safety FU**	**Safety FU**	**MVA 1 +/- rgp140/GLA**	**Safety FU**	**Safety FU**	**MVA 2+/- rgp140/GLA**	**Safety FU**	**Safety FU**	**Long term FU**	**Long term FU**
**Immunological tests**																				
CD4 count	From BC	Purple top EDTA			from H															
HLA typing	8 ml	Purple top EDTA			8ml															
**Immunogenicity tests**																				
Binding and neutralizing Ab.incl. Vaccinia Ab.	1x20ml	Red top coagulant		X	X						X			X	X		X	X	X	X
IFN-gamma ELISPOT, fresh cells					**X**						**X**			**X**			**X**		**(X)#**	**X**
ICS, fresh cells (optional)					**X**						**X**			**X**			**X**		**(X)#**	**X**
Lymphocyte proliferation assay, fresh cells (optional)	9x10ml	Green top Natrium Heparin			**X**						**X**			**X**			**X**		**(X)#**	**X**
ICS multi-colors, cryo-preserved cells				X	X						X			X	X		X	X	(X)#	X
Optional assays, cryo-preserved cells				X	X						X			X	X		X	X	(X)#	X
**Total volume for immunogenicity testing:**				**110**	**110**						**110**			**110**	**110**		**110**	**110**	**20**	**110**
**Total blood volume = 1112ml**			**12**	**110**	**130**	**12**	**12**	**12**	**12**	**12**	**122**	**12**	**12**	**122**	**122**	**12**	**122**	**122**	**32**	**122**
**Urine**																				
Urine for pregnancy test, urine dipstick	5 ml	Sterile urine container	x		x		x			x			x			x			x	x

CBC – complete blood count, ICS - intracellular cytokine staining; CTL - cytotoxic lymphocyte assay; Red top=coagulant; Purple top=EDTA; Yellow top=acid citrate dextrose.
Stored samples will be used for antibody testing and stored PBMC will be cryo-preserved for cellular immunogenicity tests. Immunogenicity tests using fresh cells will performed where marked in ***bold.*** Plasma from EDTA and PBMC processing will be stored for contingency purposes and immunogenicity tests.
Large blood volume draw for immunogenicity testing at Visit 17 will only be performed if Visit 18 blood draws are not likely to take place (e.g. early termination). Large blood draws for immunogenicity testing will therefore be performed ***either*** at Visit 17 ***or*** at Visit 18.

APPENDIX 3A1: TAMOVAC II INFORMATION SHEET (ENGLISH) TANZANIA

APPENDIX 3A2: TAMOVAC II INFORMATION SHEET (ENGLISH) MOZAMBIQUE

APPENDIX 3B: TAMOVAC II INFORMATION SHEET (SWAHILI)

APPENDIX 3C: TAMOVAC II INFORMATION SHEET (PORTUGUESE)

APPENDIX 4A: TAMOVAC II ASSESSMENT OF UNDERSTANDING (ENGLISH)

APPENDIX 4B: TAMOVAC II ASSESSMENT OF UNDERSTANDING (SWAHILI)

APPENDIX 4C: TAMOVAC II ASSESSMENT OF UNDERSTANDING (PORTUGUESE)

APPENDIX 5A: TAMOVAC II RISK ASSESSMENT OF VOLUNTEERS (ENGLISH)

APPENDIX 5B: TAMOVAC II RISK ASSESSMENT OF VOLUNTEERS (SWAHILI)

APPENDIX 5C: TAMOVAC IIRISK ASSESSMENT OF VOLUNTEERS (PORTUGUESE)

APPENDIX 6A: TAMOVAC II DIARY CARD (ENGLISH)

APPENDIX 6B: TAMOVAC II DIARY CARD (SWAHILI)

APPENDIX 6C: TAMOVAC II DIARY CARD (PORTUGUESE)

APPENDIX 7A: TAMOVAC II CERTIFICATE ON HIV VACCINE TRIAL PARTICIPATION (ENGLISH)

APPENDIX 7B: TAMOVAC II CERTIFICATE ON HIV VACCINE TRIAL PARTICIPATION (SWAHILI)

APPENDIX 7C: TAMOVAC II CERTIFICATE ON HIV VACCINE TRIAL PARTICIPATION (PORTUGUESE)

APPENDIX 8: DAIDS TABLE FOR CLINICAL AND LAB CRITERIA

Division of AIDS table for grading the severity of adult and pediatric adverse events version 1.0, December 2004, clarification August 2009.

(http://rsc.techres.com/Document/safetyandpharmacovigilance/Table_for_Grading_Severity_of_Adult_Pediatric_Adverse_Events.pdf)

CLINICAL				
**PARAMETER**	**GRADE 1 MILD**	**GRADE 2 MODERATE**	**GRADE 3 SEVERE**	**GRADE 4 POTENTIALLY LIFE-THREATENING**
**ESTIMATING SEVERITY GRADE**				
Clinical adverse event NOT identified elsewhere in this DAIDS AE grading table	Symptoms causing no or minimal interference with usual social & functional activities	Symptoms causing greater than minimal interference with usual social & functional activities	Symptoms causing inability to perform usual social & functional activities	Symptoms causing inability to perform basic self-care functions OR Medical or operative intervention indicated to prevent permanent impairment, persistent disability, or death
**SYSTEMIC**				
Acute systemic allergic reaction	Localized urticaria (wheals) with no medical intervention indicated	Localized urticaria with medical intervention indicated OR Mild angioedema with no medical intervention indicated	Generalized urticaria OR Angioedema with medical intervention indicated OR Symptomatic mild bronchospasm	Acute anaphylaxis OR Life-threatening bronchospasm OR laryngeal edema
Chills	Symptoms causing no or minimal interference with usual social & functional activities	Symptoms causing greater than minimal interference with usual social & functional activities	Symptoms causing inability to perform usual social & functional activities	NA
Fatigue<br>Malaise	Symptoms causing no or minimal interference with usual social & functional activities	Symptoms causing greater than minimal interference with usual social & functional activities	Symptoms causing inability to perform usual social & functional activities	Incapacitating fatigue/ malaise symptoms causing inability to perform basic self-care functions
Fever (nonaxillary)	37.7 – 38.6°C	38.7 – 39.3°C	39.4 – 40.5°C	> 40.5°C
Pain (indicate body site)<br>DO NOT use for pain due to injection (See Injection Site Reactions: Injection site pain)<br>See also Headache, Arthralgia, and Myalgia	Pain causing no or minimal interference with usual social & functional activities	Pain causing greater than minimal interference with usual social & functional activities	Pain causing inability to perform usual social & functional activities	Disabling pain causing inability to perform basic self-care functions OR Hospitalization (other than emergency room visit) indicated
Unintentional weight loss	NA	5 – 9% loss in body weight from baseline	10 – 19% loss in body weight from baseline	≥ 20% loss in body weight from baseline OR Aggressive intervention indicated [e.g., tube feeding or total parenteral nutrition (TPN)]

CLINICAL				
**PARAMETER**	**GRADE 1 MILD**	**GRADE 2 MODERATE**	**GRADE 3 SEVERE**	**GRADE 4 POTENTIALLY LIFE-THREATENING**
**INFECTION**				
Infection (any other than HIV infection)	Localized, no systemic antimicrobial treatment indicated AND Symptoms causing no or minimal interference with usual social & functional activities	Systemic antimicrobial treatment indicated OR Symptoms causing greater than minimal interference with usual social & functional activities	Systemic antimicrobial treatment indicated AND Symptoms causing inability to perform usual social & functional activities OR Operative intervention (other than simple incision and drainage) indicated	Life-threatening consequences (e.g., septic shock)
**INJECTION SITE REACTIONS**				
Injection site pain (pain without touching) Or Tenderness (pain when area is touched)	Pain/tenderness causing no or minimal limitation of use of limb	Pain/tenderness limiting use of limb OR Pain/tenderness causing greater than minimal interference with usual social & functional activities	Pain/tenderness causing inability to perform usual social & functional activities	Pain/tenderness causing inability to perform basic self-care function OR Hospitalization (other than emergency room visit) indicated for management of pain/tenderness
Injection site reaction (localized)				
**Adult > 15 years**	Erythema OR Induration of 5x5 cm – 9x9 cm (or 25 cm^2 – 81cm^2)	Erythema OR Induration OREdema > 9 cm any diameter (or > 81 cm^2)	Ulceration OR Secondary infection OR Phlebitis OR Sterile abscess OR Drainage	Necrosis (involving dermis and deeper tissue)
**Pediatric ≤ 15 years**	Erythema OR Induration OR Edema present but ≤ 2.5 cm diameter	Erythema OR Induration OR Edema > 2.5 cm diameter but < 50% surface area of the extremity segment (e.g., upper arm/thigh)	Erythema OR Induration OR Edema involving ≥ 50% surface area of the extremity segment (e.g., upper arm/thigh) OR Ulceration OR Secondary infection OR Phlebitis OR Sterile abscess OR Drainage	Necrosis (involving dermis and deeper tissue)
Pruritis associated with injection See also Skin: Pruritis (itching - no skin lesions)	Itching localized to injection site AND Relieved spontaneously or with < 48 hours treatment	Itching beyond the injection site but not generalized OR Itching localized to injection site requiring ≥ 48 hours treatment	Generalized itching causing inability to perform usual social & functional activities	NA
**SKIN – DERMATOLOGICAL**				
Alopecia	Thinning detectable by study participant (or by caregiver for young children and disabled adults)	Thinning or patchy hair loss detectable by health care provider	Complete hair loss	NA
Cutaneous reaction – rash	Localized macular rash	Diffuse macular, maculopapular, or morbilliform rash OR Target lesions	Diffuse macular, maculopapular, or morbilliform rash with vesicles or limited number of bullae OR Superficial ulcerations of mucous membrane limited to one site	Extensive or generalized bullous lesions OR Stevens-Johnson syndrome OR Ulceration of mucous membrane involving two or more distinct mucosal sites OR Toxic epidermal

CLINICAL				
**PARAMETER**	**GRADE 1 MILD**	**GRADE 2 MODERATE**	**GRADE 3 SEVERE**	**GRADE 4 POTENTIALLY LIFE-THREATENING**
				necrolysis (TEN)
Hyperpigmentation	Slight or localized	Marked or generalized	NA	NA
Hypopigmentation	Slight or localized	Marked or generalized	NA	NA
Pruritis (itching – no skin lesions)<br>(See also Injection Site Reactions: Pruritis associated with injection)	Itching causing no or minimal interference with usual social & functional activities	Itching causing greater than minimal interference with usual social & functional activities	Itching causing inability to perform usual social & functional activities	NA
**CARDIOVASCULAR**				
Cardiac arrhythmia (general)<br>(By ECG or physical exam)	Asymptomatic AND No intervention indicated	Asymptomatic AND Non-urgent medical intervention indicated	Symptomatic, non-life-threatening AND Non-urgent medical intervention indicated	Life-threatening arrhythmia OR Urgent intervention indicated
Cardiac-ischemia/infarction	NA	NA	Symptomatic ischemia (stable angina) OR Testing consistent with ischemia	Unstable angina OR Acute myocardial infarction
Hemorrhage (significant acute blood loss)	NA	Symptomatic AND No transfusion indicated	Symptomatic AND Transfusion of ≤ 2 units packed RBCs (for children ≤ 10 cc/kg) indicated	Life-threatening hypotension OR Transfusion of > 2 units packed RBCs (for children > 10 cc/kg) indicated
Hypertension				
**Adult > 17 years**<br>(with repeat testing at same visit)	> 140 – 159 mmHg systolic<br>OR<br>> 90 – 99 mmHg diastolic	> 160 – 179 mmHg systolic<br>OR<br>> 100 – 109 mmHg diastolic	> 180 mmHg systolic<br>OR<br>> 110 mmHg diastolic	Life-threatening consequences (e.g., malignant hypertension) OR Hospitalization indicated (other than emergency room visit)
**Pediatric ≤ 17 years**<br>(with repeat testing at same visit)	NA	91st – 94th percentile adjusted for age, height, and gender (systolic and/or diastolic)	≥ 95th percentile adjusted for age, height, and gender (systolic and/or diastolic)	Life-threatening consequences (e.g., malignant hypertension) OR Hospitalization indicated (other than emergency room visit)
Hypotension	NA	Symptomatic, corrected with oral fluid replacement	Symptomatic, IV fluids indicated	Shock requiring use of vasopressors or mechanical assistance to maintain blood pressure
Pericardial effusion	Asymptomatic, small effusion requiring no intervention	Asymptomatic, moderate or larger effusion requiring no intervention	Effusion with non-life threatening physiologic consequences OR Effusion with non-urgent intervention indicated	Life-threatening consequences (e.g., tamponade) OR Urgent intervention indicated
Prolonged PR interval				
**Adult > 16 years**	PR interval 0.21 – 0.25 sec	PR interval > 0.25 sec	Type II 2nd degree AV block OR Ventricular	Complete AV block

CLINICAL					
PARAMETER		GRADE 1<br>MILD	GRADE 2<br>MODERATE	GRADE 3<br>SEVERE	GRADE 4<br>POTENTIALLY<br>LIFE-THREATENING
				pause > 3.0 sec	
	**Pediatric ≤ 16 years**	1st degree AV block (PR > normal for age and rate)	Type I 2nd degree AV block	Type II 2nd degree AV block	Complete AV block
Prolonged QTc					
	**Adult > 16 years**	Asymptomatic, QTc interval 0.45 – 0.47 sec OR Increase interval < 0.03 sec above baseline	Asymptomatic, QTc interval 0.48 – 0.49 sec OR Increase in interval 0.03 – 0.05 sec above baseline	Asymptomatic, QTc interval ≥ 0.50 sec OR Increase in interval ≥ 0.06 sec above baseline	Life-threatening consequences, e.g. Torsade de pointes or other associated serious ventricular dysrhythmia
	**Pediatric ≤ 16 years**	Asymptomatic, QTc interval 0.450 – 0.464 sec	Asymptomatic, QTc interval 0.465 – 0.479 sec	Asymptomatic, QTc interval ≥ 0.480 sec	Life-threatening consequences, e.g. Torsade de pointes or other associated serious ventricular dysrhythmia
Thrombosis/embolism		NA	Deep vein thrombosis AND No intervention indicated (e.g., anticoagulation, lysis filter, invasive procedure)	Deep vein thrombosis AND Intervention indicated (e.g., anticoagulation, lysis filter, invasive procedure)	Embolic event (e.g., pulmonary embolism, life-threatening thrombus)
Vasovagal episode (associated with a procedure of any kind)		Present without loss of consciousness	Present with transient loss of consciousness	NA	NA
Ventricular dysfunction (congestive heart failure)		NA	Asymptomatic diagnostic finding AND intervention indicated	New onset with symptoms OR Worsening symptomatic congestive heart failure	Life-threatening congestive heart failure
**GASTROINTESTINAL**					
Anorexia		Loss of appetite without decreased oral intake	Loss of appetite associated with decreased oral intake without significant weight loss	Loss of appetite associated with significant weight loss	Life-threatening consequences OR Aggressive intervention indicated [e.g., tube feeding or total parenteral nutrition (TPN)]
Ascites		Asymptomatic	Symptomatic AND Intervention indicated (e.g., diuretics or therapeutic paracentesis)	Symptomatic despite intervention	Life-threatening consequences
Cholecystitis		NA	Symptomatic AND Medical intervention indicated	Radiologic, endoscopic, or operative intervention indicated	Life-threatening consequences (e.g., sepsis or perforation)
Constipation		NA	Persistent constipation requiring regular use of dietary modifications, laxatives, or enemas	Obstipation with manual evacuation indicated	Life-threatening consequences (e.g., obstruction)
Diarrhea					
	**Adult and Pediatric ≥ 1 year**	Transient or intermittent episodes of unformed stools	Persistent episodes of unformed to watery stools OR Increase of	Bloody diarrhea OR Increase of ≥ 7 stools per 24-hour period OR	Life-threatening consequences (e.g.,

CLINICAL				
**PARAMETER**	**GRADE 1 MILD**	**GRADE 2 MODERATE**	**GRADE 3 SEVERE**	**GRADE 4 POTENTIALLY LIFE-THREATENING**
	OR Increase of ≤ 3 stools over baseline per 24-hour period	4 – 6 stools over baseline per 24-hour period	IV fluid replacement indicated	hypotensive shock)
**Pediatric < 1 year**	Liquid stools (more unformed than usual) but usual number of stools	Liquid stools with increased number of stools OR Mild dehydration	Liquid stools with moderate dehydration	Liquid stools resulting in severe dehydration with aggressive rehydration indicated OR Hypotensive shock
Dysphagia-Odynophagia	Symptomatic but able to eat usual diet	Symptoms causing altered dietary intake without medical intervention indicated	Symptoms causing severely altered dietary intake with medical intervention indicated	Life-threatening reduction in oral intake
Mucositis/stomatitis (clinical exam)<br>Indicate site (e.g., larynx, oral)<br>See Genitourinary for Vulvovaginitis<br>See also Dysphagia-Odynophagia and Proctitis	Erythema of the mucosa	Patchy pseudomembranes or ulcerations	Confluent pseudomembranes or ulcerations OR Mucosal bleeding with minor trauma	Tissue necrosis OR Diffuse spontaneous mucosal bleeding OR Life-threatening consequences (e.g., aspiration, choking)
Nausea	Transient (< 24 hours) or intermittent nausea with no or minimal interference with oral intake	Persistent nausea resulting in decreased oral intake for 24 – 48 hours	Persistent nausea resulting in minimal oral intake for > 48 hours OR Aggressive rehydration indicated (e.g., IV fluids)	Life-threatening consequences (e.g., hypotensive shock)
Pancreatitis	NA	Symptomatic AND Hospitalization not indicated (other than emergency room visit)	Symptomatic AND Hospitalization indicated (other than emergency room visit)	Life-threatening consequences (e.g., circulatory failure, hemorrhage, sepsis)
Proctitis (functional-symptomatic)<br>Also see Mucositis/stomatitis for clinical exam	Rectal discomfort AND No intervention indicated	Symptoms causing greater than minimal interference with usual social & functional activities OR Medical intervention indicated	Symptoms causing inability to perform usual social & functional activities OR Operative intervention indicated	Life-threatening consequences (e.g., perforation)
Vomiting	Transient or intermittent vomiting with no or minimal interference with oral intake	Frequent episodes of vomiting with no or mild dehydration	Persistent vomiting resulting in orthostatic hypotension OR Aggressive rehydration indicated (e.g., IV fluids)	Life-threatening consequences (e.g., hypotensive shock)
**NEUROLOGIC**				
Alteration in personality-behavior or in mood (e.g., agitation, anxiety, depression, mania, psychosis)	Alteration causing no or minimal interference with usual social & functional activities	Alteration causing greater than minimal interference with usual social & functional activities	Alteration causing inability to perform usual social & functional activities	Behavior potentially harmful to self or others (e.g., suicidal and homicidal ideation or attempt, acute psychosis) OR Causing inability to perform basic self-care functions
Altered Mental Status	Changes causing no or minimal	Mild lethargy or somnolence causing	Confusion, memory impairment, lethargy, or	Delirium OR obtundation,

CLINICAL				
**PARAMETER**	**GRADE 1 MILD**	**GRADE 2 MODERATE**	**GRADE 3 SEVERE**	**GRADE 4 POTENTIALLY LIFE-THREATENING**
For Dementia, see Cognitive and behavioral/attentional disturbance (including dementia and attention deficit disorder)	interference with usual social & functional activities	greater than minimal interference with usual social & functional activities	somnolence causing inability to perform usual social & functional activities	OR coma
Ataxia	Asymptomatic ataxia detectable on exam OR Minimal ataxia causing no or minimal interference with usual social & functional activities	Symptomatic ataxia causing greater than minimal interference with usual social & functional activities	Symptomatic ataxia causing inability to perform usual social & functional activities	Disabling ataxia causing inability to perform basic self-care functions
Cognitive and behavioral/attentional disturbance (including dementia and attention deficit disorder)	Disability causing no or minimal interference with usual social & functional activities OR Specialized resources not indicated	Disability causing greater than minimal interference with usual social & functional activities OR Specialized resources on part-time basis indicated	Disability causing inability to perform usual social & functional activities OR Specialized resources on a full-time basis indicated	Disability causing inability to perform basic self-care functions OR Institutionalization indicated
CNS ischemia (acute)	NA	NA	Transient ischemic attack	Cerebral vascular accident (CVA, stroke) with neurological deficit
Developmental delay – **Pediatric ≤ 16 years**	Mild developmental delay, either motor or cognitive, as determined by comparison with a developmental screening tool appropriate for the setting	Moderate developmental delay, either motor or cognitive, as determined by comparison with a developmental screening tool appropriate for the setting	Severe developmental delay, either motor or cognitive, as determined by comparison with a developmental screening tool appropriate for the setting	Developmental regression, either motor or cognitive, as determined by comparison with a developmental screening tool appropriate for the setting
Headache	Symptoms causing no or minimal interference with usual social & functional activities	Symptoms causing greater than minimal interference with usual social & functional activities	Symptoms causing inability to perform usual social & functional activities	Symptoms causing inability to perform basic self-care functions OR Hospitalization indicated (other than emergency room visit) OR Headache with significant impairment of alertness or other neurologic function
Insomnia	NA	Difficulty sleeping causing greater than minimal interference with usual social & functional activities	Difficulty sleeping causing inability to perform usual social & functional activities	Disabling insomnia causing inability to perform basic self-care functions
Neuromuscular weakness (including myopathy & neuropathy)	Asymptomatic with decreased strength on exam OR Minimal muscle weakness causing no or minimal interference with usual social & functional activities	Muscle weakness causing greater than minimal interference with usual social & functional activities	Muscle weakness causing inability to perform usual social & functional activities	Disabling muscle weakness causing inability to perform basic self-care functions OR Respiratory muscle weakness impairing ventilation
Neurosensory	Asymptomatic with	Sensory alteration or	Sensory alteration or	Disabling sensory

CLINICAL				
**PARAMETER**	**GRADE 1 MILD**	**GRADE 2 MODERATE**	**GRADE 3 SEVERE**	**GRADE 4 POTENTIALLY LIFE-THREATENING**
alteration (including paresthesia and painful neuropathy)	sensory alteration on exam or minimal paresthesia causing no or minimal interference with usual social & functional activities	paresthesia causing greater than minimal interference with usual social & functional activities	paresthesia causing inability to perform usual social & functional activities	alteration or paresthesia causing inability to perform basic self-care functions
Seizure: (new onset) – **Adult ≥ 18 years**<br>See also Seizure: (known pre-existing seizure disorder)	NA	1 seizure	2 – 4 seizures	Seizures of any kind which are prolonged, repetitive (e.g., status epilepticus), or difficult to control (e.g., refractory epilepsy)
Seizure: (known pre-existing seizure disorder) **– Adult ≥ 18 years**<br>For worsening of existing epilepsy the grades should be based on an increase from previous level of control to any of these levels.	NA	Increased frequency of pre-existing seizures (non-repetitive) without change in seizure character OR Infrequent break-through seizures while on stable medication in a previously controlled seizure disorder	Change in seizure character from baseline either in duration or quality (e.g., severity or focality)	Seizures of any kind which are prolonged, repetitive (e.g., status epilepticus), or difficult to control (e.g., refractory epilepsy)
Seizure **– Pediatric < 18 years**	Seizure, generalized onset with or without secondary generalization, lasting < 5 minutes with < 24 hours post ictal state	Seizure, generalized onset with or without secondary generalization, lasting 5 – 20 minutes with < 24 hours post ictal state	Seizure, generalized onset with or without secondary generalization, lasting > 20 minutes	Seizure, generalized onset with or without secondary generalization, requiring intubation and sedation
Syncope (not associated with a procedure)	NA	Present	NA	NA
Vertigo	Vertigo causing no or minimal interference with usual social & functional activities	Vertigo causing greater than minimal interference with usual social & functional activities	Vertigo causing inability to perform usual social & functional activities	Disabling vertigo causing inability to perform basic self-care functions
**RESPIRATORY**				
Bronchospasm (acute)	FEV1 or peak flow reduced to 70 – 80%	FEV1 or peak flow 50 – 69%	FEV1 or peak flow 25 – 49%	Cyanosis OR FEV1 or peak flow < 25% OR Intubation
Dyspnea or respiratory distress				
**Adult ≥ 14 years**	Dyspnea on exertion with no or minimal interference with usual social & functional activities	Dyspnea on exertion causing greater than minimal interference with usual social & functional activities	Dyspnea at rest causing inability to perform usual social & functional activities	Respiratory failure with ventilatory support indicated
**Pediatric < 14 years**	Wheezing OR minimal increase in respiratory rate for age	Nasal flaring OR Intercostal retractions OR Pulse oximetry 90 – 95%	Dyspnea at rest causing inability to perform usual social & functional activities OR Pulse oximetry < 90%	Respiratory failure with ventilatory support indicated

CLINICAL				
**PARAMETER**	**GRADE 1 MILD**	**GRADE 2 MODERATE**	**GRADE 3 SEVERE**	**GRADE 4 POTENTIALLY LIFE-THREATENING**
**MUSCULOSKELETAL**				
Arthralgia<br>See also Arthritis	Joint pain causing no or minimal interference with usual social & functional activities	Joint pain causing greater than minimal interference with usual social & functional activities	Joint pain causing inability to perform usual social & functional activities	Disabling joint pain causing inability to perform basic self-care functions
Arthritis<br>See also Arthralgia	Stiffness or joint swelling causing no or minimal interference with usual social & functional activities	Stiffness or joint swelling causing greater than minimal interference with usual social & functional activities	Stiffness or joint swelling causing inability to perform usual social & functional activities	Disabling joint stiffness or swelling causing inability to perform basic self-care functions
Bone Mineral Loss				
**Adult ≥ 21 years**	BMD t-score -2.5 to -1.0	BMD t-score < -2.5	Pathological fracture (including loss of vertebral height)	Pathologic fracture causing life-threatening consequences
**Pediatric < 21 years**	BMD z-score -2.5 to -1.0	BMD z-score < -2.5	Pathological fracture (including loss of vertebral height)	Pathologic fracture causing life-threatening consequences
Myalgia<br>(non-injection site)	Muscle pain causing no or minimal interference with usual social & functional activities	Muscle pain causing greater than minimal interference with usual social & functional activities	Muscle pain causing inability to perform usual social & functional activities	Disabling muscle pain causing inability to perform basic self-care functions
Osteonecrosis	NA	Asymptomatic with radiographic findings AND No operative intervention indicated	Symptomatic bone pain with radiographic findings OR Operative intervention indicated	Disabling bone pain with radiographic findings causing inability to perform basic self-care functions
**GENITOURINARY**				
Cervicitis<br>(symptoms)<br>(For use in studies evaluating topical study agents)<br>For other cervicitis see Infection: Infection (any other than HIV infection)	Symptoms causing no or minimal interference with usual social & functional activities	Symptoms causing greater than minimal interference with usual social & functional activities	Symptoms causing inability to perform usual social & functional activities	Symptoms causing inability to perform basic self-care functions
Cervicitis<br>(clinical exam)<br>(For use in studies evaluating topical study agents)<br>For other cervicitis see Infection: Infection (any other than HIV infection)	Minimal cervical abnormalities on examination (erythema, mucopurulent discharge, or friability) OR Epithelial disruption<br>< 25% of total surface	Moderate cervical abnormalities on examination (erythema, mucopurulent discharge, or friability) OR Epithelial disruption of 25 – 49% total surface	Severe cervical abnormalities on examination (erythema, mucopurulent discharge, or friability) OR Epithelial disruption 50 – 75% total surface	Epithelial disruption<br>> 75% total surface
Inter-menstrual bleeding (IMB)	Spotting observed by participant OR Minimal blood observed during clinical or colposcopic	Inter-menstrual bleeding not greater in duration or amount than usual menstrual	Inter-menstrual bleeding greater in duration or amount than usual menstrual cycle	Hemorrhage with life-threatening hypotension OR Operative intervention indicated

CLINICAL				
**PARAMETER**	**GRADE 1 MILD**	**GRADE 2 MODERATE**	**GRADE 3 SEVERE**	**GRADE 4 POTENTIALLY LIFE-THREATENING**
	examination	cycle		
Urinary tract obstruction (e.g., stone)	NA	Signs or symptoms of urinary tract obstruction without hydronephrosis or renal dysfunction	Signs or symptoms of urinary tract obstruction with hydronephrosis or renal dysfunction	Obstruction causing life-threatening consequences
Vulvovaginitis (symptoms)<br>(Use in studies evaluating topical study agents)<br>For other vulvovaginitis see Infection: Infection (any other than HIV infection)	Symptoms causing no or minimal interference with usual social & functional activities	Symptoms causing greater than minimal interference with usual social & functional activities	Symptoms causing inability to perform usual social & functional activities	Symptoms causing inability to perform basic self-care functions
Vulvovaginitis (clinical exam)<br>(Use in studies evaluating topical study agents)<br>For other vulvovaginitis see Infection: Infection (any other than HIV infection)	Minimal vaginal abnormalities on examination OR Epithelial disruption < 25% of total surface	Moderate vaginal abnormalities on examination OR Epithelial disruption of 25 - 49% total surface	Severe vaginal abnormalities on examination OR Epithelial disruption 50 - 75% total surface	Vaginal perforation OR Epithelial disruption > 75% total surface
**OCULAR/VISUAL**				
Uveitis	Asymptomatic but detectable on exam	Symptomatic anterior uveitis OR Medical intervention indicated	Posterior or pan-uveitis OR Operative intervention indicated	Disabling visual loss in affected eye(s)
Visual changes (from baseline)	Visual changes causing no or minimal interference with usual social & functional activities	Visual changes causing greater than minimal interference with usual social & functional activities	Visual changes causing inability to perform usual social & functional activities	Disabling visual loss in affected eye(s)
**ENDOCRINE/METABOLIC**				
Abnormal fat accumulation (e.g., back of neck, breasts, abdomen)	Detectable by study participant (or by caregiver for young children and disabled adults)	Detectable on physical exam by health care provider	Disfiguring OR Obvious changes on casual visual inspection	NA
Diabetes mellitus	NA	New onset without need to initiate medication OR Modification of current medications to regain glucose control	New onset with initiation of medication indicated OR Diabetes uncontrolled despite treatment modification	Life-threatening consequences (e.g., ketoacidosis, hyperosmolar non-ketotic coma)
Gynecomastia	Detectable by study participant or caregiver (for young children and disabled adults)	Detectable on physical exam by health care provider	Disfiguring OR Obvious on casual visual inspection	NA
Hyperthyroidism	Asymptomatic	Symptomatic causing greater than minimal interference with usual	Symptoms causing inability to perform usual social & functional	Life-threatening consequences (e.g.,

CLINICAL				
**PARAMETER**	**GRADE 1 MILD**	**GRADE 2 MODERATE**	**GRADE 3 SEVERE**	**GRADE 4 POTENTIALLY LIFE-THREATENING**
		social & functional activities OR Thyroid suppression therapy indicated	activities OR Uncontrolled despite treatment modification	thyroid storm)
Hypothyroidism	Asymptomatic	Symptomatic causing greater than minimal interference with usual social & functional activities OR Thyroid replacement therapy indicated	Symptoms causing inability to perform usual social & functional activities OR Uncontrolled despite treatment modification	Life-threatening consequences (e.g., myxedema coma)
Lipoatrophy (e.g., fat loss from the face, extremities, buttocks)	Detectable by study participant (or by caregiver for young children and disabled adults)	Detectable on physical exam by health care provider	Disfiguring OR Obvious on casual visual inspection	NA

LABORATORY				
**PARAMETER**	**GRADE 1**<br>**MILD**	**GRADE 2**<br>**MODERATE**	**GRADE 3**<br>**SEVERE**	**GRADE 4**<br>**POTENTIALLY LIFE-THREATENING**
**HEMATOLOGY*Standard International Units are listed in italics***				
Absolute CD4+ count **–Adult and Pediatric > 13 years** (HIV NEGATIVE ONLY)	300 – 400/mm^3<br>*300 – 400/µL*	200 – 299/mm^3<br>*200 – 299/µL*	100 – 199/mm^3<br>*100 – 199/µL*	< 100/mm^3<br>*< 100/µL*
Absolute lymphocyte count **–Adult and Pediatric > 13 years** (HIV NEGATIVE ONLY)	600 – 650/mm^3<br>*0.600 x 10^9 – 0.650 x 10^9/L*	500 – 599/mm^3<br>*0.500 x 10^9 – 0.599 x 10^9/L*	350 – 499/mm^3<br>*0.350 x 10^9 – 0.499 x 10^9/L*	< 350/mm^3<br>*<0.350 x 10^9/L*
Absolute neutrophil count (ANC)				
**Adult and Pediatric, > 7 days**	1,000 – 1,300/mm^3<br>*1.000 x 10^9 – 1.300 x 10^9/L*	750 – 999/mm^3<br>*0.750 x 10^9 – 0.999 x 10^9/L*	500 – 749/mm^3<br>*0.500 x 10^9 – 0.749 x 10^9/L*	< 500/mm^3<br>*<0.500 x 10^9/L*
**Infant*†, 2 – ≤ 7 days**	1,250 – 1,500/mm^3<br>*1.250 x 10^9 – 1.500 x 10^9/L*	1,000 – 1,249/mm^3<br>*1.000 x 10^9 – 1.249 x 10^9/L*	750 – 999/mm^3<br>*0.750 x 10^9 – 0.999 x 10^9/L*	< 750/mm^3<br>*< 0.750 x 10^9/L*
**Infant*†, 1 day**	4,000 – 5,000/mm^3<br>*4.000 x 10^9 – 5.000 x 10^9/L*	3,000 – 3,999/mm^3<br>*3.000 x 10^9 – 3.999 x10^9/L*	1,500 – 2,999/mm^3<br>*1.500 x 10^9 – 2.999 x 10^9/L*	< 1,500/mm^3<br>*< 1.500 x 10^9/L*
Fibrinogen, decreased	100 – 200 mg/dL<br>*1.00 – 2.00 g/L*<br>OR<br>0.75 – 0.99 x LLN	75 – 99 mg/dL<br>*0.75 – 0.99 g/L*<br>OR<br>0.50 – 0.74 x LLN	50 – 74 mg/dL<br>*0.50 – 0.74 g/L*<br>OR<br>0.25 – 0.49 x LLN	< 50 mg/dL<br>*< 0.50 g/L*<br>OR<br>< 0.25 x LLN<br>OR<br>Associated with gross bleeding
Hemoglobin (Hgb)				
**Adult and Pediatric ≥ 57 days** (HIV POSITIVE ONLY)	8.5 – 10.0 g/dL<br>*1.32 – 1.55 mmol/L*	7.5 – 8.4 g/dL<br>*1.16 – 1.31 mmol/L*	6.50 – 7.4 g/dL<br>*1.01 – 1.15 mmol/L*	< 6.5 g/dL<br>*< 1.01 mmol/L*
**Adult and Pediatric ≥ 57 days** (HIV NEGATIVE ONLY)	10.0 – 10.9 g/dL<br>*1.55 – 1.69 mmol/L*<br>OR<br>Any decrease 2.5 – 3.4 g/dL<br>*0.39 – 0.53 mmol/L*	9.0 – 9.9 g/dL<br>*1.40 – 1.54 mmol/L*<br>OR<br>Any decrease 3.5 – 4.4 g/dL<br>*0.54 – 0.68 mmol/L*	7.0 – 8.9 g/dL<br>*1.09 – 1.39 mmol/L*<br>OR<br>Any decrease ≥ 4.5 g/dL<br>*≥ 0.69 mmol/L*	< 7.0 g/dL<br>*< 1.09 mmol/L*
**Infant*†, 36 – 56 days** (HIV POSITIVE OR NEGATIVE)	8.5 – 9.4 g/dL<br>*1.32 – 1.46 mmol/L*	7.0 – 8.4 g/dL<br>*1.09 – 1.31 mmol/L*	6.0 – 6.9 g/dL<br>*0.93 – 1.08 mmol/L*	< 6.00 g/dL<br>*< 0.93 mmol/L*
**Infant*†, 22 – 35 days** (HIV POSITIVE OR NEGATIVE)	9.5 – 10.5 g/dL<br>*1.47 – 1.63 mmol/L*	8.0 – 9.4 g/dL<br>*1.24 – 1.46 mmol/L*	7.0 – 7.9 g/dL<br>*1.09 – 1.23 mmol/L*	< 7.00 g/dL<br>*<1.09 mmol/L*
**Infant*†, 1 – 21 days** (HIV POSITIVE OR NEGATIVE)	12.0 – 13.0 g/dL<br>*1.86 – 2.02 mmol/L*	10.0 – 11.9 g/dL<br>*1.55 – 1.85 mmol/L*	9.0 – 9.9 g/dL<br>*1.40 – 1.54 mmol/L*	< 9.0 g/dL<br>*< 1.40 mmol/L*
International Normalized Ratio of prothrombin time (INR)	1.1 – 1.5 x ULN	1.6 – 2.0 x ULN	2.1 – 3.0 x ULN	> 3.0 x ULN
Methemoglobin	5.0 – 10.0%	10.1 – 15.0%	15.1 – 20.0%	> 20.0%

LABORATORY				
**PARAMETER**	**GRADE 1 MILD**	**GRADE 2 MODERATE**	**GRADE 3 SEVERE**	**GRADE 4 POTENTIALLY LIFE-THREATENING**
Prothrombin Time (PT)	1.1 – 1.25 x ULN	1.26 – 1.50 x ULN	1.51 – 3.00 x ULN	> 3.00 x ULN
Partial Thromboplastin Time (PTT)	1.1 – 1.66 x ULN	1.67 – 2.33 x ULN	2.34 – 3.00 x ULN	> 3.00 x ULN
Platelets, decreased	100,000 – 124,999/mm^3 *100.000 x 10^9 – 124.999 x 10^9/L*	50,000 – 99,999/mm^3 *50.000 x 10^9 – 99.999 x 10^9/L*	25,000 – 49,999/mm^3 *25.000 x 10^9 – 49.999 x 10^9/L*	< 25,000/mm^3 *< 25.000 x 10^9/L*
WBC, decreased	2,000 – 2,500/mm^3 *2.000 x 10^9 – 2.500 x 10^9/L*	1,500 – 1,999/mm^3 *1.500 x 10^9 – 1.999 x 10^9/L*	1,000 – 1,499/mm^3 *1.000 x 10^9 – 1.499 x 10^9/L*	< 1,000/mm^3 *< 1.000 x 10^9/L*
**CHEMISTRIES** ***Standard International Units are listed in italics***				
Acidosis	NA	pH < normal, but ≥ 7.3	pH < 7.3 without life-threatening consequences	pH < 7.3 with life-threatening consequences
Albumin, serum, low	3.0 g/dL – < LLN *30 g/L –< LLN*	2.0 – 2.9 g/dL *20 – 29 g/L*	< 2.0 g/dL *< 20 g/L*	NA
Alkaline Phosphatase	1.25 – 2.5 x ULN†	2.6 – 5.0 x ULN†	5.1 – 10.0 x ULN†	> 10.0 x ULN†
Alkalosis	NA	pH > normal, but ≤ 7.5	pH > 7.5 without life-threatening consequences	pH > 7.5 with life-threatening consequences
ALT (SGPT)	1.25 – 2.5 x ULN	2.6 – 5.0 x ULN	5.1 – 10.0 x ULN	> 10.0 x ULN
AST (SGOT)	1.25 – 2.5 x ULN	2.6 – 5.0 x ULN	5.1 – 10.0 x ULN	> 10.0 x ULN
Bicarbonate, serum, low	16.0 mEq/L – < LLN *16.0 mmol/L –< LLN*	11.0 – 15.9 mEq/L *11.0 – 15.9 mmol/L*	8.0 – 10.9 mEq/L *8.0 – 10.9 mmol/L*	< 8.0 mEq/L *< 8.0 mmol/L*
Bilirubin (Total)				
**Adult and Pediatric > 14 days**	1.1 – 1.5 x ULN	1.6 – 2.5 x ULN	2.6 – 5.0 x ULN	> 5.0 x ULN
**Infant*†, ≤ 14 days** (non-hemolytic)	NA	20.0 – 25.0 mg/dL *342 – 428 µmol/L*	25.1 – 30.0 mg/dL *429 – 513 µmol/L*	> 30.0 mg/dL *>513.0 µmol/L*
**Infant*†, ≤ 14 days** (hemolytic)	NA	NA	20.0 – 25.0 mg/dL *342 – 428 µmol/L*	> 25.0 mg/dL *>428 µmol/L*
Calcium, serum, high (corrected for albumin)				
**Adult and Pediatric ≥ 7 days**	10.6 – 11.5 mg/dL *2.65 – 2.88 mmol/L*	11.6 – 12.5 mg/dL *2.89 – 3.13 mmol/L*	12.6 – 13.5 mg/dL *3.14 – 3.38 mmol/L*	> 13.5 mg/dL *> 3.38 mmol/L*
**Infant*†,< 7 days**	11.5 – 12.4 mg/dL *2.88 – 3.10 mmol/L*	12.5 – 12.9 mg/dL *3.11 – 3.23 mmol/L*	13.0 – 13.5 mg/dL *3.245 – 3.38 mmol/L*	> 13.5 mg/dL *> 3.38 mmol/L*
Calcium, serum, low (corrected for albumin)				
**Adult and Pediatric ≥ 7 days**	7.8 – 8.4 mg/dL *1.95 – 2.10 mmol/L*	7.0 – 7.7 mg/dL *1.75 – 1.94 mmol/L*	6.1 – 6.9 mg/dL *1.53 – 1.74 mmol/L*	< 6.1 mg/dL *< 1.53 mmol/L*
**Infant*†,< 7 days**	6.5 – 7.5 mg/dL *1.63 – 1.88 mmol/L*	6.0 – 6.4 mg/dL *1.50 – 1.62 mmol/L*	5.50 – 5.90 mg/dL *1.38 – 1.51 mmol/L*	< 5.50 mg/dL *< 1.38 mmol/L*
Cardiac troponin I (cTnI)	NA	NA	NA	Levels consistent with myocardial infarction or unstable angina as defined by the manufacturer

LABORATORY				
**PARAMETER**	**GRADE 1 MILD**	**GRADE 2 MODERATE**	**GRADE 3 SEVERE**	**GRADE 4 POTENTIALLY LIFE-THREATENING**
Cardiac troponin T (cTnT)	NA	NA	NA	≥ 0.20 ng/mL OR Levels consistent with myocardial infarction or unstable angina as defined by the manufacturer
Cholesterol (fasting)				
**Adult ≥ 18 years**	200 – 239 mg/dL *5.18 – 6.19 mmol/L*	240 – 300 mg/dL *6.20 – 7.77 mmol/L*	> 300 mg/dL *>7.77 mmol/L*	NA
**Pediatric < 18 years**	170 – 199 mg/dL *4.40 – 5.15 mmol/L*	200 – 300 mg/dL *5.16 – 7.77 mmol/L*	> 300 mg/dL *> 7.77 mmol/L*	NA
Creatine Kinase	3.0 – 5.9 x ULN†	6.0 – 9.9 x ULN†	10.0 – 19.9 x ULN†	≥20.0 x ULN†
Creatinine	1.1 – 1.3 x ULN†	1.4 – 1.8 x ULN†	1.9 – 3.4 x ULN†	≥ 3.5 x ULN†
Glucose, serum, high				
Nonfasting	116 – 160 mg/dL *6.44 – 8.88 mmol/L*	161 – 250 mg/dL *8.89 – 13.88 mmol/L*	251 – 500 mg/dL *13.89 – 27.75 mmol/L*	> 500 mg/dL *> 27.75 mmol/L*
Fasting	110 – 125 mg/dL *6.11 – 6.94 mmol/L*	126 – 250 mg/dL *6.95 – 13.88 mmol/L*	251 – 500 mg/dL *13.89 – 27.75 mmol/L*	> 500 mg/dL *> 27.75 mmol/L*
Glucose, serum, low				
**Adult and Pediatric ≥ 1 month**	55 – 64 mg/dL *3.05 – 3.55 mmol/L*	40 – 54 mg/dL *2.22 – 3.06 mmol/L*	30 – 39 mg/dL *1.67 – 2.23 mmol/L*	< 30 mg/dL *< 1.67 mmol/L*
**Infant*†,< 1 month**	50 – 54 mg/dL *2.78 – 3.00 mmol/L*	40 – 49 mg/dL *2.22 – 2.77 mmol/L*	30 – 39 mg/dL *1.67 – 2.21 mmol/L*	< 30 mg/dL *< 1.67 mmol/L*
Lactate	< 2.0 x ULN without acidosis	≥ 2.0 x ULN without acidosis	Increased lactate with pH < 7.3 without life-threatening consequences	Increased lactate with pH < 7.3 with life-threatening consequences
LDL cholesterol (fasting)				
**Adult ≥ 18 years**	130 – 159 mg/dL *3.37 – 4.12 mmol/L*	160 – 190 mg/dL *4.13 – 4.90 mmol/L*	≥ 190 mg/dL *≥ 4.91 mmol/L*	NA
**Pediatric > 2 - < 18 years**	110 – 129 mg/dL *2.85 – 3.34 mmol/L*	130 – 189 mg/dL *3.35 – 4.90 mmol/L*	≥ 190 mg/dL *≥ 4.91 mmol/L*	NA
Lipase	1.1 – 1.5 x ULN	1.6 – 3.0 x ULN	3.1 – 5.0 x ULN	> 5.0 x ULN
Magnesium, serum, low	1.2 – 1.4 mEq/L *0.60 – 0.70 mmol/L*	0.9 – 1.1 mEq/L *0.45 – 0.59 mmol/L*	0.6 – 0.8 mEq/L *0.30 – 0.44 mmol/L*	< 0.60 mEq/L *< 0.30 mmol/L*
Pancreatic amylase	1.1 – 1.5 x ULN	1.6 – 2.0 x ULN	2.1 – 5.0 x ULN	> 5.0 x ULN
Phosphate, serum, low				
**Adult and Pediatric > 14 years**	2.5 mg/dL – < LLN *0.81 mmol/L –< LLN*	2.0 – 2.4 mg/dL *0.65 – 0.80 mmol/L*	1.0 – 1.9 mg/dL *0.32 – 0.64 mmol/L*	< 1.00 mg/dL *< 0.32 mmol/L*
**Pediatric 1 year – 14 years**	3.0 – 3.5 mg/dL *0.97 – 1.13 mmol/L*	2.5 – 2.9 mg/dL *0.81 – 0.96 mmol/L*	1.5 – 2.4 mg/dL *0.48 – 0.80 mmol/L*	< 1.50 mg/dL *< 0.48 mmol/L*
**Pediatric < 1 year**	3.5 – 4.5 mg/dL *1.13 – 1.45 mmol/L*	2.5 – 3.4 mg/dL *0.81 – 1.12 mmol/L*	1.5 – 2.4 mg/dL *0.48 – 0.80 mmol/L*	< 1.50 mg/dL *< 0.48 mmol/L*
Potassium, serum, high	5.6 – 6.0 mEq/L *5.6 – 6.0 mmol/L*	6.1 – 6.5 mEq/L *6.1 – 6.5 mmol/L*	6.6 – 7.0 mEq/L *6.6 – 7.0 mmol/L*	> 7.0 mEq/L *> 7.0 mmol/L*

LABORATORY				
**PARAMETER**	**GRADE 1**<br>**MILD**	**GRADE 2**<br>**MODERATE**	**GRADE 3**<br>**SEVERE**	**GRADE 4**<br>**POTENTIALLY LIFE-THREATENING**
Potassium, serum, low	3.0 – 3.4 mEq/L<br>*3.0 – 3.4 mmol/L*	2.5 – 2.9 mEq/L<br>*2.5 – 2.9 mmol/L*	2.0 – 2.4 mEq/L<br>*2.0 – 2.4 mmol/L*	< 2.0 mEq/L<br>*< 2.0 mmol/L*
Sodium, serum, high	146 – 150 mEq/L<br>*146 – 150 mmol/L*	151 – 154 mEq/L<br>*151 – 154 mmol/L*	155 – 159 mEq/L<br>*155 – 159 mmol/L*	≥ 160 mEq/L<br>*≥ 160 mmol/L*
Sodium, serum, low	130 – 135 mEq/L<br>*130 – 135 mmol/L*	125 – 129 mEq/L<br>*125 – 129 mmol/L*	121 – 124 mEq/L<br>*121 – 124 mmol/L*	≤ 120 mEq/L<br>*≤ 120 mmol/L*
Triglycerides (fasting)	NA	500 – 750 mg/dL<br>*5.65 – 8.48 mmol/L*	751 – 1,200 mg/dL<br>*8.49 – 13.56 mmol/L*	> 1,200 mg/dL<br>*> 13.56 mmol/L*
Uric acid	7.5 – 10.0 mg/dL<br>*0.45 – 0.59 mmol/L*	10.1 – 12.0 mg/dL<br>*0.60 – 0.71 mmol/L*	12.1 – 15.0 mg/dL<br>*0.72 – 0.89 mmol/L*	> 15.0 mg/dL<br>*> 0.89 mmol/L*
**URINALYSIS***Standard International Units are listed in italics*				
Hematuria (microscopic)	6 – 10 RBC/HPF	> 10 RBC/HPF	Gross, with or without clots OR with RBC casts	Transfusion indicated
Proteinuria, random collection	1 +	2 – 3 +	4 +	NA
Proteinuria, 24 hour collection				
**Adult and Pediatric ≥ 10 years**	200 – 999 mg/24 h<br>*0.200 – 0.999 g/d*	1,000 – 1,999 mg/24 h<br>*1.000 – 1.999 g/d*	2,000 – 3,500 mg/24 h<br>*2.000 – 3.500 g/d*	> 3,500 mg/24 h<br>*> 3.500 g/d*
**Pediatric > 3 mo - < 10 years**	201 – 499 mg/m^2/24 h<br>*0.201 – 0.499 g/d*	500 – 799 mg/m^2/24 h<br>*0.500 – 0.799 g/d*	800 – 1,000 mg/m^2/24 h<br>*0.800 – 1.000 g/d*	> 1,000 mg/ m^2/24 h<br>*> 1.000 g/d*

APPENDIX 9: ADDITIONAL BACKGROUND INFORMATION

(I) The immune response to HIV

(i) The role of T-cells

Whilst CD4 T-cells are key regulators of humoral and cellular adaptive immune responses, CD8 T-cells have often been the main focus of T-cell vaccine development because they are able to kill virally infected cells. HIV specific T-cell responses contribute directly towards protective immunity in those that are naturally infected [1][2] and the development of such cellular responses coincides with the control of acute HIV-1 viremia in both humans and macaques. This has been taken as evidence that T-cells are instrumental in controlling viral replication, especially as there is no such correlation between the kinetics of viral control and appearance of Ab responses. Recent work in animal models has suggested that recombinant viral vectors which stimulate a broad and strong CD8+ T-cell response can also partially protect against challenge with the highly virulent SIV mac239 strain [3][4] and a recently described macaque model using a cytomegalovirus vector able to continuously deliver SIV antigens suggests that the persistence of immune response might be central to efficacy. One area which is particularly poorly understood is why so many SIV and HIV-specific T-cells are so poor at killing virally infected (as opposed to peptide pulsed) target cells in vitro [5] and a deeper understanding of the relationship between the phenotype of a T-cell and its protective potential is urgently needed. There is also a need for studies employing "functional" readouts which better reflect what is needed to control a natural infection. Ideally these assays should also be validated for incorporation as endpoints in clinical trials [6].

(ii) The role of antibodies

Passive transfer experiments in macaques have already shown that broadly physiological levels of an existing broadly neutralising antibody are able to block infection following low dose intra-vaginal challenge with SIV [7, 8] and whilst immunogens are designed, plans are underway to conduct passive transfer studies with human monoclonal neutralising antibodies to see whether this would also be feasible as a prevention strategy. Non-neutralising antibodies have come back into the spotlight again in light of the results of RV144 and there are passive transfer studies underway in NHP using monoclonal antibodies derived from those which were shown to correlate with reduced risk of infection.

It is still far from clear how to induce neutralising antibody responses with a vaccine. A significant minority of HIV infections result in heterogeneous mixtures of polyclonal broadly neutralizing antibodies with broad specificity for the virus envelope [9]. After a concerted effort several broadly neutralising antibodies have been isolated from HIV infected individuals and cloned. Whilst these can inform rational design, the affinity maturation of these broadly neutralising antibodies is not well understood and it seems more complex than for other humoral responses. The antibodies elicited by vaccines to date have not been protective and many of the most relevant epitopes on the viral envelope remain undefined. Some of the underlying reasons for this are now being understood. The target of the most potent neutralising antibodies, the envelope spike, is very unstable and difficult to synthesise in the relevant immunogenic form [10] and the most conserved epitopes within this spike are some of the most difficult to access [11]. Even if it were possible to induce neutralising antibodies, access to infectious virions might also prove limiting. Importantly, it is also not yet known whether virus is transmitted in a predominantly cell-free or intracellular form *in vivo* and it is also still far from clear precisely how the virus passes between cells. This is particularly relevant because there is some doubt about any role for neutralising antibodies in processes such as synapse mediated transmission [12]. It is anticipated that the insights gained from

the structural analysis of naturally occurring antibodies will feed directly into rational vaccine design [13].

(iii) RV144 and immune correlates protection

Elispot T-cell responses were only seen in ~17% of vaccinees and there were no T-cell responses which correlated with protection from infection, although >90% of those vaccinated made non- neutralising (and relatively short-lived) antibody responses to the vaccine. The exhaustive immunological analysis of available samples from a small subset of the cohort has very recently been completed and initial results (unpublished) suggest that circulating levels of IgG non-neutralising antibodies with specificity for the V1/V2 loop of the viral envelope are correlated with a reduced risk of subsequent infection whereas levels of IgA of the same specificity are positively correlated with risk of infection [14]. The latest results have had a significant impact on the field, shifting strategic focus back to antibodies and impacting on the design of ongoing trials. Initially, the strategic focus of the field was protective antibodies and when there was no demonstrable efficacy using this approach in clinical trials, the emphasis switched to the development of T-cell immunogens. The first efficacy trials (VAXgen) started in North America and Europe in 1998 and in Thailand in 1999, and assessed the efficacy of a recombinant gp120 HIV-1 envelope protein (AIDSVAX B/E). Preliminary results from the North American Phase III randomised trial which recruited 5095 individuals, showed that whilst the protein used was safe and immunogenic, it failed to confer protection [15]. Results from the trial in Thailand endorsed the findings in a different setting [16]. Two large phase IIB trials of a "T-cell vaccine" (STEP and Phambili) using a non-replicating recombinant Adenovirus 5 vector encoding subtype B gag, pol and nef were terminated due to futility and worryingly, because there was also a suggestion of increased risk of HIV acquisition in some of those who received the vaccine- although whether this increased susceptibility was caused by vaccine itself is still debated [17, 18].

In late 2011, after 2 years of unprecedented intensive and collaborative immunological analysis of samples from the vaccinees, immunological correlates were presented at the AIDS vaccine conference in Bangkok. The levels of two types of serum antibody correlated with the risk of infection: Levels of IgG against the V1/V2 loop of the viral envelope were negatively correlated with risk of infection and levels of IgA with the same specificity were positively correlated. After preliminary screening of archived samples in order to identify potentially informative immune responses for the analysis, those which correlated with "protection" were identified using a set of "case-control" samples selected from the original cohort. This case control set of samples was derived from 41 vaccinees who became infected, 205 who were vaccinated and remained uninfected and 40 who received placebo. Six primary and ~30 secondary assays made it through to the final correlates analysis which was pre-defined. Assays were selected on the basis of fulfilling certain criteria in terms of reproducibility and performance. After two separate multivariate analyses, which included all six of the main immune responses of interest and were controlled for age, gender and baseline behavioural risk; two immune responses correlated with the risk of acquiring HIV. The levels of circulating IgG antibodies to V1/V2 variable loops in the HIV envelope correlated with levels of protection, whereas circulating levels of (monomeric) IgA were correlated with risk of infection. Titres of monomeric (serum) IgA antibodies did not correlate with an increased susceptibility to infection, but rather seemed to interfere with vaccine efficacy because the risk was not increased relative to placebo. One suggestion which was made by Bart Haynes (chair of the "correlates analysis group" Steering committee)- was that monomeric IgA in serum might interfere with antibody-dependent cellular cytotoxicity (ADCC) and so impact on the destruction of virally infected cells- as has already been described in cancer (AIDS Vaccine 2011 Plenary session). Bart Haynes also emphasised the fact that what had been described were correlates of risk and thus causative link(s) could not be inferred. The analysis was 80% powered to detect an approximately 50% reduction in HIV infection rate and so it was also acknowledged that weaker correlates might have been missed. In spite of these exciting data, the protective effect of the vaccine was modest and relatively shortlived. T-cell immune

responses were relatively weak and the results have not shifted the consensus view that the optimal vaccine against HIV would need to generate strong cellular and humoral immune responses- both of which have been shown to contribute to control of natural infection.

(iv) Current vaccination strategies

Host responses to natural and experimental infections suggest that that there are protective immune responses which could be harnessed by a vaccine [19, 20]. Interestingly, although associated with a ~31% reduction in infection, vaccination in RV144 was not associated with a reduction in viral load set point or any other sustained clinical parameters related to improved outcomes [20].

Immune correlates of protection are poorly understood but it is now widely agreed that broad and durable humoral and cellular immune responses are both needed - ideally present at the portals of virus entry at the time of first exposure. Nonhuman primate models have been extensively used to assess vaccine efficacy in experimental challenge studies and the limitations and potential relevance of such models to vaccine efficacy have been reviewed elsewhere [21]. More than 30 candidate HIV vaccines have been taken forward into early phase clinical trials on the basis of the demonstration of efficacy in nonhuman primates but to date this has only translated in to one (modestly) successful trial in humans.

(v) Heterologous prime boost vaccination regimes.

In a trial which enrolled participants who had previously been primed with either DNA or Ad5 alone, the advantages of the heterologous prime boost strategy were evident, with an approximately 7 fold increase in immune responses following Ad5 boosting after DNA priming. This effect was evident in both the T-cell and B-cell compartments [22]**.** In the HIVIS 03 trial which recruited healthy volunteers in Tanzania, 58% of individuals receiving three injections with multiclade DNA made a positive γ-IFN T-cell Elispot response and this increased to ~100% after boosting twice with MVA. Interestingly, due to problems with product availability, up to 3 years elapsed between the two MVA-CMDR immunisations in this trial. There have been concerns about the dampening effects of vector-specific responses on vaccine immunogenicity and the striking T-cell and B-cell responses seen in this trial may have been due to this (impractically) drawn out schedule.

Apart from intrinsic flexibility and relatively modest manufacturing costs, the rationale for using DNA as a component of HIV vaccines stems also from a desire to induce cytotoxic T cells-responses which depend largely upon the expression of endogenous proteins. Unfortunately, the potency of DNA vaccines first described in animal models has not been realised in the clinic, and this had led to a focus on the development of methods specifically focussed on increasing the efficiency of DNA uptake by cells. Electroporation and the use of needle free devices such as the Biojector have both been shown to increase the immunogenicity of DNA although, to date, electroporation has only been shown to be effective in one clinical trial and the choice of device used is probably key to success [23]. We believe that the cumulative dose of DNA used in priming is crucial in determining the breadth and magnitude of the T-cell response (in this case 12mg given over 8 weeks). The uptake of DNA after IM administration is known to be highly inefficient (~5%) and this may be one of the explanations of the relatively poor performance of DNA vaccines in the clinic. We propose that Electroporation and the Biojector device are included in the proposed trial with the explicit aim of improving the efficiency of uptake of DNA by target cells.

(II) Work leading to this trial

(i) The vaccine

There have been several trials leading to the vaccination strategy centered around priming with 7 plasmids encoding p37 gag (clade A and B), RT (clade B), gp160 env (clade A, B and C) and rev (clade B) followed by boosting with MVA-CMDR encoding gp150 ENV (clade E), p55 gag (clade A) and pro/rt (clade A). HIVIS 01/02 was conducted in Sweden in healthy volunteers, and demonstrated the clear immunological benefit of boosting with MVA after priming with DNA. 30% of vaccinees responded to 3 doses of 1.0 mg DNA given at 0, 4 and 12 week intradermally (ID), and this increased to 92% after a single boost with MVA-CMDR given intramuscularly (IM) 24 weeks later [24]. Interestingly, there was a clear advantage of a second, albeit very delayed boost with MVA-CMDR upon antibody responses to ENV. This additional boost was given to some of those who had participated in HIVIS01/02 and who had completed the vaccination regimen (HIVIS05). After 3 DNA priming injections and one boost with MVA-CMDR, 57% of individuals generated p37GAG-specific systemic antibodies but only 5% HIV-1 GP160 ENV-specific antibodies. Four weeks after a second boost with MVA-CMDR, given 3 years later, 96% of those vaccinated had developed ENV and 100% GAG-specific antibodies suggesting the advantage of a long gap between boosts with the modified virus (G. Biberfeld, personal communication). HIVIS 03 built on the these results in an African setting and demonstrated the dose sparing impact of delivering the DNA ID relative to IM and showed, if anything, increased immunogenicity using the same DNA and MVA in healthy volunteers in Tanzania. Approximately 58% of individuals made T-cell responses in response to 3 injections of 1.0mg DNA given ID using the needle free Biojector, and this increased to 100% after one boost with MVA-CMDR given IM 6 months later. All vaccinees had HIV-specific lymphoproliferative responses and their sera reacted in diagnostic serological tests. 90% had gp160- specific antibodies after a second MVA boost at ~ 84 weeks [25].

The (ongoing) TaMoVac I (TM01) trial is also focussed on the optimisation of DNA priming by exploring (i) the impact of reducing the dose and concentration of DNA and (ii) the effects of pooling the plasmid pools encoding env and gag and injecting them into one rather than two arms. Priming with separate plasmid pools has been shown to favour the development of balanced ENV and GAG-specific immune responses in the mouse. The results of TM01 will directly inform the design of TM02 and interim analysis of the immunogenicity data will be carried out early in 2012 to allow a rational choice of the DNA to be used. The trial has been carried out in two sites in Tanzania and volunteers are randomised to receive 600µg DNA plasmids pools encoding (env/rev) or (gag/pol) peptides, injected either in separate pools or combined together. A third group will serve as a reference and will receive 1.0mg DNA delivered ID in separate pools in accordance with HIVIS 01/2/3. Based on previous results, balanced T-cell responses and systemic binding antibodies responses are expected in ~ 100% of individuals in TM01 (at least in the group receiving high dose DNA) after a second MVA boost. There are ongoing plans to amend the protocol, and by re-boosting the participants twice with rGP140/GLA-AF four weeks apart, the expectation is that this will further favour the development of ENV-specific antibody responses. We anticipate that the results of TM01 will complement those of the proposed trial by shedding light on the impact of boosting with a Clade C envelope protein after priming potent B and T-cell responses with multiclade DNA and MVA.

(ii) The use of Electroporation to improve vaccination efficiency

In vivo electroporation has been proven to be an effective method of gene delivery into muscle [26][27][28, 29]. In various animal studies, in vivo electroporation has also been shown to markedly enhance humoral and cellular immune responses in mice, [30-32][33][34], rabbits [35][36][37], pigs, cattle [38, 39]and non-human primates [40][41][42][43][44].
Another Phase I/IIa employing EP is being conducted by Tripep AB in collaboration with the Karolinska Institute in Sweden to test a therapeutic Hepatitis C DNA vaccine (CHRONVAC-C)

in chronic hepatitis C patients (www.clinicaltrials.gov, ID: NCT00563173). This study is exploring the safety and efficacy of a device produced by Inovio Biomedical Corporation. Preliminary results from Inovio and Tripep AB show the vaccine delivered by EP to be safe, tolerable and to generate detectable cell-mediated immune responses in five individuals given four injections of the low dose group. A recent phase I HIV vaccine trial (RV262) sponsored by DAIDS/NIAID evaluating the safety and immunogenicity of the PENNVAX™-G DNA administered by IM Biojector® 2000 or IM electroporation using the CELLECTRA® device followed by MVA-CMDR boost in healthy HIV uninfected adults was initiated. The trial started in October 2010 in the U.S. and is planned to be initiated in Tanzania (Mbeya), Kenya, Uganda and Thailand after interim safety and tolerability data from the U.S. have been analysed showing confirming results to continue with the study.

References

1. Rowland-Jones, S., et al., *HIV-specific cytotoxic T-cells in HIV-exposed but uninfected Gambian women.* Nat Med, 1995. **1**(1): p. 59-64.
2. Mazzoli, S., et al., *HIV-specific mucosal and cellular immunity in HIV-seronegative partners of HIV-seropositive individuals.* Nat Med, 1997. **3**(11): p. 1250-7.
3. Wilson, N.A., et al., *Vaccine-induced cellular responses control simian immunodeficiency virus replication after heterologous challenge.* J Virol, 2009. **83**(13): p. 6508-21.
4. Liu, J., et al., *Immune control of an SIV challenge by a T-cell-based vaccine in rhesus monkeys.* Nature, 2009. **457**(7225): p. 87-91.
5. Chung, C., et al., *Not all cytokine-producing CD8+ T cells suppress simian immunodeficiency virus replication.* J Virol, 2007. **81**(3): p. 1517-23.
6. Spentzou, A., et al., *Viral inhibition assay: a CD8 T cell neutralization assay for use in clinical trials of HIV-1 vaccine candidates.* J Infect Dis, 2010. **201**(5): p. 720-9.
7. Hessell, A.J., et al., *Broadly neutralizing human anti-HIV antibody 2G12 is effective in protection against mucosal SHIV challenge even at low serum neutralizing titers.* PLoS Pathog, 2009. **5**(5): p. e1000433.
8. Hessell, A.J., et al., *Effective, low-titer antibody protection against low-dose repeated mucosal SHIV challenge in macaques.* Nat Med, 2009. **15**(8): p. 951-4.
9. Scheid, J.F., et al., *Broad diversity of neutralizing antibodies isolated from memory B cells in HIV-infected individuals.* Nature, 2009. **458**(7238): p. 636-40.
10. Pancera, M., et al., *Structure of HIV-1 gp120 with gp41-interactive region reveals layered envelope architecture and basis of conformational mobility.* Proc Natl Acad Sci U S A, 2010. **107**(3): p. 1166-71.
11. Chen, L., et al., *Structural basis of immune evasion at the site of CD4 attachment on HIV-1 gp120.* Science, 2009. **326**(5956): p. 1123-7.
12. Hubner, W., et al., *Quantitative 3D video microscopy of HIV transfer across T cell virological synapses.* Science, 2009. **323**(5922): p. 1743-7.
13. Walker, L.M., et al., *Broad and potent neutralizing antibodies from an African donor reveal a new HIV-1 vaccine target.* Science, 2009. **326**(5950): p. 285-9.
14. Haynes, B.F., *Case-control study of the RV144 for immune correlates:the analysis and way forward.* AIDS vaccine 2011, 12015 September 2011, Bangkok, Thailand [Abstract PL01.04], 2011.
15. Flynn, N.M., et al., *Placebo-controlled phase 3 trial of a recombinant glycoprotein 120 vaccine to prevent HIV-1 infection.* J Infect Dis, 2005. **191**(5): p. 654-65.
16. Pitisuttithum, P., et al., *Randomized, double-blind, placebo-controlled efficacy trial of a bivalent recombinant glycoprotein 120 HIV-1 vaccine among injection drug users in Bangkok, Thailand.* J Infect Dis, 2006. **194**(12): p. 1661-71.
17. Buchbinder, S.P., et al., *Efficacy assessment of a cell-mediated immunity HIV-1 vaccine (the Step Study): a double-blind, randomised, placebo-controlled, test-of-concept trial.* Lancet, 2008. **372**(9653): p. 1881-93.
18. D'Souza, M.P. and N. Frahm, *Adenovirus 5 serotype vector-specific immunity and HIV-1 infection: a tale of T cells and antibodies.* Aids, 2010. **24**(6): p. 803-9.

19. Johnston, M.I. and A.S. Fauci, *An HIV vaccine--challenges and prospects.* N Engl J Med, 2008. **359**(9): p. 888-90.
20. McElrath, M.J. and B.F. Haynes, *Induction of immunity to human immunodeficiency virus type-1 by vaccination.* Immunity, 2011. **33**(4): p. 542-54.
21. Sodora, D.L., et al., *Toward an AIDS vaccine: lessons from natural simian immunodeficiency virus infections of African nonhuman primate hosts.* Nat Med, 2009. **15**(8): p. 861-5.
22. Koup, R.A., et al., *Priming immunization with DNA augments immunogenicity of recombinant adenoviral vectors for both HIV-1 specific antibody and T-cell responses.* PLoS One, 2010. **5**(2): p. e9015.
23. Vasan, S., et al., *In Vivo Electroporation Enhances the Immunogenicity of an HIV-1 DNA Vaccine Candidate in Healthy Volunteers.* PLoS One, 2011. **6**(5): p. e19252.
24. Sandstrom, E., et al., *Broad immunogenicity of a multigene, multiclade HIV-1 DNA vaccine boosted with heterologous HIV-1 recombinant modified vaccinia virus Ankara.* J Infect Dis, 2008. **198**(10): p. 1482-90.
25. Bakari, M., et al., *Broad and potent immune responses to a low dose intradermal HIV-1 DNA boosted with HIV-1 recombinant MVA among healthy adults in Tanzania.* Vaccine, 2011. **29**(46): p. 8417-28.
26. Dobano, C., et al., *Enhancement of antibody and cellular immune responses to malaria DNA vaccines by in vivo electroporation.* Vaccine, 2007. **25**(36): p. 6635-45.
27. Widera, G., et al., *Increased DNA vaccine delivery and immunogenicity by electroporation in vivo.* J Immunol, 2000. **164**(9): p. 4635-40.
28. Dupuis, M., et al., *Distribution of DNA vaccines determines their immunogenicity after intramuscular injection in mice.* J Immunol, 2000. **165**(5): p. 2850-8.
29. Hooper, J.W., et al., *Smallpox DNA vaccine delivered by novel skin electroporation device protects mice against intranasal poxvirus challenge.* Vaccine, 2007. **25**(10): p. 1814-23.
30. Zhang, X., et al., *Intramuscular immunization with a monogenic plasmid DNA tuberculosis vaccine: Enhanced immunogenicity by electroporation and co-expression of GM-CSF transgene.* Vaccine, 2007. **25**(7): p. 1342-52.
31. Medi, B.M. and J. Singh, *Skin targeted DNA vaccine delivery using electroporation in rabbits II. Safety.* Int J Pharm, 2006. **308**(1-2): p. 61-8.
32. Medi, B.M. and J. Singh, *Delivery of DNA into skin via electroporation.* Methods Mol Biol, 2008. **423**: p. 225-32.
33. Wang, S., et al., *The relative immunogenicity of DNA vaccines delivered by the intramuscular needle injection, electroporation and gene gun methods.* Vaccine, 2008. **26**(17): p. 2100-10.
34. Draghia-Akli, R., et al., *Parameters for DNA vaccination using adaptive constant-current electroporation in mouse and pig models.* Vaccine, 2008. **26**(40): p. 5230-7.
35. Hirao, L.A., et al., *Combined effects of IL-12 and electroporation enhances the potency of DNA vaccination in macaques.* Vaccine, 2008. **26**(25): p. 3112-20.
36. Hirao, L.A., et al., *Intradermal/subcutaneous immunization by electroporation improves plasmid vaccine delivery and potency in pigs and rhesus macaques.* Vaccine, 2008. **26**(3): p. 440-8.
37. Luckay, A., et al., *Effect of plasmid DNA vaccine design and in vivo electroporation on the resulting vaccine-specific immune responses in rhesus macaques.* J Virol, 2007. **81**(10): p. 5257-69.
38. Rosati, M., et al., *Increased immune responses in rhesus macaques by DNA vaccination combined with electroporation.* Vaccine, 2008. **26**(40): p. 5223-9.
39. Otten, G., et al., *Enhancement of DNA vaccine potency in rhesus macaques by electroporation.* Vaccine, 2004. **22**(19): p. 2489-93.
40. Cristillo, A.D., et al., *Persistent antibody and T cell responses induced by HIV-1 DNA vaccine delivered by electroporation.* Biochem Biophys Res Commun, 2008. **366**(1): p. 29-35.

41. Brave, A., et al., *Biodistribution, persistence and lack of integration of a multigene HIV vaccine delivered by needle-free intradermal injection and electroporation.* Vaccine. **28**(51): p. 8203-9.
42. Dorrell, L., et al., *Safety and tolerability of recombinant modified vaccinia virus Ankara expressing an HIV-1 gag/multiepitope immunogen (MVA.HIVA) in HIV-1-infected persons receiving combination antiretroviral therapy.* Vaccine, 2007. **25**(17): p. 3277-83.
43. Gilbert, P.B., et al., *Long-term safety analysis of preventive HIV-1 vaccines evaluated in AIDS vaccine evaluation group NIAID-sponsored Phase I and II clinical trials.* Vaccine, 2003. **21**(21-22): p. 2933-47.
44. Jaoko, W., et al., *Safety and immunogenicity of recombinant low-dosage HIV-1 A vaccine candidates vectored by plasmid pTHr DNA or modified vaccinia virus Ankara (MVA) in humans in East Africa.* Vaccine, 2008. **26**(22): p. 2788-95.

APPENDIX 10: PROTOCOL VERSION 2.0 LISTING OF CHANGES

New text is indicated by the use of yellow highlight. Where it is necessary to aid understanding, the text that has been removed is marked with a strike through it.

The following table lists the pages and sections in which changes have been made, and the reason for the change to create v2.0.

Page	Text/section	Reason
1	Study Title	To reflect the addition of CN54 rgp140/GLA-AF to the MVA boosting
1	Protocol date	To reflect the date of the amended protocol which also appears in the header
1	Clinical trial numbers	To indicate the trial registration on Clinical Trials.gov and the Pan African Clinical Trials Registry (PACTR)
1 & 3	MUHAS Deputy Chief Investigator	Professor Kisali Pallangyo has been added as Deputy Chief Investigator
1 & 2	MUHAS co-sponsor	To indicate that Prof. Muhsin Aboud no longer represents MUHAS as co-sponsor. MUHAS is now represented by the Vice-Chancellor, Prof. Ephata E. Kaaya, as contractor
1	NIMR-MMRC	To reflect the change of name from MMRP to NIMR-Mbeya Medical Research Center (NIMR-MMRC). The name has been changed throughout the whole document.
4	List of Co-investigators	Dr. Emmanuel Kapesa has been added to the list of co-investigators at NIMR-MMRC.
6	Consultancies	Dr. Mary Marovich is no longer based at MHRP
6	Trial Pharmacists	The trial pharmacists' qualifications have been added
7	Data management/data management mentors	Maria Patricia Goncalves has been added as data manager for CISPOC and Dr. Sue Fleck as Data Management mentor for MRC-CTU
9	External Monitor	Dr. Kathleen Walker is no longer directly involved in monitoring
9-10	Vaccine suppliers/contacts	To indicate SMI as recipient of MVA donated by WRAIR and information about donation of CN54rgp140 and GLA-AF by Imperial College. Tina Tong has been added as the contact person at MHRP in relation to MVA product. Roger Tatoud is no longer involved in the distribution of CN54rgp140 and GLA-AF. The product will be donated to SMI for distribution to the sites.
10	Period for SAE and important AE notification period	To harmonise to 24 hours reporting throughout the protocol.
11-13	Contents	To reflect changes to trial Committees' names. Appendix 3A2 has been added to reflect the shorter version of the PIS/IC required by National Health Bioethics Committee in Mozambique and Appendix 10 has been included to list all protocol changes.
15	Abbreviations and glossary	To include SUSAR and TMT
17	Section 1.1.1	To reflect the factorial design and addition of CN54 rgp140/GLA-AF to the MVA boosts
17	Section 1.1.2	To include additional information about schedule, doses and routes of immunisation with CN54rgp140/GLA-AF in Table 1.
18	Section 1.1.3	To update the primary objectives including the safety and immunogenicity of CN54rgp140/GLA-AF of

18-19	Section 1.1.4	To include additional immunogenicity outcomes of interest
21	Section 1.1.8	To include the second randomisation in the flow diagram
25	Section 2.2.2	To update justification for DNA dose and delivery
26-27	Section 2.3 and 2.3.1	To include the rationale and hypothesis for the second randomisation reflecting the addition of CN54rgp140/GLA-AF to the MVA boost
28	Section 2.4.2	To include additional information on the safety of DNA and MVA vaccines as requested by the National Health Bioethics Committee requirements in Mozambique
28-29	Section 2.4.3	To add the description of CN54rgp140 and GLA-AF products
29	Section 2.4.4.3	To specify the site of CMDR-MVA immunisation
30	Section 2.4.4.4	To describe formulation procedures, route and site of CN54rgp140/GLA-AF immunisation
30	Section 2.4.5	To include CN54rgp140/GLA-AF
31	Section 3.1.2	To add the RV262 trial as this trial is currently ongoing at NIMR-MMRC
35	Section 4.2	To specify that only grade II or III AV block would be considered as exclusion criteria unless a 1st degree AV block is clinically significant
35-36	Section 4.3.2	To reflect current recruitment practices at Mbeya
36	Section 4.3.3	To reflect current recruitment procedures at Maputo, including a description on the evaluation of substance abuse and use of glucocorticoids as this was a request from the National Health Bioethics Committee requirements in Mozambique.
37-38	Section 4.4	To update the informed consent process and the procedures to follow at the end of the study relating to photo ID card and certificate on HIV vaccine trial participation as requested by the National Health Bioethics Committee requirements in Mozambique
39	Section 5.2	To provide details of the randomisation procedures and to accommodate the second randomisation.
40	Section 5.4	To reflect TMG as the committee involved in the review of co-enrolments
40	Section 5.5	To update procedures for dealing with replacements
41	Section 6.1	To include CN54rgp140/GLA-AF in the description of vaccine products
41-42	Section 6.2	To include CN54rgp140 and GLA-AF in the procedures for administration of vaccines
43-44	Section 6.3	To include information on supply, storage, composition and labelling of CN54 rgp140/GLA-AF
44	Section 6.3.1	To update details on inventory and transport of products from Dar es Salaam site to Mbeya site
45	Section 6.3.2	To include CN54rgp140/GLA-AF in the dispensing records and disposal of unused vaccines
46	Section 6.4	To update immunisation schedule to reflect second randomisation and include CN54rgp140/GLA-AF in Table 4
47	Section 6.5 and 6.6	To update correct names of relevant committees.
48	Section 6.8	To reflect current practice in Maputo on the use of HIV PCR in case of discordant ELISA results
48	Section 6.8.1	To indicate the use of the HIV trial participation card in case volunteers desire to be tested for HIV
48-49	Section 6.8.2	To reflect the requirements by the National Health Bioethics Committee in Mozambique on the procedures for the management of HIV, Syphilis and Hepatitis B
50	Section 7.1.2	To update visit windows reflecting second randomisation and timing of primary endpoint

51	Section 7.1.3	To update labelling procedures followed at Maputo
51	Section 7.2.1	To include ECG as part of the examinations at screening
52	Section 7.3.3	To reflect current practices at sites
54-55	Section 7.4.2	To specify immunogenicity endpoints for first and second randomisations and provide details of other immune responses which will be measured
56	Section 7.5.3	To specify that only grade II or III AV block would be considered as a significant finding
56	Section 7.5.4.1	To update procedures for Troponin I testing
58	Section 7.5.6	To reflect procedures for dealing with pregnancies and availability of contraception as requested by the National Health Bioethics Committee in Mozambique. Name of reporting committee was updated
58	Section 7.5.7	To update procedures for SAE reviewing and reporting
59	Section 7.6	To avoid duplication in the procedures for safety reporting in sections 7.6 and sections 8.3 and 8.4, the latter 2 sections have been rationalised into section 7.6
60	Section 7.8.1	To clarify the nature of the sub-study collaboration between MMRC and the Dar es Salaam and Maputo groups and need for ethics approval prior to involvement of volunteers and researchers as requested by the National Health Bioethics Committee requirements in Mozambique
61	Section 7.8.5	To indicate that ethics committee approval will be sought in relation to the structured questionnaires and procedures for recruiting volunteers
62	Section 8.1	To update definitions and classifications for safety reporting
62	Section 8.1.1	To update the definition of a grade 4 event
64	Section 8.2	To update the procedures for reporting adverse events which require expedited reporting by centres in relation to time frames and relevant committees
64	Section 8.2.1	To update procedures at the centres for reporting expedited events particularly in relation to the contact list
65	Section 8.2.2	To indicate that consent withdrawals/dropouts will also be reported to local IRBs as requested by the National Health Bioethics Committee requirements in Mozambique
67	Section 8.3 and 8.4	As explained above, the criteria for withdrawal or discontinuation and the criteria for pausing the study (whole sections were deleted) have been condensed into section 7.6
68	Section 9.2.1.1	To describe the primary immunogenicity endpoints relating to the first and second randomisation
68	Section 9.2.2.1	To include details of the secondary and the exploratory immunogenicity endpoints
69	Section 9.3	To update sample size calculations and associated assumptions for first (60 per group) and second (90 per group) randomisation
70-71	Section 9.5, 9.5.1 and 9.5.2	To update the description of populations and variables which will be included in the analyses in relation to the first and second randomisation.
76	Section 11.1	To reflect relevant individuals and committees involved
76	Section 11.2	To update details of safety calls during the study
77	Section 11.6	To update details of plans for ensuring quality of data
81	Section 13.2	To clarify that the volunteer will be covered by an insurance if proven that the adverse events is associated with the study product as requested by the National Health Bioethics Committee requirements in Mozambique. To update the sponsor responsibilities
83-84	Section 15.1 and 15.2	To update the name of relevant committees, composition and role of the trial management team

84	Section 15.3	To update the composition and role of the TaMoVac steering committee
85	Section 15.5	To update the role of TMG in the context of DSMB procedures for safety reporting
86	Section 16	To reflect the current timeframe for receipt of comments on potential manuscripts
87	Section 17	To reflect the committee responsible for preparing protocol amendments
91-92	Appendix 1	To include CN54rgp140/GLA-AF
93-94	Appendix 2	To include CN54rgp140/GLA-AF and update on current practices for ECG, Troponin I testing and blood volumes for immunogenicity assays
95	Appendices 3A1 and 3A2	To include a shorter version of the PIS/IC as Appendix 3A2 following the request by the National Health Bioethics Committee of Mozambique to shorthen the PIS/IC. The PIS/IC for Tanzania was re-labelled as Apppendix 3A1.

Summary of changes from Tamovac II protocol version 1.0 to version 1.1 following review by the National Health Bioethics Committee of Mozambique (page numbers refer to the original protocol dated 12 March 2012)

Page	Section	Reason
28	Section 2.4.2	To include additional information on the safety of DNA and MVA vaccines
31	Section 4.2	To add explanation on how participants will be assessed in terms of substance abuse, asthma and use of corticosteroids in points 6 and 9.
33	Section 4.4	To update the procedures to follow at the end of the study relating to photo ID card and certificate on HIV vaccine trial participation
43	Section 6.8.1	To indicate the use of the HIV trial participation card in case volunteers desire to be tested for HIV
43	Section 6.8.2	To include procedures for the management of HIV, Syphilis and Hepatitis B
52	Section 7.5.6	To include procedures for dealing with pregnancies and availability of contraception and counselling
52	Section 7.6	To indicate that all consent withdrawals/dropouts that occur during the study should be notified to the National Health Bioethics Committee of Mozambique.
53	Section 7.8.1	To clarify the need for ethics approval prior to involvement of volunteers and researchers in the substudy.
82	Section 13.2	To clarify that the volunteer will be covered by an insurance if proven that the adverse events is associated with the study product.
-	PIS/IC	To shorten the PIS/IC

Summary of changes to the PIS/IC, Tanzania

Page	Change	Reason
1	MMRP logo	To reflect the change in the name of MMRP to MMRC
1	Protocol version and dates	To reflect amendment from version 1.0 to version 2.0 and the date of amendment
1	Study Title	To reflect the addition (or not) of CN54 rgp140/GLA-AF to the MVA boosting
1	1st and 3rd paragraph	To reflect the addition (or not) of a protein (CN54 rgp140/GLA-AF) to the vaccination regimen and change of name to from MMRP to MMRC. The name has been changed throughout. Doses of DNA vaccine have been corrected
2	Purposes	To explain the purpose of adding a protein (CN54 rgp140/GLA-AF) to the vaccination regimen and to highlight the collaboration between Tanzania and Mozambique in capacity development
2-3	The vaccines	To update vaccine composition, safety of vaccine products, including the addition of CN54 rgp140/GLA-AF to the vaccination regimen
3	The Trial	To indicate that the vaccine has been approved for investigational use. Information related to Mozambique deleted. To reflect the second randomization
4	Table	To indicate the addition (or not) of the protein (CN54 rgp140/GLA-AF) to the MVA vaccinations at visits 11 (week 24) and 14 (week 40)
5-6	Vaccination groups and procedures for enrolement and follow up	To reflect the addition (or not) of the protein (CN54 rgp140/GLA-AF) to the MVA vaccinations
7	HLA testing	To simplify the information given on HLA testing
9	Risks	To explain the risks associated with the protein and adjuvant (CN54 rgp140/GLA-AF)
9	Risks	To simplify the information on the risks related to HIIV exposure
12	Compensation for time and inconvenience	To delete information related to compensation in Mozambique
13	Contacts	To update the list of contacts
15	Consent	To include consent to store samples

Summary of changes to the PIS/IC, Mozambique

Page	Change	Reason
1	MMRP logo	To reflect the change in the name of MMRP to MMRC
1	Protocol version and dates	To reflect amendment from version 1.0 to version 2.0 and the date of amendment
1	Study Title	To reflect the addition (or not) of CN54 rgp140/GLA-AF to the MVA boosting
1	Paragraphs 1 and 3	To reflect the addition (or not) of a protein (CN54 rgp140/GLA-AF) to the vaccination regimen and change of name to from MMRP to MMRC. The name has been changed throughout. Doses of DNA vaccine have been corrected
2	Purposes	To explain the purpose of adding a protein (CN54 rgp140/GLA-AF) to the vaccination regimen and to highlight the collaboration between Tanzania and Mozambique in capacity development

2	The vaccines, paragraph 1	To explain the results of Tamovac I in which a greater immune response was observed by adding a protein (CN54 rgp140/GLA-AF) to the vaccination regimen and to indicate that in this study the protein will be given at the time of MVA vaccination
2	The vaccines, last paragraph	To explain what an MVA vaccine is
3	The vaccines, paragraph 2	To explain what the protein and adjuvant are (CN54 rgp140/GLA-AF)
3	The Trial, paragraph 1, 2 and 3	To indicate that the vaccine has been approved for investigational use. Information related to Tanzania deleted
3	The Trial, paragraph 5	To reflect the second randomization
4	Table	To indicate the addition (or not) of the protein (CN54 rgp140/GLA-AF) to the MVA vaccinations at visits 11 (week 24) and 14 (week 40)
5	Vaccination groups	To reflect the addition (or not) of the protein (CN54 rgp140/GLA-AF) to the MVA vaccinations and/or the placebo
6	Paragraphs 4 and 6	To reflect the addition (or not) of the protein (CN54 rgp140/GLA-AF) to the MVA boost
9	Paragraphs 3 and 4	To explain the risks associated with the protein and adjuvant (CN54 rgp140/GLA-AF)
